# Feeding-induced muscle mTORC1 signaling regulates postprandial protein synthesis and endurance but not muscle size

**DOI:** 10.1172/jci.insight.210523

**Published:** 2026-09-10

**Authors:** Samuel C. Lapp, Krystle C. Kalafut, Madi Y. Cissé, Khaled Tighanimine, Dean M. Rosenthal, Will Doxsey, Sheng Hui, Karen E. Inouye, Claire E. Morrow, Yann Cormerais, Brendan D. Manning

**Affiliations:** 1 Department of Molecular Metabolism, Harvard T.H. Chan School of Public Health, Boston, Massachusetts, USA.; 2 Department of Cell Biology, Harvard Medical School, Boston, Massachusetts, USA.

**Keywords:** Endocrinology, Muscle biology, Signal transduction

## Abstract

Activation of the mechanistic target of rapamycin (mTOR) complex1 (mTORC1) promotes muscle protein synthesis, mass, and function. Muscle mTORC1 can be activated by feeding and contraction. Here, muscle mTORC1 signaling, protein synthesis, mass, and function are characterized in a genetic mouse model that separates these 2 major modes of muscle mTORC1 regulation. AKT signaling is required for feeding-induced muscle mTORC1 signaling and protein synthesis, and mice expressing a mutant of tuberous sclerosis complex 2 (TSC2) that cannot be phosphorylated by AKT specifically in skeletal muscle (SkM-TSC2-5A) attenuate these effects of feeding. Despite this loss of postprandial protein synthesis, SkM-TSC2-5A mice have similar muscle and myofiber size compared with SkM-TSC2-WT mice. SkM-TSC2-5A mice maintain normal muscle mTORC1 activation in response to contraction and exhibit no differences in atrophy-related gene expression or ribosomal content. SkM-TSC2-5A mice exhibit improved maximal endurance capacity without changes in muscle contractile function. This phenotype occurs without alterations in muscle glycogen content or myofiber type, but it does coincide with a modest increase in muscle mitochondrial content. Therefore, AKT-mediated phosphorylation of TSC2 is required for postprandial mTORC1 activation and the induction of protein synthesis; however, these are dispensable for the development and maintenance of muscle mass in sedentary mice.

## Graphical abstract

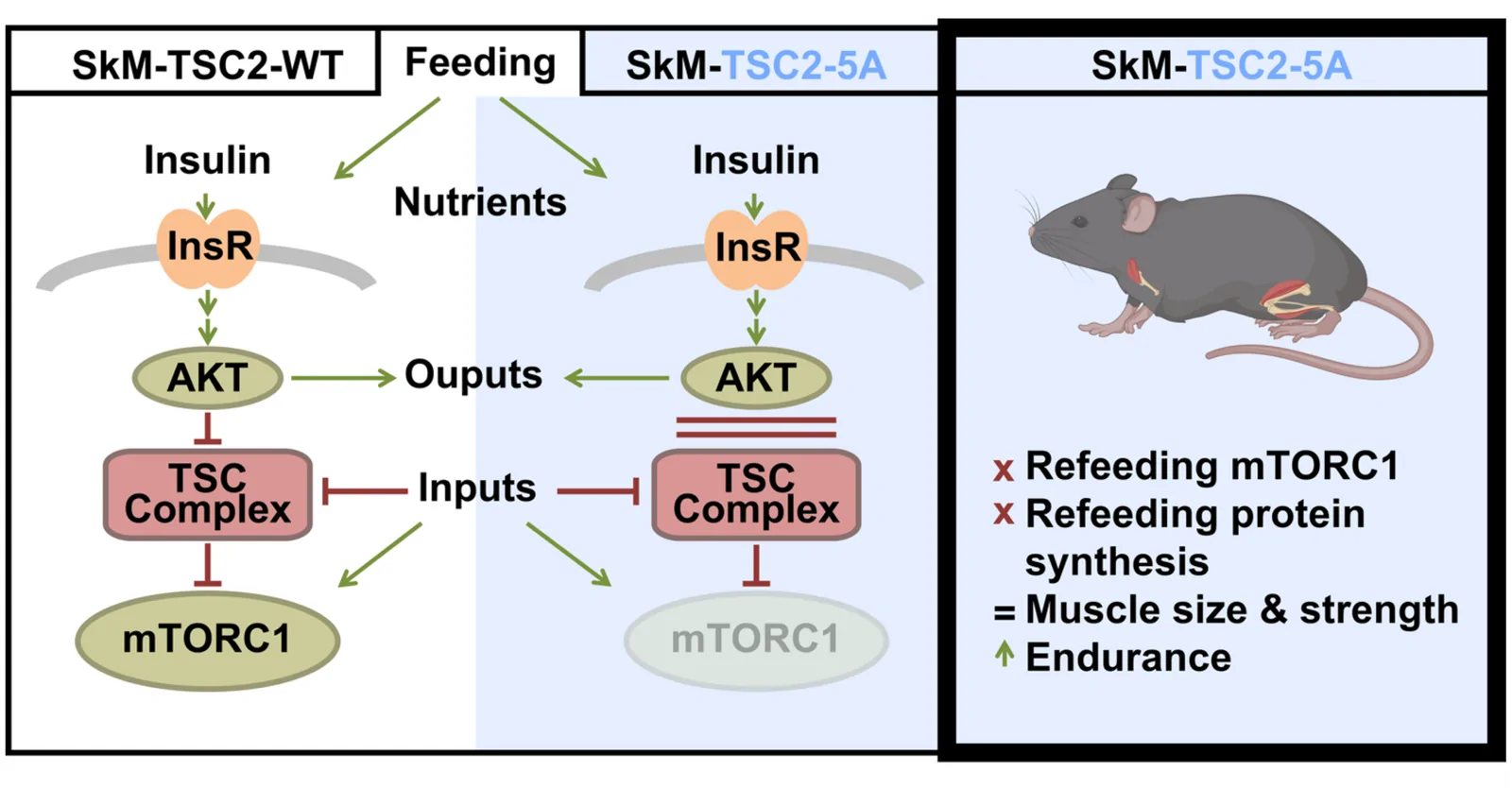

## Introduction

Skeletal muscle is a crucial site for metabolic homeostasis (1, 2). It is the largest insulin-sensitive organ in healthy adults, the primary amino acid depot in mammals, and critical for locomotion (1–3). Skeletal muscle is uniquely plastic in response to diverse stimuli, including endocrine and growth factors, nutrients, and contraction, influencing muscle growth in a time- and intensity-dependent manner (1, 2, 4, 5). Dysregulation in signaling from these stimuli from inactivity, malnutrition, aging, cancer, or genetic disruption results in skeletal muscle weakness or atrophy (6–15). Therefore, understanding the dominant factors regulating skeletal muscle mass and function will inform us on better ways to address disease- or lifestyle-related muscle loss.

Food intake provides a complex input of endocrine factors and nutrients that stimulate anabolism and suppress catabolism in skeletal muscle (3, 16, 17). These signals converge on the mechanistic target of rapamycin (mTOR) complex 1 (mTORC1) in skeletal muscle to regulate, in part, this transition to net anabolism (16, 18). mTORC1 is a conserved regulator of cellular growth and critical for skeletal muscle mass (11–15, 19, 20). This protein complex consists of mTOR, lethal with SEC13 protein 8 (LST8), and regulatory-associated protein of mTOR (RAPTOR) as its essential core components (18). Ras homolog enriched in brain (RHEB) is a small GTPase that, when GTP-loaded, binds to and activates mTORC1 (21–23). Amino acids activate mTORC1 in a Rag GTPase-dependent manner by prompting the interaction of mTORC1 with GTP-bound RHEB (24–26). Hence, local amino acid availability is essential for mTORC1 activation.

The GTP/GDP-loading state of RHEB is regulated by exogenous endocrine and growth factors, including insulin, through the tuberous sclerosis complex (TSC) protein complex (21). The TSC complex, consisting of TSC1, TSC2, and TBC1D17, serves as a GTPase-activating protein (GAP) for RHEB, thus decreasing RHEB-GTP levels and inhibiting its activation of mTORC1 (21). Therefore, growth factor activation of mTORC1 is thought to occur primarily through preventing the TSC complex from acting as a RHEB-GAP. Following insulin stimulation, the protein kinase AKT is activated and directly phosphorylates TSC2 on at least 5 sites, spatially separating it from RHEB and allowing RHEB-GTP to accumulate and activate mTORC1 (21, 27–29). However, nearly all these regulatory mechanisms upstream of mTORC1 are derived from studies in cell culture models.

Unique among cell types, skeletal muscle cells comprising myofibers also activate mTORC1 by contraction (4, 30). While this regulation is less well understood than that coming from insulin, AKT and TSC2-dependent and -independent mechanisms have been proposed (15, 31–33). Thus, signals from endocrine factors, nutrients, and contraction may converge on mTORC1 in skeletal muscle, making the functional dissection of any individual input challenging.

This current study seeks to isolate the role of endocrine signaling to mTORC1 in response to feeding and the physiological role of feeding in determining skeletal muscle mass and function. For this purpose, a skeletal muscle–specific mouse model expressing a mutant of TSC2 (SkM-TSC2-5A) that lacks the inhibitory AKT-mediated phosphorylation sites on TSC2, specifically in skeletal muscle, was generated. The TSC complex in the muscle of these mice is insensitive to inhibition by AKT but leaves other AKT functions and AKT-independent inputs to mTORC1 regulation intact. The SkM-TSC2-5A model demonstrates that these phosphorylation sites are required for postprandial induction of mTORC1 activation and protein synthesis in skeletal muscle. However, loss of postprandial protein synthesis did not reduce bodyweight, lean mass, muscle weight, or myofiber size in sedentary mice. Instead, this suggests that muscle size is likely maintained by alternative signals to mTORC1, such as contraction, which we show to be unaffected in SkM-TSC2-5A mice. Functionally, AKT signaling to mTORC1 is dispensable for the contractile properties of muscle, but its loss enhances maximal endurance capacity. This endurance phenotype is not due to changes in muscle glycogen content or fiber type proportions but is associated with a modest increase in muscle mitochondrial content. The findings described suggest that exogenous endocrine factor signaling induces postprandial mTORC1 activation and protein synthesis but that neither influence muscle mass nor contractile function in sedentary mice.

## Results

### Feeding induces postprandial protein synthesis in skeletal muscle through activation of AKT and mTORC1.

There are multiple possible routes to muscle mTORC1 activation from feeding and contraction (Figure 1A). Studying cell signaling in mammalian tissues is challenging, especially in metabolic tissues such as skeletal muscle, where induction of the AKT-mTOR pathway and many other pathways is highly sensitive to the naturally differential timing of food intake. After piloting different conditions, including ad libitum feeding and daytime fast followed by night-time feed, we settled on an overnight fast followed by a 2-hour refeed as the best way to synchronize feeding behavior in mice to get more uniform, feeding-specific induction of the pathways of interest in skeletal muscle. Therefore, we use this paradigm throughout this study and recognize the caveats that accompany it. To assess how feeding stimulates mTORC1 signaling and protein synthesis in skeletal muscle, mice were fasted overnight and refed for 2 hours with or without an AKT inhibitor. Refeeding induced the activating phosphorylation of AKT and, with a trend toward significance, the AKT-mediated inhibitory phosphorylation of TSC2 in the quadriceps muscle (Figure 1, B and C, and Supplemental Figure 1A; supplemental material available online with this article; https://doi.org/10.1172/jci.insight.210523DS1). This induction was associated with stimulation of mTORC1 signaling, as scored by phosphorylation of ribosomal protein S6 kinase 1 (S6K1) at T389, ribosomal protein S6 (S6) at S240/244, and eukaryotic translation initiation factor 4E binding protein 1 (4E-BP1) (via electrophoretic shift) (Figure 1, B and D, and Supplemental Figure 1B). Pretreatment of mice with an AKT inhibitor reduced feeding-induced muscle TSC2 phosphorylation and mTORC1 signaling to that of the fasted state (Figure 1, B–D, and Supplemental Figure 1, A and B). Similarly, pretreatment with either an AKT inhibitor or the mTORC1 inhibitor rapamycin reduced both mTORC1 signaling and rates of postprandial protein synthesis to that of the fasted state (Figure 1, E and F), as determined by puromycin incorporation over the final 30 minutes of refeeding (34). Together, these data demonstrate that acute refeeding induces mTORC1 signaling and protein synthesis in a manner that is dependent on AKT.

### AKT-mediated phosphorylation of TSC2 is required for feeding-induced activation of mTORC1.

AKT activates mTORC1, at least in part, through TSC2 phosphorylation in vitro and likely also in skeletal muscle (Figure 1A) (27, 35, 36). To assess the physiological importance of this regulation more clearly, a genetic mouse model was generated that lacks the ability of AKT to phosphorylate TSC2 specifically in adult skeletal muscle. Two lines of knock-in/knock-out mice were generated using the striated muscle-specific *HSA* (actin alpha 1 [*ACTA1*])-*Cre* promoter (Figure 2A) (37). In brief, mice with floxed endogenous mouse *Tsc2* (*mTsc2*) (38) were crossed with mice featuring either a human *TSC2* (*hTSC2)-WT* or a *hTSC2-5A* phosphomutant under control of a lox-stop-lox cassette at the *Rosa26* (R26) locus, described previously (36). *R26-hTSC2^WT/WT^ Tsc2^fl/fl^* and *R26-hTSC2^5A/5A^ Tsc2^fl/fl^* were crossed with *HSA-Cre^+/–^* mice to conditionally knock out endogenous *mTsc2* while complementing the loss with either *hTSC2-WT* or *hTSC2-5A* transgene expression specifically in *HSA*-expressing differentiated muscle, resulting in SkM-TSC2-WT and SkM-TSC2-5A mice (37). As confirmation of proper Cre-dependent recombination at these 2 loci, transcripts of the *hTSC2* transgenes are detected only in the skeletal muscle of *Cre^+^* SkM-TSC2-WT and -5A mice (Figure 2B). Reciprocally, endogenous *mTsc2* transcripts were greatly decreased in *Cre*-expressing skeletal muscle relative to floxed controls. In contrast, both *Cre^+^* and *Cre^–^* mice express similar levels of *mTsc2* transcripts in control tissues, including the cardiac muscle and liver (Figure 2B). Given the documented expression of *HSA-Cre* in striated muscles (37), which include both skeletal and cardiac muscle, relative recombination was also assessed by measuring TSC2 phosphorylation at one of the sites mutated in the SkM-TSC2-5A mice, T1462, in the gastrocnemius and heart (Figure 2, C–E). SkM-TSC2-5A mice completely lack phosphorylation of TSC2 at T1462 in skeletal muscle but maintain comparable phosphorylation in the heart, confirming that expression of endogenous WT TSC2 is unchanged in the heart and that this model is skeletal muscle specific.

When fed ad libitum, SkM-TSC2-5A mice trended toward higher levels of skeletal muscle AKT activation compared with SkM-TSC2-WT mice (Figure 2, F and G, and Supplemental Figure 2, A and B). Despite this, muscles from both male and female SkM-TSC2-5A mice showed a decrease in mTORC1 signaling (Figure 2, F and H, and Supplemental Figure 2, A and C). While there was no difference between male SkM-TSC2-WT and -5A mice in skeletal muscle AKT, S6, or 4E-BP1 phosphorylation following a 12-hour daytime fast, SkM-TSC2-5A mice had significantly elevated phosphorylation of AKT and abrogation of postprandial S6 and 4E-BP1 phosphorylation following 2 hours of refeeding (Figure 2, I–K). The increased AKT activation in SkM-TSC2-5A muscles likely reflects loss of the well-documented mTORC1-mediated feedback inhibition of AKT signaling (39–41).

Given that feeding increases nutrient availability, metabolomic analyses was performed on skeletal muscle following the same fasting and refeeding paradigm to determine whether major changes in skeletal muscle metabolites might underlie the dampened mTORC1 signaling in SkM-TSC2-5A muscle. Feeding induced an increase in some, but not all, amino acids present in the skeletal muscle, and SkM-TSC2-5A mice had similar or elevated levels of muscle amino acids compared with SkM-TSC2-WT mice (Supplemental Figure 2D). Both glycolytic and TCA cycle intermediates, as well as free fatty acid species, were also similar in the muscles of SkM-TSC2-WT and -5A mice under both fasting and feeding conditions (Supplemental Figure 2, E and F). Together, these data demonstrate that skeletal muscle–intrinsic AKT-mediated phosphorylation of TSC2 is necessary for postprandial mTORC1 activation without gross changes in intramuscular nutrients or metabolites that might indirectly influence mTORC1 signaling.

### AKT-mediated phosphorylation of TSC2 is required for postprandial protein synthesis in skeletal muscle.

Given that feeding activates postprandial protein synthesis in skeletal muscle in an AKT- and mTORC1-dependent manner (Figure 1), we next determined whether AKT-mediated phosphorylation of TSC2 is required for postprandial protein synthesis. Mice were fasted overnight and refed for 2 hours, with protein synthesis measured by puromycin incorporation over the final 30 minutes (34). Male SkM-TSC2-WT mice aged 2 and 8 months displayed a respective ~54% and ~25% increase in postprandial muscle protein synthesis, which was completely abolished in SkM-TSC2-5A mice (Figure 3, A and B, and Supplemental Figure 3, A and B). Similarly, female 2-month-old SkM-TSC2-WT mice demonstrated a ~64% increase in postprandial muscle protein synthesis that was abrogated in their -5A counterparts (Figure 3, C and D). Female 8-month-old SkM-TSC2-5A mice showed a reduction in muscle protein synthesis relative to SkM-TSC2-WT mice under both fasted and refed conditions, but feeding failed to induce a significant increase in muscle protein synthesis in either genotype (Supplemental Figure 3, C and D). The greater induction of postprandial protein synthesis in the muscles of 2-month-old mice may be indicative of the rapid growth occurring at this age (4, 11). At 6 months of age, there was no difference between male SkM-TSC2-WT and -5A mice in respiratory exchange ratio (RER) with ad libitum refeeding following the same fasting paradigm as above, suggesting that there are likely similar feeding habits and nutrient absorption between genotypes (Supplemental Figure 3E). These data demonstrate that AKT-mediated phosphorylation of TSC2 to activate mTORC1 is required for the induction of postprandial protein synthesis in skeletal muscle.

### AKT-mediated phosphorylation of TSC2 has substrate specific effects downstream of skeletal muscle mTORC1 signaling.

mTORC1 has downstream substrates that fall into 1 of 4 categories: RHEB-dependent, rapamycin sensitive (e.g., S6K1); RHEB-dependent, partially rapamycin sensitive (e.g., 4E-BP1); RHEB-dependent, rapamycin resistant (e.g., unc-51 like autophagy activating kinase 1 [ULK1]); and RHEB-independent, rapamycin resistant (e.g., transcription factor EB [TFEB]) (42, 43). Therefore, it is critical to understand how specific inputs to mTORC1, like feeding, can selectively affect downstream substrates. Given that AKT-mediated phosphorylation of TSC2 should only affect the RHEB-dependent targets of mTORC1, the effects of feeding on the phosphorylation of S6K1, 4E-BP1, ULK1, and TFEB — representative of these 4 categories — were determined in the skeletal muscle of fasted and refed SkM-TSC2-WT and -5A mice. As seen above, the feeding-induced phosphorylation of skeletal muscle S6K1 and 4E-BP1 were abrogated in both male and female SkM-TSC2-5A mice (Figure 4, A, B, and E, and Supplemental Figure 4A). The feeding-induced mTORC1-mediated phosphorylation of skeletal muscle ULK1 at S757 was also significantly decreased in SkM-TSC2-5A mice (Figure 4, A, C, and F, and Supplemental Figure 4A). In contrast, the mTORC1-mediated phosphorylation of the TFEB transcription factor, which is dependent on amino acid signaling through the Rag proteins and independent of TSC-RHEB signaling (44), was not impaired in SkM-TSC2-5A muscle but was actually elevated, especially in male mice (Figure 4, A, D, and G, and Supplemental Figure 4A). This difference in TFEB phosphorylation is consistent with increased TSC2 activity yielding a decrease in RHEB-dependent mTORC1 signaling in SkM-TSC2-5A muscles, as the RHEB-mediated activation of mTORC1 has been found to hinder the ability of mTORC1 to regulate TFEB (45–47). These data suggest that AKT-mediated phosphorylation of TSC2 in skeletal muscle is required specifically for postprandial RHEB-mTORC1 signaling to downstream substrates but not regulation of RHEB-independent substrates of mTORC1, like TFEB.

### Loss of postprandial mTORC1 activation and protein synthesis does not decrease muscle mass.

Unexpectedly (36), the loss of postprandial muscle mTORC1 activation and protein synthesis in SkM-TSC2-5A mice did not reduce bodyweight, lean mass, muscle weight, or myofiber size (Figure 5 and Supplemental Figure 5). Male SkM-TSC2-5A mice exhibited a modest increase in bodyweight over time compared with their WT counterparts, while female mice did not show genotype-specific differences (Figure 5, A and B, and Supplemental Figure 5, A and B). This trend was also observed in measurements of lean mass over time (Figure 5, C and D, and Supplemental Figure 5, C and D), while neither sex showed a genotype specific effect on fat mass (Supplemental Figure 5, E and F). Neither male nor female SkM-TSC2-5A mice showed differences in skeletal or cardiac muscle weights compared with their WT counterparts at either 4 months (Figure 5, E and F) or 8 months (Supplemental Figure 5, G and H). In the plantaris muscle (Figure 5G), there was no difference measured in the average myofiber size (Figure 5H), nor was there a difference in the specific size distributions of Type IIB (Figure 5I), Type IIX (Figure 5J), or Type IIA (Figure 5K) myofibers. Analysis of a uniform area of red gastrocnemius (Figure 5G) also showed no difference in average myofiber size (Figure 5L), nor any gross differences in Type IIB (Figure 5M), Type IIX (Figure 5N), or Type IIA (Figure 5O) fiber size distributions. Individual mouse values are shown in Supplemental Figure 5, I–N. Only a slight shift toward larger IIB fibers in the SkM-TSC2-5A gastrocnemius was detected (Figure 5M). Type I fibers were not detected in the plantaris and showed no difference in size distribution within the gastrocnemius (Supplemental Figure 5, O and P). These data indicate that feeding-induced activation of mTORC1 and stimulation of protein synthesis in skeletal muscle following an overnight fast, both of which are defective in the SkM-TSC2-5A mice, does not influence muscle mass or myofiber size.

### AKT-mediated phosphorylation of TSC2 does not affect skeletal muscle expression of atrophy-related genes or RNA content.

Alterations in muscle mass can come from changes in the relative balance of protein synthesis and degradation (2). Atrophy-related genes encode proteins that induce muscle protein degradation and whose transcripts are elevated during muscle atrophy (48–51). The most relevant of these are the ubiquitin ligases F-box only protein 3 (Fbxo32, also known as Atrogin-1) and tripartite motif containing 63 (Trim63, also known as MuRF1), as well as growth arrest and DNA damage-inducible 45 (Gadd45a) (50, 52–54). However, no significant differences in transcript levels of any of these genes were detected between SkM-TSC2-WT and -5A muscles under fasting or ad libitum–fed conditions (Figure 6, A and B). These data suggest that a decrease in muscle protein degradation, at least due to differential expression of these common atrophy-related genes, is unlikely to explain the maintenance of muscle mass in the SkM-TSC2-5A mice.

Overall protein synthetic capacity and cellular growth are dictated by ribosomal content, which can be regulated by mTORC1 (55). Muscle mass scales has a positive, linear relationship with ribosomal content, which is considered reflective of total RNA content (4, 56, 57). Similar concentrations of RNA were measured in SkM-TSC2-WT and -5A skeletal muscle (Figure 6C), and both maintained the same linear relationship between RNA content and muscle tissue weight (Figure 6D; *P* = 0.7888). This suggests that loss of feeding-induced muscle mTORC1 signaling does not affect ribosomal content in skeletal muscle, implying that both genotypes have similar protein synthetic capacity in their muscles.

### AKT-mediated phosphorylation of TSC2 is dispensable for contraction-mediated mTORC1 activation in skeletal muscle.

While skeletal muscle growth requires mTORC1 activation, the relative contribution of upstream signals, including feeding and contraction, to growth-promoting mTORC1 signaling are less well understood. Since the SkM-TSC2-5A mice are defective in feeding-induced mTORC1 activation without effects on muscle mass, contraction-induced mTORC1 signaling was assayed in these mice. Thus, maximal intensity contractions (MIC) were elicited through direct electrical stimulation of the sciatic nerve (Figure 7A), as previously described (58). At 3 hours after contraction, when skeletal muscle mTORC1 signaling has been shown to peak in response to MIC (30), there was no difference in AKT activation, as previously described (59), or its phosphorylation of TSC2 between the contracted (MIC) and uncontracted contralateral (Cntrl) TA muscle from the same mouse in both males and females (Figure 7, B, C, and E, and Supplemental Figure 6, A and B). However, contraction potently activated mTORC1 in both sexes relative to the uncontracted contralateral muscle from the same animal (Figure 7, B, D, and F–J, and Supplemental Figure 6, A and B). Importantly, and in contrast to feeding, the phosphorylation of S6K1, S6, and 4E-BP1 in skeletal muscle following contraction was induced in SkM-TSC2-5A mice with no significant differences from their WT counterparts. These data suggest that contraction does not activate muscle mTORC1 through AKT-mediated phosphorylation of TSC2 and that the sustained ability of contraction to stimulate mTORC1 may be an alternative signal that contributes to the development and maintenance of normal muscle mass in SkM-TSC2-5A mice.

### Loss of AKT-mediated phosphorylation of TSC2 enhances maximal endurance capacity without altering contractile function.

In assays of muscle function, a slight increase in grip strength was detected in male, but not female, SkM-TSC2-5A mice (Figure 8A and Supplemental Figure 7A), prompting further analyses of muscular strength in male mice. Male mice underwent electrical stimulation of the tibial nerve to assess functional outcomes in the plantarflexors. There were no differences across stimulation intensities in maximal isometric torque produced in male SkM-TSC2-WT and -5A mice (Figure 8B and Supplemental Figure 7B), suggesting that there are no gross differences in skeletal muscle strength between genotypes. Inducible deletion of either mTOR or RAPTOR in skeletal muscle results in impaired contractility and delayed muscle relaxation after contraction (60, 61). However, no differences in the rates of muscle contraction and relaxation were detected between male SkM-TSC2-WT and -5A mice (Figure 8, C and D, and Supplemental Figure 7, C and D). In an inverted screen test, SkM-TSC2-5A mice exhibited similar relationships between hang time and bodyweight as their SkM-TSC2-WT counterparts (Figure 8E: *P* = 0.6434), but the SkM-TSC2-5A mice displayed an overall decrease in total hang time (Figure 8F). This prompted further investigation into fatigue resistance in SkM-TSC2-5A mice. In response to multiple, submaximal stimulations reported as the decline of force production as a percentage of maximum initial force, there were no significant differences in fatigue resistance between male SkM-TSC2-WT and -5A mice when assessed as the decline in force production over repeated submaximal stimulations (Figure 8, G–I, and Supplemental Figure 7E). Therefore, consistent with the lack of differences in muscle mass, the overall strength and contractile function of the muscle in SkM-TSC2-WT and -5A mice are similar.

We next assessed endurance capacity via run-to-exhaustion experiments as previously described (62). Mice ran at a consistent incline with periodic increases in treadmill speed until exhaustion. Interestingly, male SkM-TSC2-5A mice showed improved endurance capacity, as demonstrated by increased maximum speed, total run time, and cumulative distance run (Figure 8, J–L). However, this phenotype was not observed in female SkM-TSC2-5A mice (Supplemental Figure 7, F–H). Thus, parameters that might underlie this unexpected phenotype were measured in muscles from male mice.

### Improved endurance capacity in SkM-TSC2-5A mice is not associated with changes in skeletal muscle glycogen content.

During maximal exercise, skeletal muscle energy production becomes disproportionally dependent on glycogen (63), suggesting that increased glycogen content may explain differences in endurance capacity between SkM-TSC2-WT and -5A mice. In metabolic cage measurements, SkM-TSC2-5A mice exhibited a slightly higher, but not statistically significant, RER — particularly when having access to wheel running, perhaps indicative of higher carbohydrate utilization (Figure 9A). This prompted us to investigate alterations in glycogen and glucose metabolism. SkM-TSC2-5A mice have enhanced feeding-induced AKT activation in their skeletal muscle compared with SkM-TSC2-WT mice (Figure 2, G and J and Figure 9, B and C). GSK3β is a direct target inhibited by AKT that has been implicated in regulating glycogen content through its inhibition of glycogen synthase (35). Indeed, the AKT-mediated inhibitory phosphorylation of GSK3β was elevated during refeeding in SkM-TSC2-5A muscles compared with WT (Figure 9, B and D), which could theoretically result in increased glycogen synthesis in these muscles. However, SkM-TSC2-WT and -5A mice did not exhibit significant differences in glycogen content or GSK3β phosphorylation in muscles from ad libitum fed (Figure 9E and Supplemental Figure 8, A–C) or fasted (Figure 9, B, D, and F) mice. Thus, an increase in muscle glycogen content is unlikely to explain improvements in endurance capacity in SkM-TSC2-5A mice.

A primary function of insulin-stimulated AKT signaling in skeletal muscle is to promote glucose uptake and clearance from the blood (1). Therefore, we investigated glucose clearance and insulin sensitivity in SkM-TSC2-WT and -5A mice. There were no differences in either ad libitum or fasted blood glucose levels between male SkM-TSC2-WT and -5A mice (Supplemental Figure 8, D and E). Glucose tolerance tests (GTT) in both male and female mice also showed no differences between genotypes in glucose clearance (Supplemental Figure 8, F–I). Likewise, there were no differences in insulin sensitivity in insulin-tolerance tests (ITT) between genotypes in either male or female cohorts (Supplemental Figure 8, J–M). Together, we observe no overt differences in glucose metabolism dependent on AKT-mediated phosphorylation of TSC2 in skeletal muscle.

### SkM-TSC2-5A muscle display a moderate increase in mitochondrial content without changes in muscle fiber type.

Beyond glycogen content, skeletal muscle fiber type and mitochondrial content can alter endurance capacity (64–66). Skeletal muscle fiber types exist on a gradient between oxidative (I and IIA) and glycolytic (IIX and IIB), where a shift toward more oxidative fibers is associated with improved endurance capacity (65). Complete ablation of skeletal muscle mTORC1 signaling has been found to result in a shift toward type I and IIA fiber types (13, 61); therefore, fiber type identity was compared between muscles of SkM-TSC2-WT and -5A mice. Skeletal muscle gene expression for markers of oxidative (*Tnni1*, *Myh1*, *Myh2*) and glycolytic (*Tnni2*, *Myh4*) fiber types did not significantly differ between SkM-TSC2-WT and -5A mice (Figure 10, A and B). Furthermore, direct assessment of fiber type proportions showed no differences in the plantaris or red gastrocnemius (Figure 5G and Figure 10, C and D). These data indicate that fiber type composition is not significantly different between SkM-TSC2-WT and -5A mice and is, thus, unlikely to explain the alterations in endurance capacity observed.

While oxidative fiber types are associated with higher mitochondrial content, fiber type identity and mitochondrial content can be uncoupled (65). Therefore, we quantified mitochondrial content by comparing nuclear DNA (nDNA) content to mitochondrial DNA (mtDNA), as previously described (67). Using 2 distinct markers of mtDNA, a modest but significant 3%–4% increase in mtDNA/nDNA ratio was measured in SkM-TSC2-5A compared with WT muscle (Figure 10, E and F). While not a direct measurement of mitochondrial function, it is plausible that this modest increase in mitochondrial content could underlie, at least in part, the improved endurance capacity of SkM-TSC2-5A mice.

## Discussion

The SkM-TSC2-5A mice provide insights into the physiological regulation and function of skeletal muscle mTORC1, which is widely believed to be a key signaling node in the control of muscle growth and strength (4, 16). While the complete absence or maximal sustained activation of skeletal muscle mTORC1 — as achieved through muscle-specific genetic deletion of RAPTOR or TSC1, respectively — has dramatic effects on skeletal muscle mass (12, 13), the SkM-TSC2-5A mice demonstrate that abrogation of a single key regulatory input into mTORC1, from feeding, has specific effects on muscle function without altering muscle mass.

The SkM-TSC2-5A model separates feeding-induced mTORC1 activation and postprandial protein synthesis from muscle mass and function. While previous studies implicate AKT-mediated activation of mTORC1 as crucial for muscle mass development (2, 20), the SkM-TSC2-5A model provides genetic evidence that decreased AKT-mediated mTORC1 activation and the subsequent loss of postprandial protein synthesis does not alter skeletal muscle mass. Despite the evidence provided here that mTORC1-mediated protein synthesis in response to refeeding is dispensable for muscle mass, mTORC1 is undeniably required during phases of rapid muscle growth, such as during development and mechanical loading-induced hypertrophy (13, 15, 58, 61). Therefore, mTORC1-dependent maintenance of muscle mass in SkM-TSC2-5A mice may occur through sustained contraction-mediated mTORC1 activation or other alternative signals beyond those derived just after food intake. In support of this hypothesis, SkM-TSC2-5A mice maintain sensitivity to contraction-mediated mTORC1 activation. Furthermore, rates of protein synthesis do not significantly differ between genotypes in the fasted state, indicating that signals outside of feeding-induced mTORC1 maintain skeletal muscle protein synthesis in other contexts. Feeding-induced mTORC1 activation and protein synthesis may instead be critical for skeletal muscle growth through synergizing with other signals. In support of this, feeding in combination with contraction potently increases muscle protein synthesis and hypertrophy relative to feeding alone (5, 68). Nutrient intake is clearly essential for both the maintenance and growth of muscle (3), but the effects of acute refeeding on mTORC1 do not appear essential for either.

While acute feeding activates mTORC1 via AKT, AKT has functions independent of mTORC1 that can contribute to the control of muscle mass (1, 11). The skeletal muscle of SkM-TSC2-5A mice repeatedly displayed hyperphosphorylation of AKT in response to feeding, likely due to the loss of established mTORC1-mediated negative-feedback effects on AKT activation (39–41). Therefore, maintenance of skeletal muscle mass in SkM-TSC2-5A mice may also be supported by enhanced AKT signaling. Skeletal muscle–specific AKT1/AKT2 double-KO mice display a reduction in muscle mass due predominantly to reductions in protein synthesis, with little change detected in muscle proteolysis or atrophy-related gene expression (11). Importantly, protein synthesis was only restored to the skeletal muscle of these mice upon deletion of both forkhead box O1 (FOXO1) and TSC1, suggesting that reactivating mTORC1 alone, albeit constitutively through TSC1 deletion, is not sufficient to restore skeletal muscle protein synthesis in the absence of AKT (11). This suggests that AKT-dependent but TSC- and mTORC1-independent pathways may also regulate protein content. Thus, elevated AKT signaling in SkM-TSC2-5A muscles might also contribute to the maintenance of muscle mass despite reduced mTORC1 activation. This is consistent with the findings that skeletal muscle–specific expression of constitutively active AKT results in massive skeletal muscle hypertrophy, yet chronic hyperactivation of mTORC1 through KO of TSC1, which suppresses AKT activation, results in myopathy (12, 14, 20). Furthermore, the feeding-induced hormone insulin, which activates AKT, has been suggested to regulate muscle mass primarily by suppressing catabolic processes, rather than activating anabolic ones (1, 17, 69). These findings support the notion that elevated AKT activation in SkM-TSC2-5A mice may offset reductions in postprandial protein synthesis through mTORC1-independent pathways and that the acute anabolic effect of feeding to increase protein synthesis may be insufficient for feeding to promote muscle mass.

The clear signaling changes in the skeletal muscle of SkM-TSC2-5A mice did not result in major changes to skeletal muscle contractile function. Despite this, male SkM-TSC2-5A mice demonstrate improved endurance capacity. While genetic ablation of mTORC1 from skeletal muscle results in both increased glycogen content and fiber-type shifts (13), neither glycogen content nor fiber-type proportion were different between muscles from SkM-TSC2-WT and -5A mice. Skeletal muscle ablation of mTORC1 was associated with reduced muscle mitochondrial content (13, 70), while the specific decrease in feeding-induced muscle mTORC1 signaling in the SkM-TSC2-5A mice, instead, was accompanied by a mild gain in mitochondrial content. How mTORC1 signaling positively or negatively regulates skeletal muscle mitochondrial content, directly or indirectly, in these or any setting is currently unknown.

In contrast to SkM-TSC2-5A mice, whole-body TSC2-5A mice generated through use of CMV-Cre, which is expressed across all cell types, have a decrease in lean mass, muscle weights, and myofiber size, accompanying their decrease in feeding-induced muscle mTORC1 signaling and protein synthesis (36). While the smaller muscle size of these mice correlated with reductions in muscle protein synthesis, the phenotypic separation of muscle size and feeding-induced protein synthesis in this current study strongly suggests that postprandial protein synthesis does not determine muscle size in sedentary mice. Therefore, the reduction of muscle size in whole-body TSC2-5A mice likely reflects a role for alternative cell or tissue types in this phenotype, rather than something intrinsic to terminally differentiated skeletal muscle.

In summary, this study shows that acute feeding–induced muscle mTORC1 signaling and protein synthesis are dependent on AKT-mediated phosphorylation of TSC2 in skeletal muscle. However, we provide genetic evidence muscle protein synthesis induced just after feeding does not determine muscle mass or strength in sedentary mice. AKT-mediated regulation of the TSC complex and mTORC1 may have a negative effect on skeletal muscle mitochondrial content through an undetermined mechanism, and the loss of mTORC1 regulation through this signal improves endurance capacity in male mice.

### Limitations of this study

While no changes in heart TSC2 phosphorylation or mass were observed in SkM-TSC2-5A mice, *HSA-Cre* has been found to express in all striated muscle tissues, including the heart (37). Whole-body TSC2-5A mice, produced with CMV-Cre, lack TSC2 phosphorylation in the heart and have reduced heart mass, further supporting that the model described here is skeletal muscle specific (36). Signaling was assessed primarily at 2 hours of refeeding, a time point that might be too long for some acute signaling events and too short for other downstream outcomes, including transcriptional regulation, to be detected (71). While the experimental paradigm used throughout was designed to reproducibly detect acute changes in skeletal muscle signaling and protein synthesis induced specifically by food consumption across a cohort of mice, it does not represent the more physiological ad libitum context in which muscle mass and function are assessed. While the expression level of genes critical for muscle protein degradation were found to be indistinguishable between SkM-TSC2-WT and -5A muscles, proteasome function, autophagic flux, and protein degradation rates were not directly measured, preventing definitive conclusions on the effect of feeding-induced mTORC1 on skeletal muscle protein turnover. Furthermore, while a defect in feeding-induced mTORC1 signaling is seen in both male and female SkM-TSC2-5A mice at 2 and 8 months of age, there are unexplained age- and sex-specific differences in protein synthesis rates and endurance capacity observed, which await further study (72).

## Methods

### Sex as a biological variable.

This study examined male and female animals, with both similar findings and sex differences reported. Rationale for investigating 1 sex upon the occurrence of sex differences is indicated within the text.

### Animals.

Mice were housed 3–4 mice per cage in standard static microisolator top cages at the Harvard T.H. Chan School of Public Health and maintained in a temperature-controlled, pathogen-free facility undergoing 12-hour light:dark cycles with ad libitum access to autoclaved chow (PicoLab Rodent Diet 20: LabDiet, 5053) and water.

*Rosa26^LSL-TSC2-WT^ Tsc2^fl/fl^* and *Rosa26^LSL-TSC2-5A^ Tsc2^fl/fl^* mice were generated as previously described (36, 38). For skeletal muscle–specific transgene expression, these lines were bred to commercially available mice expressing *HSA (ACTA1)-Cre* (The Jackson Laboratory, 006149) (37) to generate SkM-TSC2-WT and -5A colonies. Mice were maintained via heterozygous crosses of *Rosa26^LSL-TSC2-WT/LSL-TSC2-5A^ Tsc2^fl/fl^ HSA-Cre* (SkM-TSC2-WT/5A) mice. F1 homozygous SkM-TSC2-WT and -5A offspring generated from above P1 heterozygous crosses were bred to generate F2 cohorts for all experiments. SkM-TSC2-WT and -5A lines were not bred separately for more than 1 generation to minimize potential of genetic drift.

To genotype mice, DNA was extracted from ear punches or tail snips, the gene regions of interest were amplified by PCR using the following primer sequences (5′-3′): R26: TTGCTCTCCCAAAGTCGCTCTGA, AATCTGTGGGAAGTCTTGTCC, CATCAAGGAAACCCTGGACTACTG; Ser939/981: CGTCATAGCCATGTGGTTCA, ATCATGTCCAGACAGGTTTCC; *Tsc2*: ACAATGGGAGGCACATTACC, AAGCAGCAGGTCTGCAGTG; *Cre*: CGCCGCATAACCAGTGAAAC, ATGTCCAATTTACTGACCG; and *Fabp* (Cre^–/–^ control): TGGACAGGACTGGACCTCTGCTTTCCTAGA, TAGAGCTTTGCCACATCACAGGTCATTCAG.

PCR products were analyzed by agarose gel electrophoresis. Detailed methods for genotyping TSC2-WT and -5A mice were previously described (36).

### Protein extraction and immunoblotting.

Tissues from freshly euthanized animals were flash frozen in liquid nitrogen for subsequent analyses. Whole skeletal and cardiac muscle were lysed in cold Triton Lysis Buffer (40 mM Tris, 5 mM EDTA, 0.5% Triton X-100, 25 mM β-glycerophosphate) with protease (Sigma-Aldrich, P8340) and phosphatase inhibitors (Roche, 4906845001). Tissues were lysed using a handheld homogenizer (Omni International Homogenizer) for 3 × 10-second pulses from 5,000 to 35,000 rpm. Skeletal and cardiac muscle lysates were rotated at 4°C for 2–3 hours to improve solubilization. Lysates were centrifuged at 20,000*g* for 15 minutes at 4°C, and protein concentrations of the supernatant were determined using the Pierce Detergent-Compatible Bradford assay (Thermo Fisher Scientific, 23246). Equal amounts of protein were loaded onto 4%–15% polyacrylamide gradient gels (Bio-Rad, 5678085). Proteins were separated by electrophoresis using Criterion Cell (Bio-Rad, 1656001) and transferred to nitrocellulose membranes using Criterion Blotter (Bio-Rad, 1704070). After transfer, membranes were blocked with 5% nonfat milk in Tris-buffered saline plus Tween-20 (TBS-T; 25 mM Tris, 150 mM NaCl, 2.5 mM KCl, 0.1% Tween-20) for 30 minutes at room temperature (RT). For immunoblotting, membranes were incubated with primary antibodies (1:1,000) in TBS-T with 5% BSA at 4°C overnight. Membranes were washed 3 × 5 minutes with TBS-T then incubated with IRDye 800CW anti-rabbit (LI-COR, 926-32211) or anti-mouse (LI-COR, 926-32210) secondary antibodies at 1:5,000 in 5% nonfat milk for 1 hour at RT. Membranes were washed 3 × 5 minutes in TBS-T and imaged using the LI-COR Odyssey CLx imaging system. LI-COR images were quantified using FIJI (73).

The following primary antibodies from Cell Signaling Technologies were used: p-TSC2 (S939; catalog 3615), p-TSC2 (T1462; catalog 3617), TSC2 (catalog 3612), p-AKT (S473; catalog 4060), AKT (catalog 4691), p-S6 (S240/244; catalog 2215), S6 (catalog 2217), 4E-BP1 (catalog 9644), p-S6K1 (T389, catalog 9205), S6K (catalog 2708), p-TFEB (S122, catalog 87932), p-ULK1 (S757, catalog 6888), ULK1(catalog 4773), pGSK3α/β (S21/9, catalog 9331), and GSK3β (catalog 9332). Anti-TFEB (catalog A303-673) was obtained from Fortis Life Sciences, and anti-puromycin (catalog MABE343MI) from Thermo Fisher Scientific.

### In vivo signaling experiments and protein synthesis assay.

Mice were fed ad libitum, fasted, or refed as indicated. In indicated experiments, mice were treated with or without the AKT inhibitor MK2206 (180 mg/kg bodyweight in 30% Captisol [sulfobutylether-*β*-cyclodextrin]) via oral gavage or the mTORC1 inhibitor rapamycin (1 mg/kg bodyweight) via i.p. injection 30 minutes prior to refeeding. Rapamycin was resuspended in 100% ethanol and diluted 1:20 in resuspension buffer (90% 1×PBS, 5% PEG200, 5% tween80). For measurements of protein synthesis, 30 minutes prior to euthanasia and tissue harvest, mice were injected i.p. with 40 nmol/g bodyweight of puromycin dissolved in PBS as previously described (34, 36, 74).

Protein was extracted and immunoblotted as described above. Total protein was assessed with Revert 700 Total Protein Stain (LI-COR, 926-11011) in the 700 nm channel using LI-COR Odyssey CLx imaging system. Puromycin incorporation was assessed by immunoblotting for puromycin (1:500) in the 800 nm channel, on the same membrane as total protein quantification. Protein synthesis was quantified using FIJI by normalizing puromycin intensity to total protein intensity.

### Gene expression analysis.

Skeletal muscle was lysed in TRIzol (Invitrogen, 15596018) on ice, and RNA was isolated following manufacturer’s instructions. RNA was reverse transcribed using the Advanced cDNA Synthesis Kit (Bio-Rad, 1725038). qPCR analysis was performed with technical triplicates using iTaq SYBR green (Bio-Rad, 172-5124) and the Quant Studio 7 Flex Real-Time PCR System. Samples were normalized to 36B4 (mouse) for ΔΔCt analysis. Primer sequences included the following: *Fbxo32*: TTCTCGACTGCCATCCT, ATGCCACTCAGGGATGT; *Gadd45α*: ATTACGGTCGGCGTGTA, GATGAGGGTGAAATGGATCTG; *Myh1*: CGGAGTCAGGTGAATACTCACG, GAGCATGAGCTAAGGCACTCT; *Myh2*: ACTTTGGCACTACGGGGAAAC, CAGCAGCATTTCGATCAGCTC; *Myh4*: AAACCACCTCAGAGTTGTGGA, GTTCCGAAGGTTCCTGATTGC; *Tnni1*: GATGGGAGATGAGGAGAAGC, GGGCAGTGTTCTGACAGGTA; *Tnni2*: GGCCTCTGAGCACAGAAAGT, GCCGGGAGCTATCTTTCTTA; *Trim63*: GAGAACCTGGAGAAGCAGCT, CCGCGGTTGGTCCAGTAG; *Tsc2*: GCATGCAGTTCTCACCTTAT, GGGTAGTCCTTGATCACTTTG; *TSC2*: GGCTCTATTCTCTCAGGAACTC, GATGGACAGGACGATCTCATAG; and *36B4*: AGATGCAGCAGATCCGCAT, GTTCTTGCCCATCAGCACC.

### Metabolite extraction and LC-MS analysis in skeletal muscle.

Adapted from Hui et al. (75). In brief, whole, flash-frozen quadriceps were pulverized using a Cryomill (Retsch) until forming a fine powder. In total, 20 mg of tissue was resuspended in ice-cold metabolite-extraction buffer (methanol/acetonitrile/water at 40:40:20 volume ratio) at a 25 mg/mL ratio. Samples were vortexed and incubated for 10 minutes on ice, centrifuged at 1,000*g* for 5 minutes at 4**°**C. In total, 500 μL of supernatant was aliquoted into a new tube and centrifuged at 21,000*g* for 10 minutes at 4**°**C. Supernatant was transferred to an HPLC vial, from which 5 μL were injected into the LC-MS.

Chromatographic separation was achieved using Vanquish UHPLC (Thermo Fisher Scientific) and XBridge BEH Amide XP column (2.5 μm, 2.1 mm × 150 mm) with guard column (2.5 μm, 2.1 mm × 5 mm) (Waters). The mobile phase A was water/acetonitrile 95:5, and the mobile phase B was water/acetonitrile 20:80, with both phases containing 10 mM ammonium acetate and 10 mM ammonium hydroxide. The elution linear gradient was: 0–3 minutes, 100% B; 3.2–6.2 minutes, 90% B; 6.5–10.5 minutes, 80% B; 10.7–13.5 minutes, 70% B; 13.7–16 minutes, 45% B; and 16.5–22 minutes, 100% B, with flow rate of 0.3 mL/min. The autosampler was at 4°C, with the injection volume of 5 μL. Between-samples needle wash (methanol/acetonitrile/water at 40:40:20) was used. Samples were analyzed using an Orbitrap Exploris 480 mass spectrometer (Thermo Fisher Scientific) and scanned from 70 to 1000 *m*/*z* with switching polarity. The resolution was 120,000. Metabolites were identified based on accurate mass and retention time using EI-Maven (Elucidata) with our in-house library. Relative metabolite abundance was determined by total ion counts across conditions.

### Indirect calorimetry and behavioral assessments.

Mice were housed individually in cages inside Environmental Control Cabinets (Sable Systems #CAB-16 L_Y), set at 21°C, with lights on from 07:00 to 19:00 hours, and chamber humidity at 43%–63%. The following was measured using the Sable Promethion CORE Metabolic and Behavioral Analysis System: oxygen consumption (VO_2_), carbon dioxide production (VCO_2_), RER, energy expenditure (EE), wheel running, food intake, water intake, bodyweight, and locomotor activity. Mice were acclimated to the cages for 5 days prior to analysis with 1 day of acclimation connected to the system. Mice were assessed for 6 days with 3 days of wheel access followed by 3 days without wheel access. Afterward, mice underwent a 16-hour overnight fast (17:00–09:00) followed by ad libitum access to food to assess substrate utilization and eating habits under the conditions of protein synthesis experiments. Body composition was assessed before and after each study. Data were assessed using the CalR application (76).

### Body composition.

Body composition was measured in awake mice using the EchoMRI analyzer system (EchoMRI-100, EchoMRI LLC). Mice were placed in a ventilated plastic tube, and movement was restricted by the insertion of a secondary cylinder into the enclosed tube. The restricted mouse was then inserted into the EchoMRI to measure lean and fat mass. Immediately following assessment, mice were returned to standard housing.

### Immunostaining of skeletal muscle cross-sections.

Skeletal muscles were dissected and immediately frozen in Tissue-Tek O.C.T. Compound (Sakura, 4583) in isopentane cooled in liquid nitrogen for 10–30 seconds depending on muscle size. Samples were immediately placed on dry ice then transferred to –80°C for storage. Frozen tissues were transferred on dry ice to –20°C cryostat for 1 hour (Microtome HM 505N Cryostat). Muscle samples were cut transversely at the midbelly. Serial sections were collected at 10 μm thickness onto Superfrost microscope slides (VWR; 48311-703) and stored at –80°C until staining.

Slides were dried for 30 minutes at RT. Slides were fixed in 4% paraformaldehyde for 15 minutes at 4°C. Slides were washed 3 times for 5 minutes in 1X PBS. Heat-induced antigen retrieval (HIAR) was performed in 1-L 10mM citrate buffer (0.5 mL Tween 20, pH 7). Slides were placed in warmed citrate buffer and maintained at a low boil for 10 minutes. Citrate buffer was diluted by consistent running of distilled water for 10 minutes. Slides were placed in permeabilization buffer (0.1M glycine, 0.1% Triton-X, syringe filtered in 1X PBS) for 10 minutes, followed by blocking buffer (5% goat serum [Jackson ImmunoResearch; 005-000-121], 2% bovine serum albumin [Sigma-Aldrich; A9647], 1:40 Mouse on Mouse blocking reagent [Vector Laboratories; MKB-2213-1], in 1X PBS, syringe filtered) for 1 hour at RT. Slides were incubated overnight at 4°C with the following: Alexa Fluor 594–conjugated wheat germ agglutinin (WGA, Thermo Fisher; W11262), myosin heavy chain Type I (BA-D5 [IgG2b]; concentrate, Developmental Studies Hybridoma Bank; 1:500), Type IIA (SC-71 [IgG1]; concentrate, Developmental Studies Hybridoma Bank; 1:1000), and Type IIB (BF-F3 [IgM]; concentrate, Developmental Studies Hybridoma Bank; 1:200). Slides were washed 3 times for 5 minutes each in 1X PBS, incubated for 1 hour at RT with Alexa Fluor 568 anti-Mouse IgG2b (Jackson ImmunoResearch; 115-575-207), Alexa Fluor 488 Goat anti-Mouse IgM, μ-chain specific (Jackson ImmunoResearch; 115-545-075), and DyLight 405 anti-Mouse IgG1 (Jackson ImmunoResearch; 115-475-205). Slides were washed 3 times for 5 minutes with 1X PBS, mounted with VectaShield Hardset (Vector Laboratories; H-1400-10). Slides were incubated at RT for 30 minutes and were then sealed with clear nail polish.

Slides were imaged with a Nikon Eclipse Ti microscope using an X-Cite 120LED lamphouse. Images were captured on an ANDOR Zyla 5.5 SCMOS camera using NIS-Elements software. All images were captured at 10x magnification under 20% iris intensity. Images of whole cross-sections were taken with 15% overlap and stitched together using NIS-Elements software. Whole plantaris muscles were analyzed; uniform areas of red gastrocnemius directly adjacent and lateral to the plantaris measuring 2 × 10^6^ ± 4.3 × 10^3^ μm^2^ per section were analyzed.

Brightness of images was uniformly adjusted for analysis. CSA of fiber types were assessed with semiautomated muscle analysis using segmentation of histology (SMASH) in a blinded fashion (77). Fibers were manually outlined as needed, and all Type I fibers were manually outlined due to fluorophore overlap. Images were analyzed with the following parameters, pixel size 0.658 μm/pixel, minimum fiber area 300 μm^2^, maximum fiber area 8000 μm^2^, maximum eccentricity 0.97, and minimum convexity 0.7. Myofibers damaged from processing, overlapping myofibers, incomplete myofibers, and nonmyofiber selections were manually removed from SMASH analysis. Both gastrocnemius and plantaris were censored following sample loss (*n* = 1/group). Individual plantaris muscles with extensive damage were censored from analysis (*n* = 1/group), while intact gastrocnemius areas were still assessed.

PAS staining was performed by the Dana Farber/Harvard Cancer Center Rodent Histopathology Core.

### MIC.

Mice were given carprofen in drinking water (0.067 mg/mL) starting 24 hours prior to experiment. Three-month-old male mice were fasted for 2.5 hours prior to procedure. Mice were anesthetized using 4% isoflurane and maintained with ~1% isoflurane via nosecone. Lidocaine (2 mg/kg bodyweight) was injected at the site of the incision. A small incision and blunt dissection tools were used to identify the sciatic nerve. Electrodes were placed transversely across the sciatic nerve and stimulated at 100 Hz with 0.5 ms pulses at 5V (Aurora Scientific, 1300A 3-in-1: Whole-Animal System) for a total of 10 sets of 6 contractions (58). Between each pulse, the leg was manually returned to the starting position by the experimenter. The wound was sealed, and mice were returned to cages without food. Mice were sacrificed 3 hours after final stimulation. Thirty minutes prior to sacrifice, mice were injected with 40 nmol/g bodyweight puromycin.

### Functional strength assessments in mice.

Inverted screen test and grip strength were measured as previously described (62, 78). Unacclimated mice were placed in the center of a wire screen that was inverted to suspend the mice approximately 35 cm above soft bedding. Time from inversion to fall was recorded. Grip strength was measured using a wire rack force meter (Chatillon, DFE-002), according to the manufacturer’s instructions.

### In vivo force and fatigue measurement.

3-month-old male mice were anesthetized using 4% isoflurane and maintained with ~1% isoflurane via nosecone. Mice were placed supine on heat-support while platform was adjusted so the tibia was perpendicular to the foot plate with the leg extended and foot in a relaxed position (Aurora Scientific, 1300A 3-in-1: Whole-Animal System). The foot was secured in the mouse foot plate with surgical tape, and the leg was secured at the knee. For stimulation of the plantarflexors, electrodes were inserted transversely across the proximal region of the peroneal nerve. Isometric contractions were induced via biphasic stimulation first at 50 Hz, then 100 Hz, and 150 Hz as indicated for a 1-second tetanus with 1-minute rest between stimulations. After another minute of rest, fatigue was measured by 50 Hz stimulations for 1-second tetanus and 1-second rest for 150 contractions. Force was recorded for each contraction using accompanying software.

### Treadmill running.

Maximal treadmill running was adapted from a previous study (62). In brief, mice were acclimated for 3 days at 5 m/min for 10 minutes at a 0° incline (Columbus Instruments, Exer-3/6). On the fourth day, mice ran at a 5° incline at 10 m/min for 10 minutes, followed by an increase of 2 m/min every 5 minutes until exhaustion. On the day of experiment, experimenter was blinded to genotype during each run. Exhaustion was defined by the inability to keep hindlimbs off the electric grid for > 5 seconds, at which point, the experiment was terminated, and animals were returned to their cages.

### Glycogen assessment.

Skeletal muscle glycogen was measured using the Glycogen-Glo assay (Promega, catalog J5051) according to manufacturer’s instructions with the following adaptations. Approximately 15 mg of pulverized skeletal muscle was weighed and lysed in HCl supplemented with 0.2% Triton X-100 resulting in samples of equal concentration of tissue and allowed to rotate at 4°C for 2 hours to ensure full digestion. Samples were centrifuged at 4,500*g* for 5 minutes, and supernatant was diluted 1:5. Liver tissue samples and kit-provided standards were used as positive controls.

### GTT and ITT.

Mice were fasted for 6 hours during the early light phase (08:00–14:00) with ad libitum access to water. For GTT, mice were subjected to i.p. injection with glucose in sterile saline (2 g/kg bodyweight). For ITT, mice were subjected to intraperitoneal injection with insulin in sterile saline (0.5 U/kg bodyweight in 0.1% protease-free BSA [HumulinR, Eli Lily]). Blood glucose was monitored periodically as noted using Contour Next EZ gluocometer (Bayer).

### Mitochondrial content.

Mitochondrial content was assessed as previously described (67). Flash frozen quadriceps were pulverized using a Cryomill (Retsch) until forming a fine powder. mtDNA and nDNA was isolated using DNeasy Blood & Tissue Kit (Qiagen, catalog 69504) following manufacturer’s instructions using ~25 mg of tissue. Samples were normalized by total DNA content; then, copy number was assessed using qPCR as described above using the following primers (5′-3′): *16S*: CCGCAAGGGAAAGATGAAAGAC, TCGTTTGGTTTCGGGGTTTC; *HK2*: GCCAGCCTCTCCTGATTTTAGTGT, GGGAACACAAAAGACCTCTTCTGG; and *ND1*: CTAGCAGAAACAAACCGGGC, CCGGCTGCGTATTCTACGTT.

### Statistics.

Statistical analysis was performed using Prism 11.0.1 software. For comparisons between 2 groups, a Mann-Whitney *U* test was performed. For comparisons between slopes, a 2-tailed *t* test was performed. For comparisons of 3 or more independent groups, an ordinary 1-way ANOVA with Tukey’s multiple comparisons was performed. For comparisons with 2 independent factors, a 2-way ANOVA with Holm-Šídák multiple comparisons was performed. For multiple independent comparisons between groups, multiple Mann-Whitney *U* tests with a Holm-Šídák correction were performed. All paired tests were performed 2 sided. Samples were censored in the following experiments: Figure 1F (*n* = 1) and Figure 3D (*n* = 2), when puromycin labeling was not detected indicating a technical failure in puromycin administration. Subject was censored from body composition due to premature death (Figure 5C and Supplemental Figure 5, C and E; *n* = 1). Subjects were censored from inverted screen test for climbing around and above the screen (Figure 8, E and F; *n* = 3). Subjects were censored from fatigue measures due to electrode displacement during experiment (Figure 8G and Supplemental Figure 6E; *n* = 1). *P* < 0.05 was considered significant. *P* > 0.05 and below 0.5 are reported. *P* values above 0.5 are not reported.

### Study approval.

All animal procedures were approved by the Harvard Medical Area Standing Committee on Animal Care and Use (IACUC), an AAALAC International and USDA-accredited facility.

### Data availability.

Values for all data points in graphs are reported in the Supporting Data Values file.

## Author contributions

SCL, YC, KCK, and BDM conceptualized the project. WD and SH performed metabolomics and consulted in analysis. KEI assisted with indirect calorimetry experiments and analysis. YC, KCK, MYC, KT, DMR, and CEM assisted with in vivo experiments. SCL performed all other experiments and analysis. SCL wrote this article with editing from KCK, YC, and BDM. BDM acquired funding. BDM and YC supervised this work. All authors provided critical project insights through group discussions.

## Conflict of interest

BDM is a consultant for GondolaBio and Tarsus on topics unrelated to this article.

## Funding support

This work is the result of NIH funding, in whole or in part, and is subject to the NIH Public Access Policy. Through acceptance of this federal funding, the NIH has been given a right to make the work publicly available in PubMed Central.

- NIH grants T32-DK128781 (SCL and KCK), F31-DK128873 (KCK), R35-CA197459 (BDM), and P01-CA120964 (BDM).
- Glenn Foundation for Medical Research Postdoctoral Fellowship (YC).
- Damon Runyon Cancer Research Foundation Merck Fellowship DRG-#2443-21 (MYC).

## Supplementary Material

Supplemental data

Unedited blot and gel images

Supporting data values

## Figures and Tables

**Figure 1:**
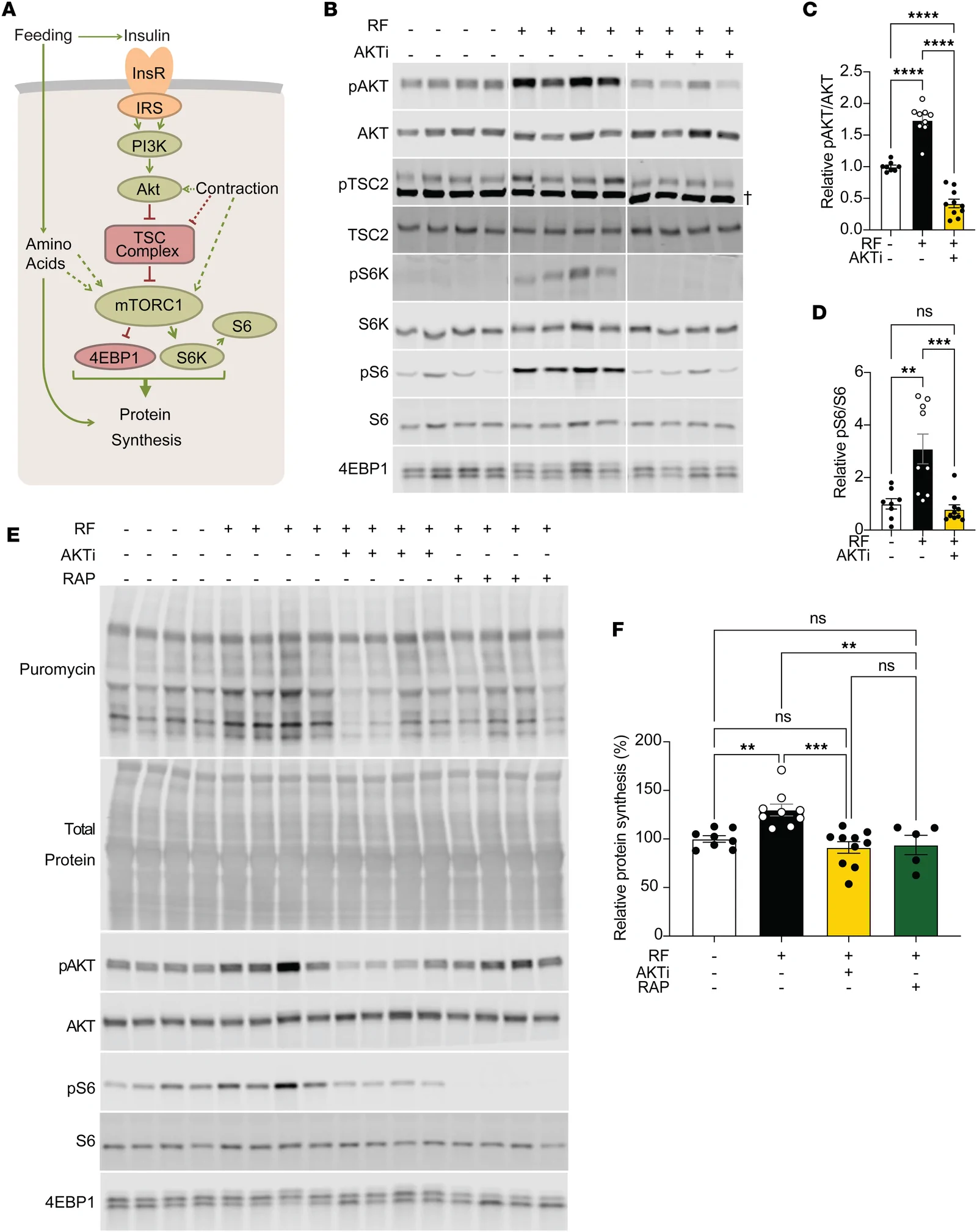
Feeding induces postprandial protein synthesis through activation of AKT and mTORC1. (**A**) Model of the multiple inputs to mTORC1 activation in skeletal muscle and select downstream targets. (**B**–**E**) Representative immunoblots (*n* = 2) and quantification of protein extracts from quadriceps muscle of 4-month-old male C57BL6/J mice after a 16-hour overnight fast with or without a 2-hour refeeding (RF) (*n* = 8–10) with or without MK2206 (180mg/kg bodyweight [AKTi]) pretreatment 30 minutes prior to RF; samples from the same blot and exposure. (**C** and **D**) Quantification of immunoblot data from (**B** and **E**), with relative phospho/total protein graphed as mean ± SEM. (**E** and **F**) Representative immunoblots (*n* = 2) and quantification of protein extracts from quadriceps muscle of 4-month-old male C57BL6/J mice after a 16-hour overnight fast with or without 2-hour RF (*n* = 5–10) with or without AKTi as above or with or without rapamycin (1 mg/kg bodyweight [RAP]) pretreatment 30 minutes prior to RF. Puromycin injected (40 nmol/g bodyweight) 30 minutes prior to sacrifice. (**F**) Quantification of immunoblot data from (**B** and **E**), with relative phospho/total protein graphed as mean ± SEM. (**C**, **D**, and **F**) Ordinary 2-way ANOVA with Tukey’s multiple comparison; **P < 0.01, ***P < 0.001, and ****P < 0.0001. ^†^nonspecific band.

**Figure 2:**
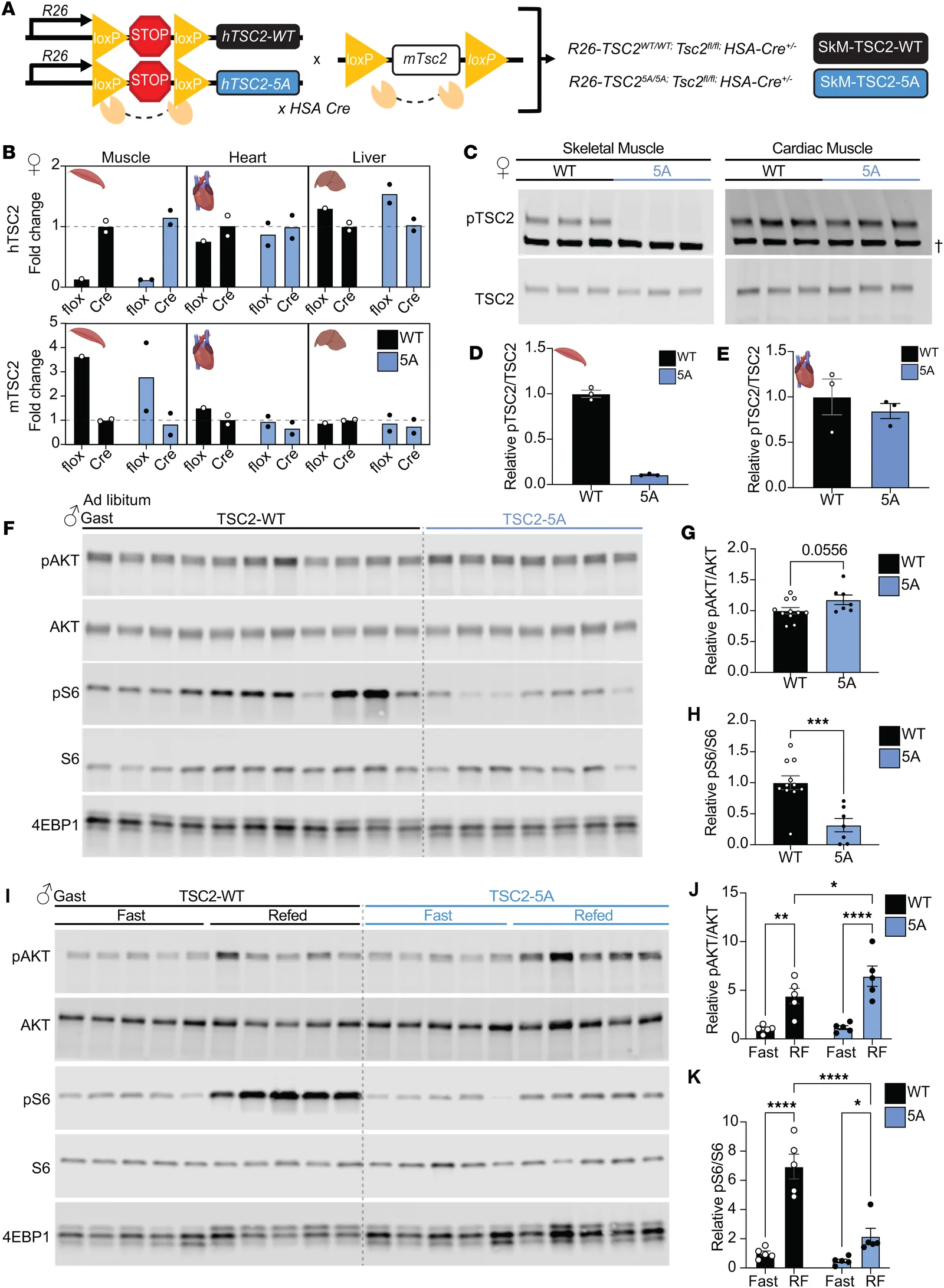
AKT-mediated phosphorylation of TSC2 is required for feeding-induced activation of mTORC1. (**A**) Schematic of conditional mouse model using the *Rosa26 (R26)-lox-STOP-lox-hTSC2-WT* or *-5A* transgene knock-ins and the *Tsc2^fl/fl^* conditional allele with *HSA-Cr*e for generation of SkM-TSC2-WT and -5A mice. (**B**) Ad libitum*-*fed 4-month-old female *R26-lox-STOP-lox-hTSC2-WT* or *-5A; Tsc2^fl/fl^* with or without *HSA-Cre* expression, as indicated. Transcript levels of the human (h)*TSC2* transgene (top) and endogenous mouse (m)*Tsc2* (bottom) in tibialis anterior muscle, cardiac muscle, and liver (left to right), graphed relative to *WT Cre^+^* of the same tissue displayed (*n* = 1–2/group). (**C**–**E**) Immunoblots and quantifications of protein extracts from gastrocnemius and cardiac muscles of 2-month-old female SkM-TSC2-WT and -5A mice RF for 2 hours after a 16-hour overnight fast (*n* = 3/group). (**D** and **E**) Quantification of immunoblot data from **C**, with relative phospho/total protein graphed as mean ± SEM. (**F**–**H**) Immunoblots and quantification of protein extracts from gastrocnemius of 4-month-old male SkM-TSC2-WT and -5A mice fed ad libitum (*n* = 7–11). (**G** and **H**) Quantification of immunoblot data from **F**, with relative phospho/total protein graphed as mean ± SEM. (**I**–**K**) Immunoblots and quantification of protein extracts from gastrocnemius of 3-month-old male SkM-TSC2-WT and -5A mice following a 12-hour daytime fast with or without 2-hour RF, as indicated (*n* = 5/group). (**J** and **K**) Quantification of immunoblot data from **I**, with relative phospho/total protein graphed as mean ± SEM. (**G** and **H**) Mann-Whitney, (**J** and **K**) 2-way ANOVA; *P < 0.05, **P < 0.01, ***P < 0.001, and ****P < 0.0001. ^†^nonspecific band. Created in BioRender. Lapp, S. (2026) https://BioRender.com/sun06xm

**Figure 3:**
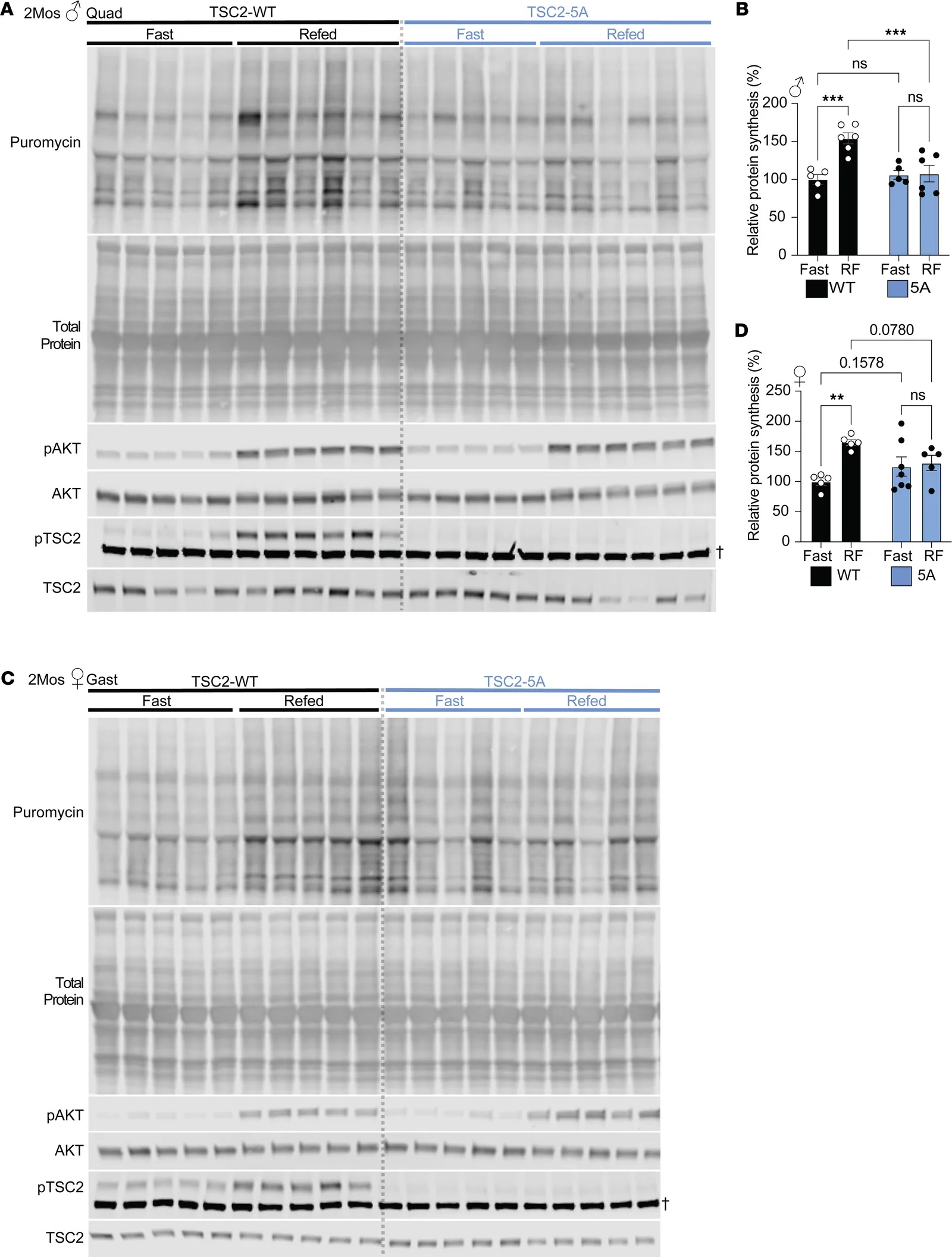
AKT-mediated phosphorylation of TSC2 is required for postprandial protein synthesis in skeletal muscle. (**A** and **B**) Immunoblots and quantification of protein extracts from quadriceps of 2-month-old male SkM-TSC2-WT and -5A mice after a 16-hour overnight fast with or without 2-hour RF (*n* = 5–6/group). Puromycin injected (40 nmol/g bodyweight) 30 minutes prior to sacrifice. (**B**) Quantification of immunoblot data from **A**, with relative puromycin labeled protein/total protein graphed as mean ± SEM. (**C** and **D**) Representative immunoblots and quantification of protein extracts from gastrocnemius of 2-month-old female SkM-TSC2-WT and -5A mice after a 16-hour overnight fast with or without 2-hour RF (*n* = 5–7/group). Puromycin injected (40 nmol/g bodyweight) 30 minutes prior to sacrifice. (**D**) Quantification of immunoblot data from **C**, with relative puromycin labeled protein/total protein graphed as mean ± SEM. (**B** and **D**) 2-way ANOVA. **P < 0.01, ***P < 0.001. ^†^nonspecific band.

**Figure 4:**
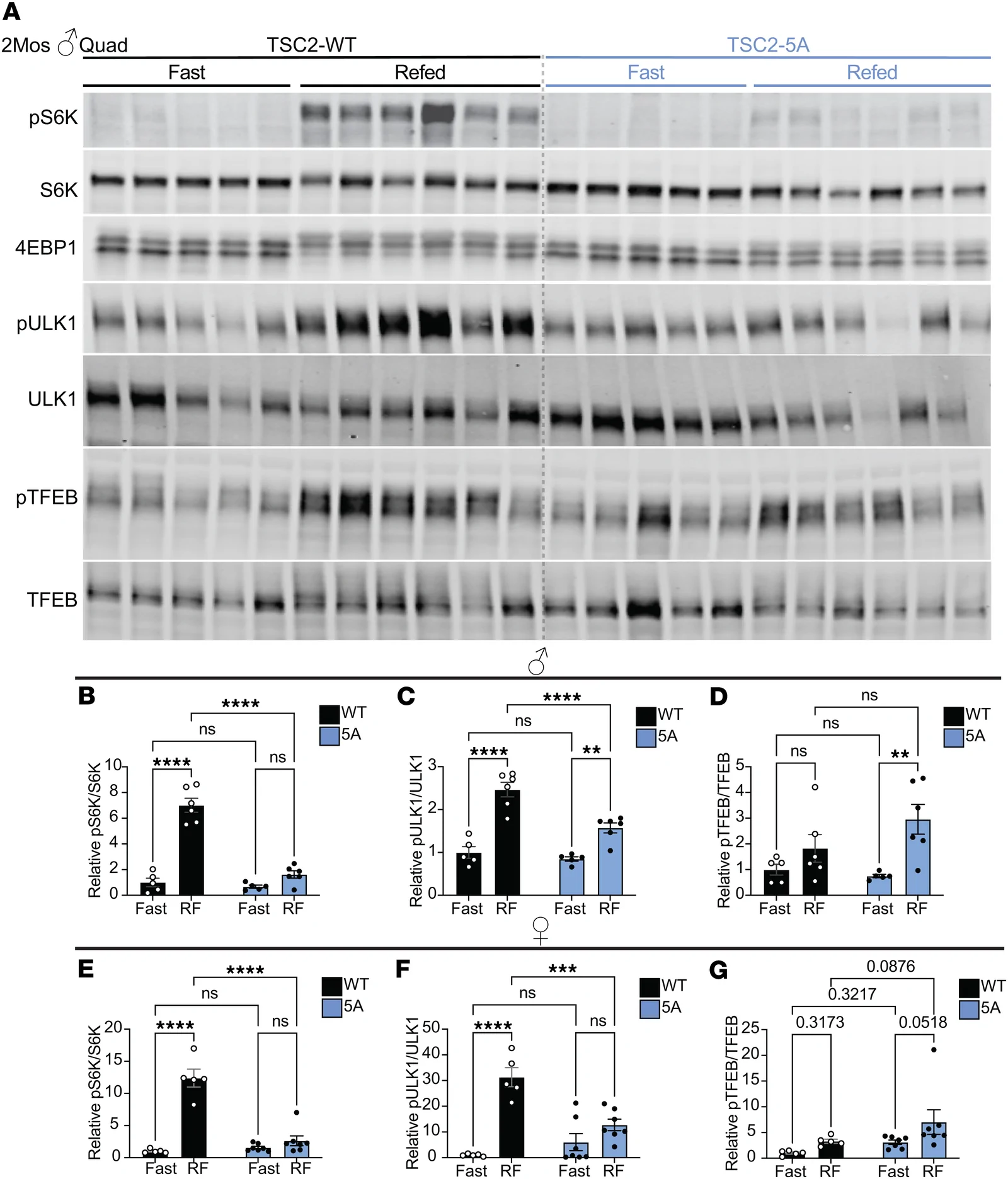
AKT-mediated phosphorylation of TSC2 has substrate specific effects downstream of skeletal muscle mTORC1 signaling. (**A**–**D**) Immunoblots and quantification of protein extracts from quadriceps of male 2-month-old mice after a 16-hour overnight fast with or without 2-hour RF (*n* = 5–6/group). (**B**–**D**) Quantification of immunoblot data from **A**, with relative phospho/total protein graphed as mean ± SEM. (**E**–**G**) Quantification of immunoblots in Supplemental Figure 4A of protein extracts from gastrocnemius of female 2-month-old mice after a 16-hour overnight fast with or without 2-hour RF (n = 5–7/group), with relative phospho/total protein graphed as mean ± SEM. (**B**–**G**) 2-way ANOVA. **P < 0.01, ***P < 0.001, and ****P < 0.0001.

**Figure 5:**
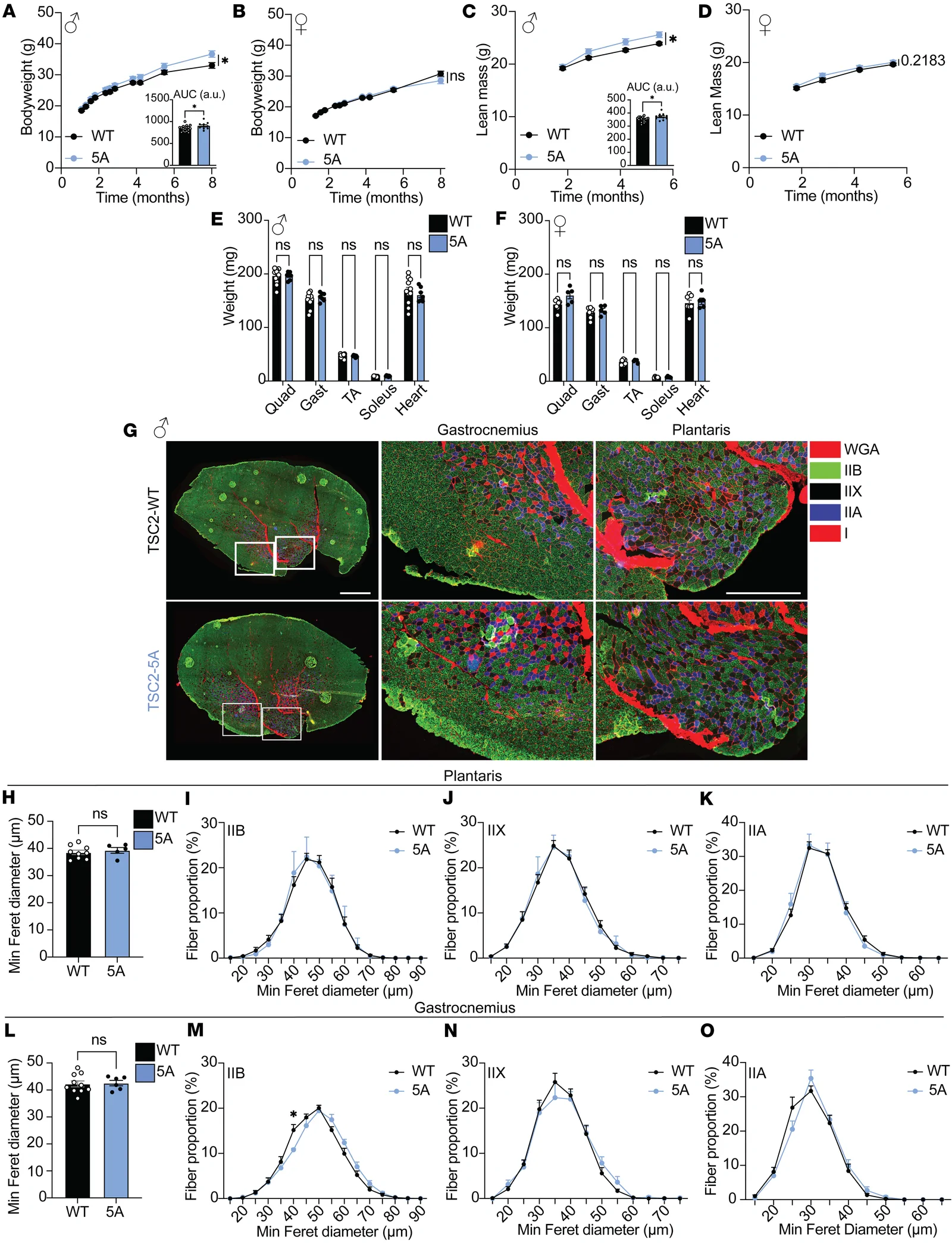
Loss of postprandial mTORC1 activation and protein synthesis does not decrease muscle size. (**A** and **B**) Bodyweight over 8 months graphed as mean ± SEM in male (*n* = 11–19) (**A**) and female mice (*n* = 7–13) (**B**). (**C** and **D**) Lean mass over 6 months graphed as mean ± SEM in male (*n* = 11–18) (**C**) and female mice (*n* = 7–13) (**D**). (**E** and **F**) Muscle weights for quadriceps (Quad), gastrocnemius + plantaris (Gast), tibialis anterior (TA), soleus, and heart in 4-month-old male (*n* = 7–11) (**E**) and female (*n* = 5–7) (**F**) mice. (**G**) Representative immunofluorescence images of male 4-month-old gastrocnemius and plantaris muscles as indicated. WGA (red), Type IIB (green), Type IIX (unstained), Type IIA (blue), and Type I (red). Insets are representative, with whole plantaris and a uniform area of red gastrocnemius (area analyzed: 2 × 10^6^ ± 4.3 × 10^3^ μm^2^) selected for subsequent analyses. Scale bar: 1,000 μm (left); 500 μm (right). (**H**–**O**) Myofiber minimum Feret diameter for (**H**–**K**) plantaris: (**H**) All fibers, (**I**) Type IIB, (**J**) Type IIX, and (**K**) Type IIA (**L**–**O**) gastrocnemius: (**L**) All fibers, (**M**) Type IIB, (**N**) Type IIX (**O**) Type IIA. All data expressed as mean ± SEM. (**A**, **B**, and **D**) Mixed effects analysis; (**C**, **I**–**K**, and **M**–**O**) 2-way ANOVA; (**E** and **F**) Multiple Mann-Whitney *U* with Holm-Šídák correction; (**H** and **L**) Mann-Whitney; *P < 0.05.

**Figure 6:**
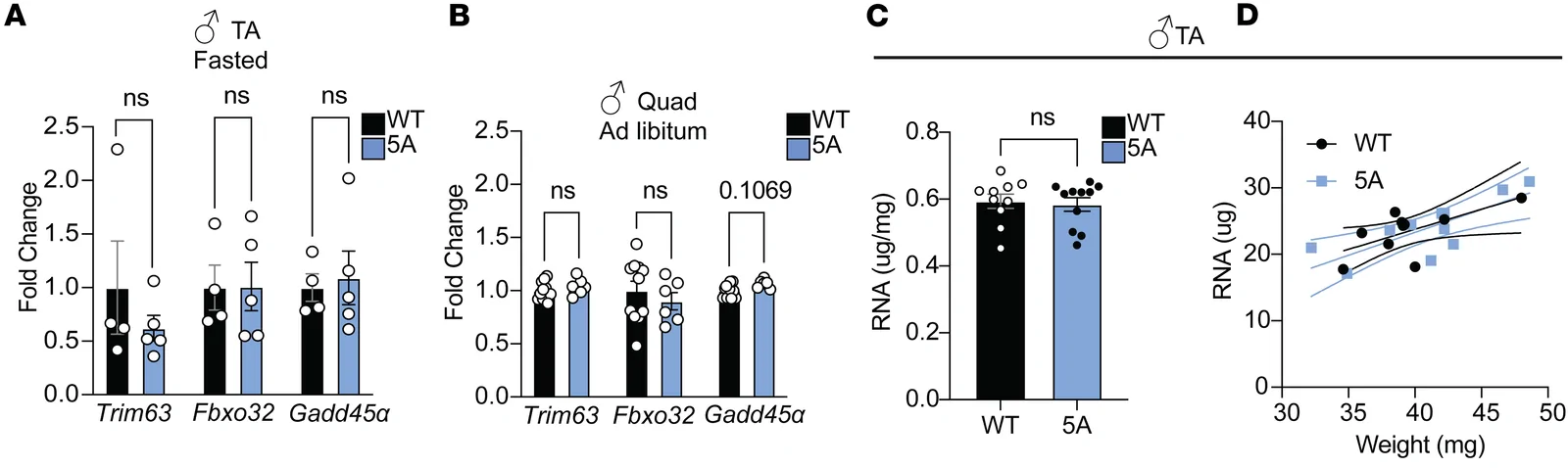
AKT-mediated phosphorylation of TSC2 does not affect skeletal muscle expression of atrophy-related genes or RNA content. (**A** and **B**) Transcript levels of atrophy-related genes (*Trim63*, *Fbxo32*, and *Gadd45a*) in male mice graphed as mean ± SEM relative to the respective -WT in tibialis anterior muscle of male 2-month-old 16-hour fasted mice (*n* = 4,5) and quadriceps of ad libitum fed male 4-month-old mice (*n* = 6–11). (**C** and **D**) RNA content of tibialis anterior muscle from male 2-month-old mice pooled from fasted (n = 4[WT], 5[5A]) and RF (n = 6/group) graphed as (**C**) mean ± SEM (n = 10-11) concentration and (**D**) RNA content as a function of tissue weight (slope-intercept formula [Y=mX+b], coefficient of determination: WT: Y=0.60*X–0.366, R2 = 0.42; and 5A: Y = 0.69X–4.294, R2 = 0.60). (**A** and **B**) Multiple Mann-Whitney *U* tests with Holm-Šídák correction; (**C**) Mann-Whitney *U* test; (**D**) For comparison between slopes, a 2 tailed *t* test was performed.

**Figure 7:**
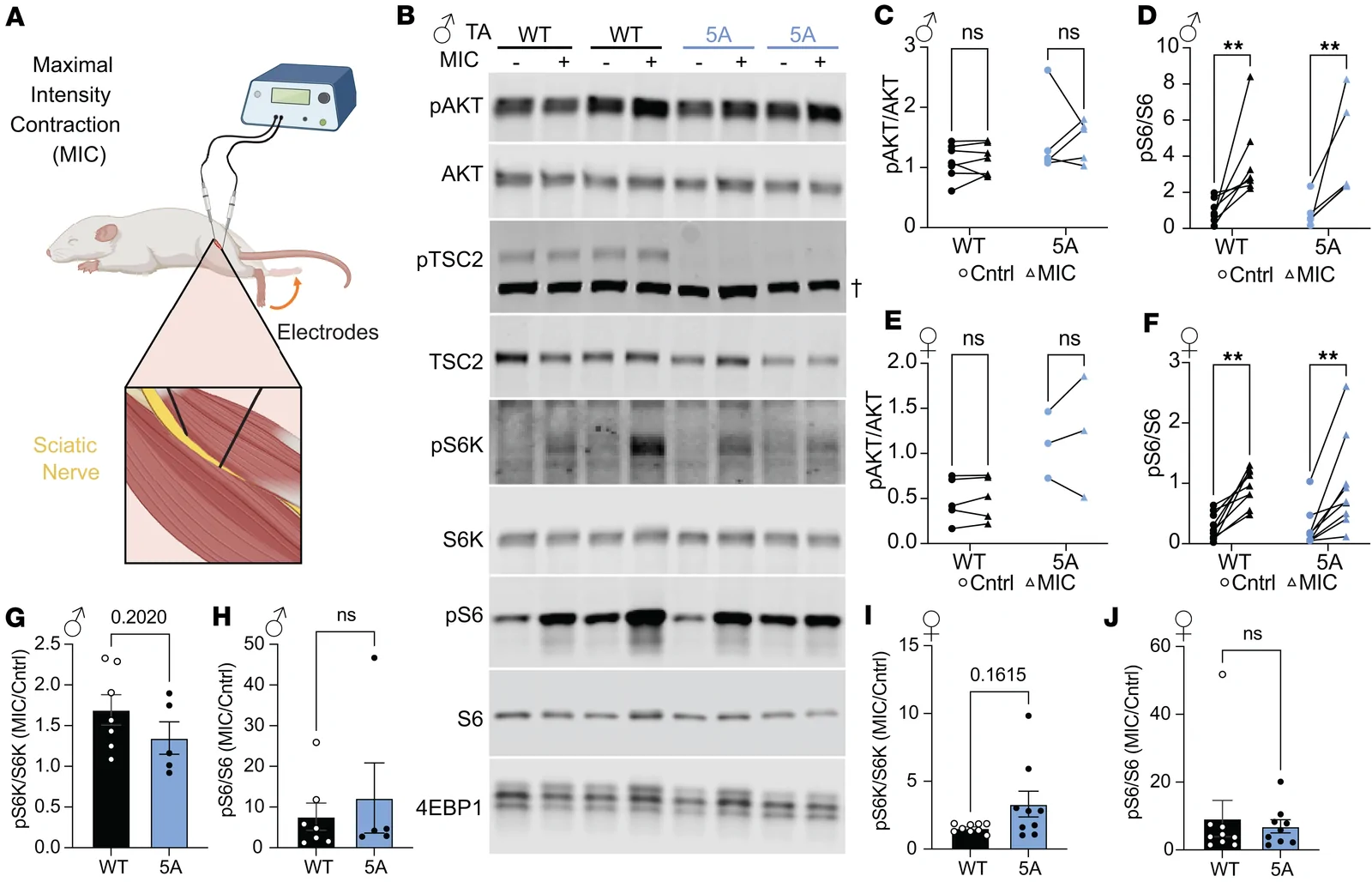
AKT-mediated phosphorylation of TSC2 is dispensable for contraction-mediated mTORC1 activation in skeletal muscle. (**A**) Schematic of direct sciatic nerve stimulation for maximal intensity contraction (MIC) protocol. (**B**–**D**) Representative immunoblots (*n* = 3) and quantification of protein extracts from tibialis anterior of 3-month-old male mice (n = 5[WT], 7[5A]) 3 hours following MIC treatment. Each “WT” or “5A” represents one animal with “–” indicating unstimulated contralateral muscle and “+” indicating the stimulated muscle. (**C** and **D**) Quantification of immunoblot data from **B** and Supplemental Figure 6A, with relative phospho/total protein graphed as change between unstimulated and stimulated muscles from the same mouse. (**E** and **F**) Quantification of immunoblot data from Supplemental Figure 6B, with relative phospho/total protein graphed as change between unstimulated and stimulated muscles from the same mouse from *n* = 1 (**E**) and *n* = 2 independent experiments (**F**). (**G** and **H**) Quantification of fold induction of phospho/total protein from **B** and Supplemental Figure 6A graphed as mean ± SEM. (**I** and **J**) Quantification of fold induction of phospho/total protein from (Supplemental Figure 6B) graphed as mean ± SEM. (**C**–**F**) 2-way ANOVA; (**G**–**J**) Mann-Whitney *U* test. **P < 0.01. ^†^nonspecific band. Created in BioRender. Lapp, S. (2026) https://BioRender.com/kafewsi

**Figure 8:**
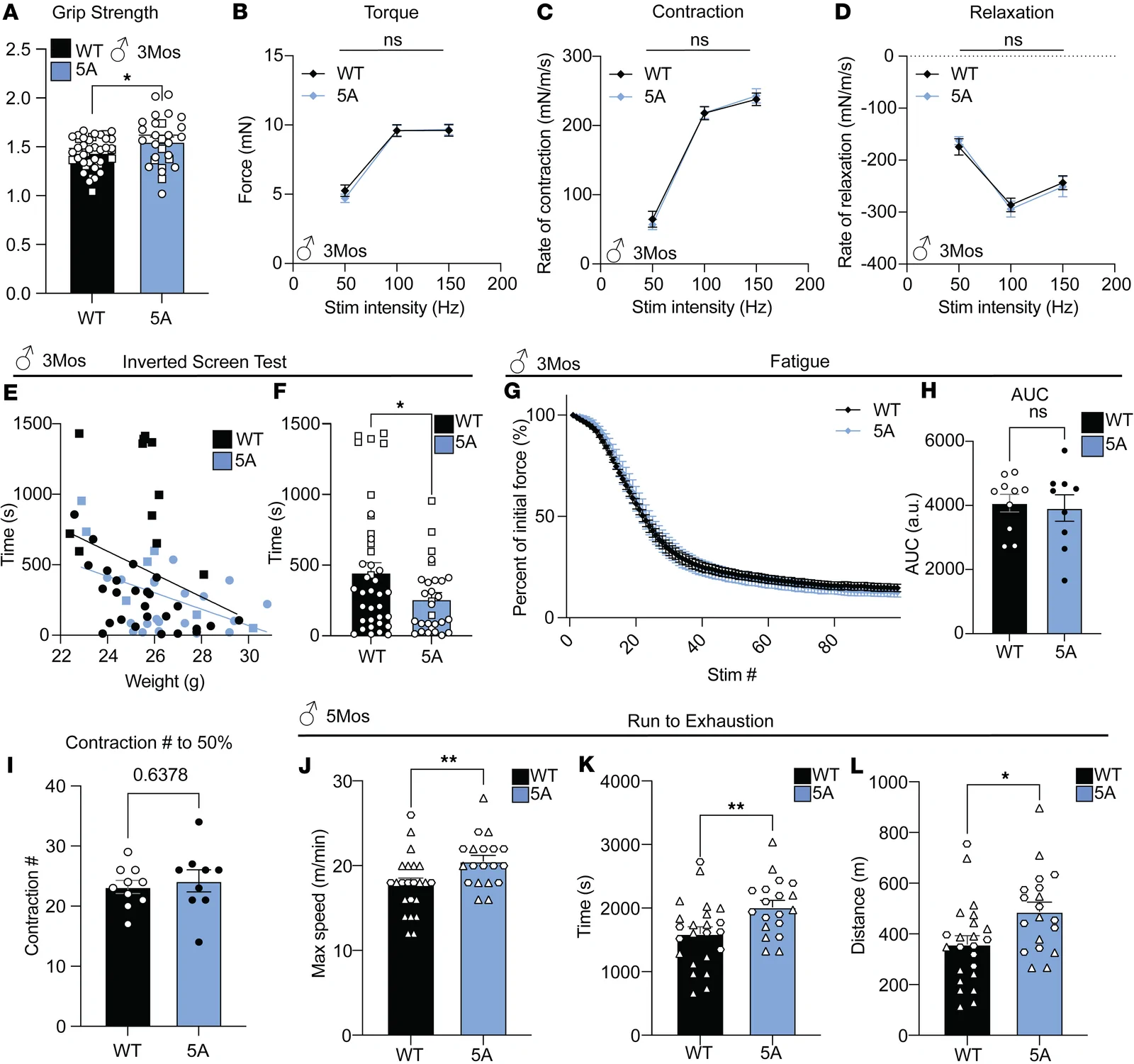
Loss of AKT-mediated phosphorylation of TSC2 enhances maximal endurance capacity without altering contractile function. (**A**) Four-limb grip strength of male 3-month-old mice represented as mean ± SEM (*n* = 28–40) (**B**–**D**) Electrical stimulation of tibial nerve in vivo for functional assessment of plantarflexors in 3-month-old male mice at indicated stimulation intensities represented as mean ± SEM (*n* = 10/group): (**B**) isometric torque, (**C**) rate of contraction, and (**D**) rate of relaxation. (**E** and **F**) Inverted screen test of male 3-month-old mice (*n* = 28–37). (**E**) hang time as a function of bodyweight; (slope-intercept formula [Y=mX+b], coefficient of determination: WT: Y = –80.86*X + 2534, R2 = 0.1; and 5A: Y = –58.18*X + 1811, R2 = 0.2) and (**F**) mean ± SEM. (**G**–**I**) Male 3-month-old mice treated with repeated submaximal 50 Hz stimulations to measure fatigue resistance represented as (**G**) a percentage of initial torque at each stimulation, (**H**) area under the curve (AUC), and (**I**) number of contractions to reach 50% of initial torque represented each as mean ± SEM. (**J**–**L**) Male 5-month-old mice run to exhaustion assessed via graded exercise stress testing assay represented as mean ± SEM (*n* = 19–23): (**J**) maximal speed reached, (**K**) time to exhaustion, and (**L**) total distance run. For data compiled from independent experiments, individual experimental cohorts are identified by use of the same symbols. (**A**, **F**, and **H**–**L**) Mann-Whitney *U* test, (**B**–**D**) 2-way ANOVA, (**E**) Slope *t* test. *P < 0.05, **P < 0.01.

**Figure 9:**
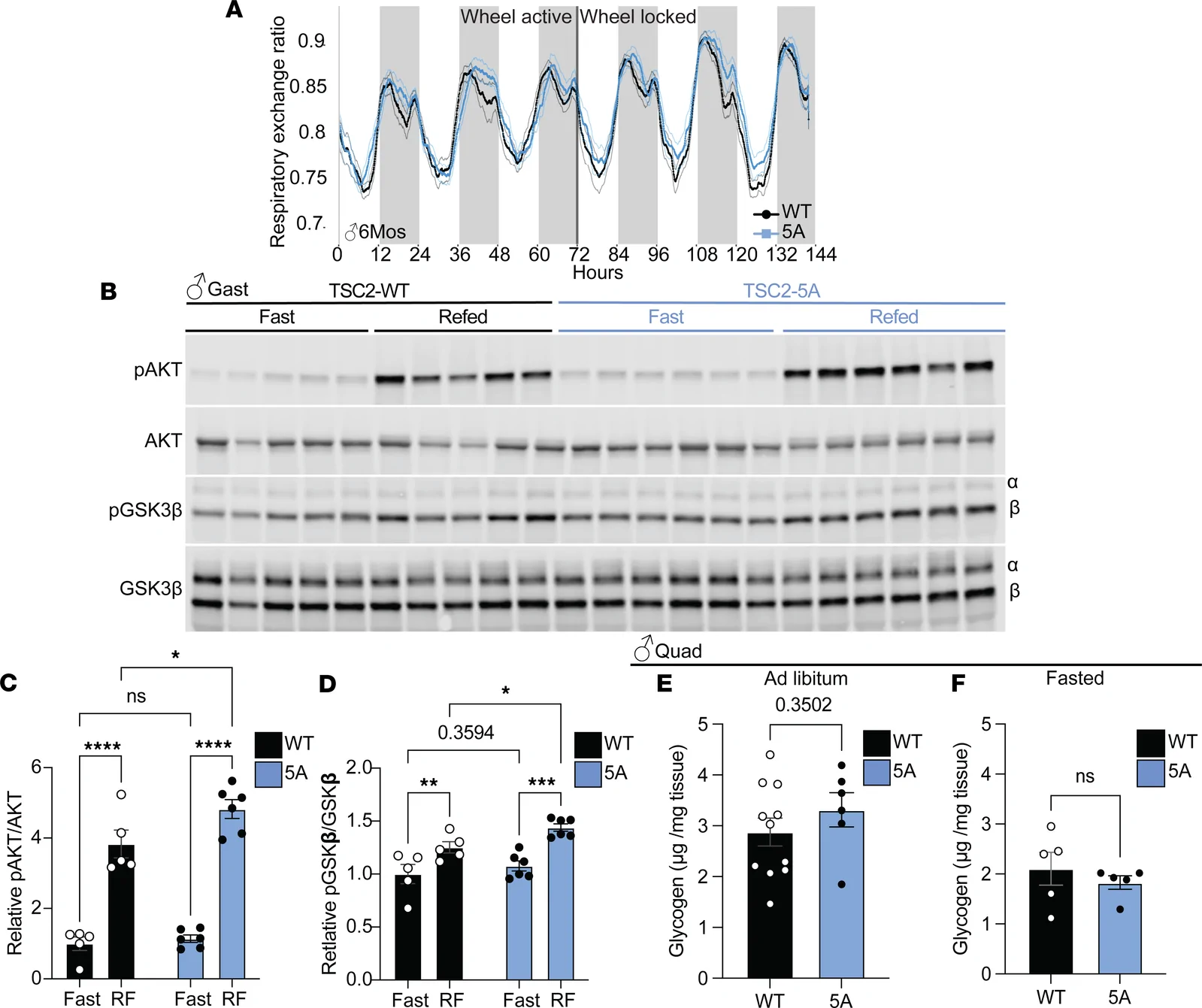
Improved endurance capacity in SkM-TSC2-5A mice is not associated with changes in skeletal muscle glycogen content. (**A**) Respiratory exchange ratio of male 6-month-old mice housed in metabolic cages with free wheel access (0–72 hours) or without free wheel access (73–144 hours) as indicated (*n* = 4/group). (**B**–**D**) Immunoblots and quantification of protein extracts from gastrocnemius of male 2-month-old SkM-TSC2-WT and -5A mice after a 16-hour overnight fast with or without 2-hour RF (*n* = 5–6/group). (**C** and **D**) Quantification of immunoblot data from **B**, with relative phospho/total protein graphed as mean ± SEM. (**E** and **F**) Glycogen content normalized to skeletal muscle weight extracted from quadriceps of male 4-month-old SkM-TSC2-WT and -5A ad libitum fed mice (*n* = 6–11) and quadriceps of male 2-month-old SkM-TSC2-WT and -5A 12-hour fasted mice (*n* = 5/group) as mean ± SEM. (**C** and **D**) 2-way ANOVA; (**E** and **F**) Mann-Whitney. *P < 0.05, **P < 0.01, ***P < 0.001, and ****P < 0.0001.

**Figure 10:**
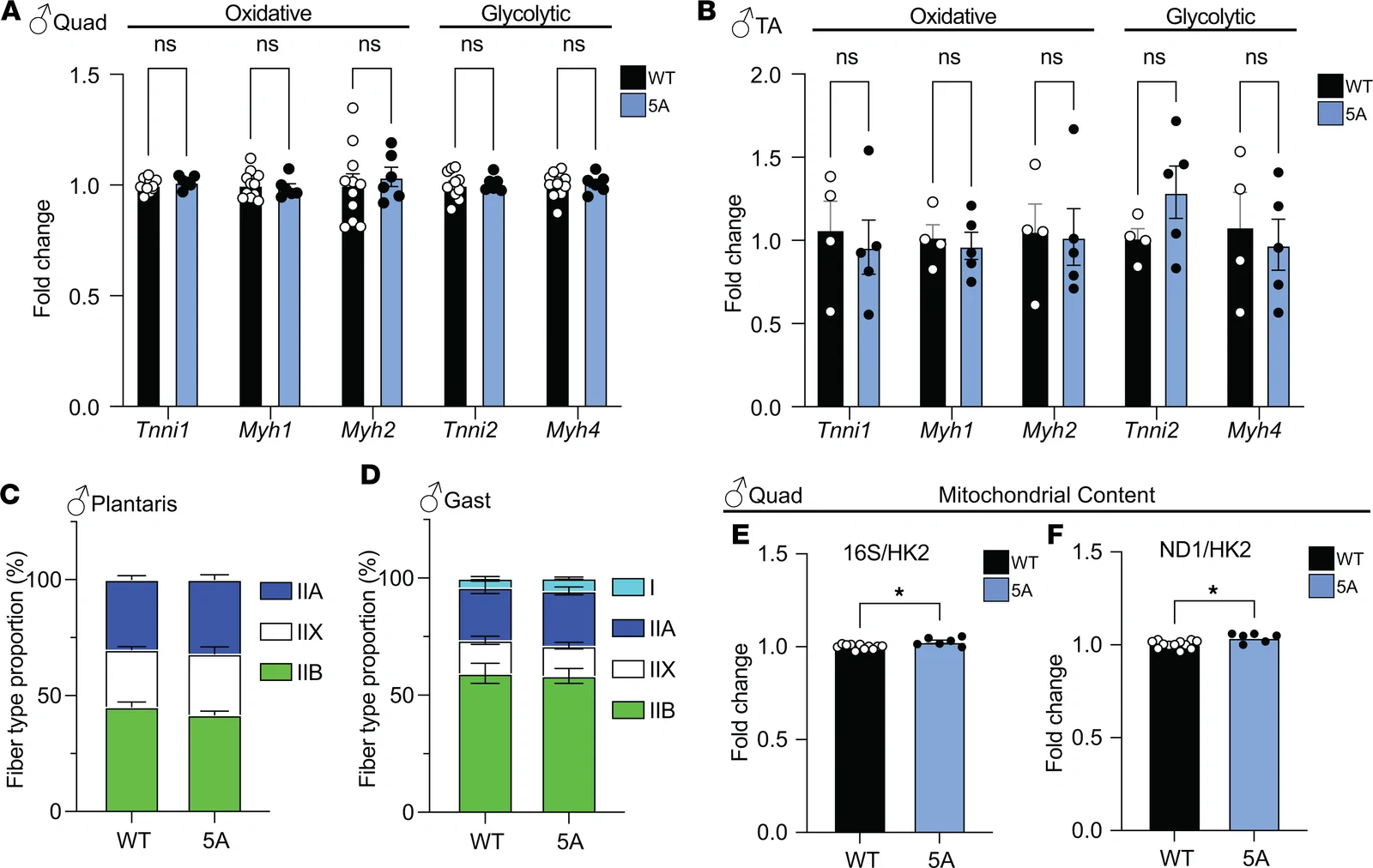
SkM-TSC2-5A muscle displays a moderate increase in mitochondrial content without changes in muscle fiber type. (**A** and **B**) Skeletal muscle fiber type composition as assessed by gene expression of oxidative (*Tnni1*, *Myh1*, *Myh2*) and glycolytic (*Tnni2*, *Myh4*) genes in (**A**) male 4-month-old SkM-TSC2-WT and -5A mice quadriceps (*n* = 6–11) displayed as mean ± SEM relative to WT and (**B**) male 3-month-old SkM-TSC2-WT and -5A quadriceps following 16 hours fasting (*n* = 5/group) expressed as mean ± SEM relative to -WT. (**C** and **D**) Quantification of fiber type proportions in male 4-month-old SkM-TSC2-WT and -5A muscles stained via immunofluorescence in (Figure 5G) for (**C**) the plantaris (*n* = 5–9) and (**D**) gastrocnemius muscles (*n* = 6–10) displayed as mean ± SEM. (**E** and **F**) Mitochondrial DNA copy number normalized to nuclear DNA copy number (*HK2*) in male 4-month-old SkM-TSC2-WT and -5A mouse quadriceps (*n* = 6–11) with either (**E**) *16S* or (**F**) *ND1* as mitochondrial DNA markers. Data displayed as mean ± SEM relative to WT. (**A**–**C**) Multiple Mann-Whitney with Holm-Šídák correction (**E** and **F**) Mann-Whitney *U* test. *P < 0.05, **P < 0.01.

## Version 1 09/10/2026

In-Press Preview

## Version 2 10/22/2026

Electronic publication
