# Supplementary material for: Feeding-induced muscle mTORC1 signaling regulates postprandial protein synthesis and endurance but not muscle size
Source: JCI Insight. 2026 Sep 10;11(20):e210523. doi: 10.1172/jci.insight.210523 (PMC13726562; doi:10.1172/jci.insight.210523)
Supplement: Supplementary file 1 [file jciinsight-11-210523-s170.pdf]

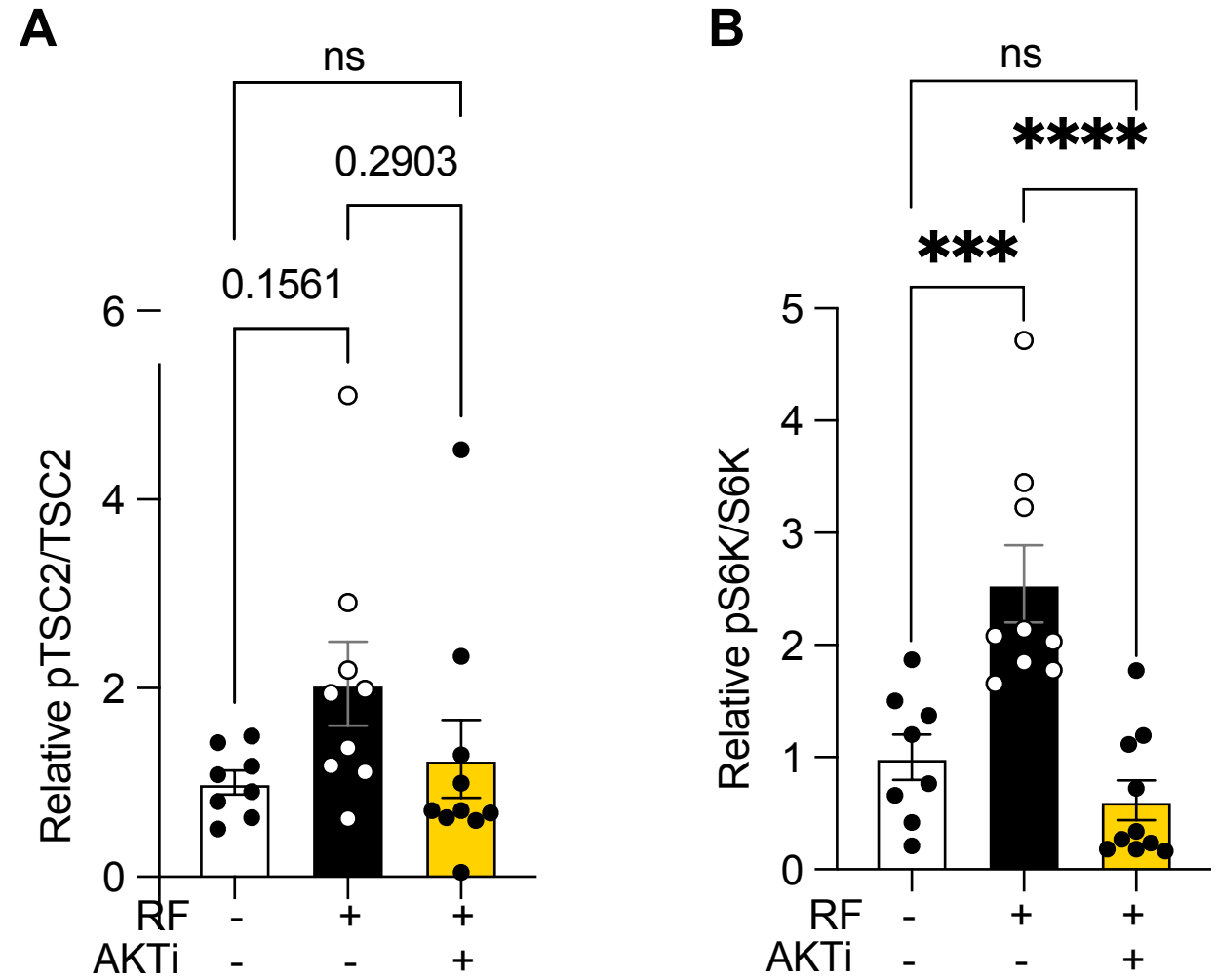

Supplemental Figure 1. Feeding induces AKT-dependent TSC2 phosphorylation and signaling in skeletal muscle. (A-B) Quantification of immunoblot data from Figure 1B and E, with relative phospho/total protein graphed as mean ± SEM. **(A,B)** Ordinary one-way ANOVA with Tukey's multiple comparison; ns $p > 0.05$, $*P < 0.05$, $**P < 0.01$, $***P < 0.001$, and $****P < 0.0001$.

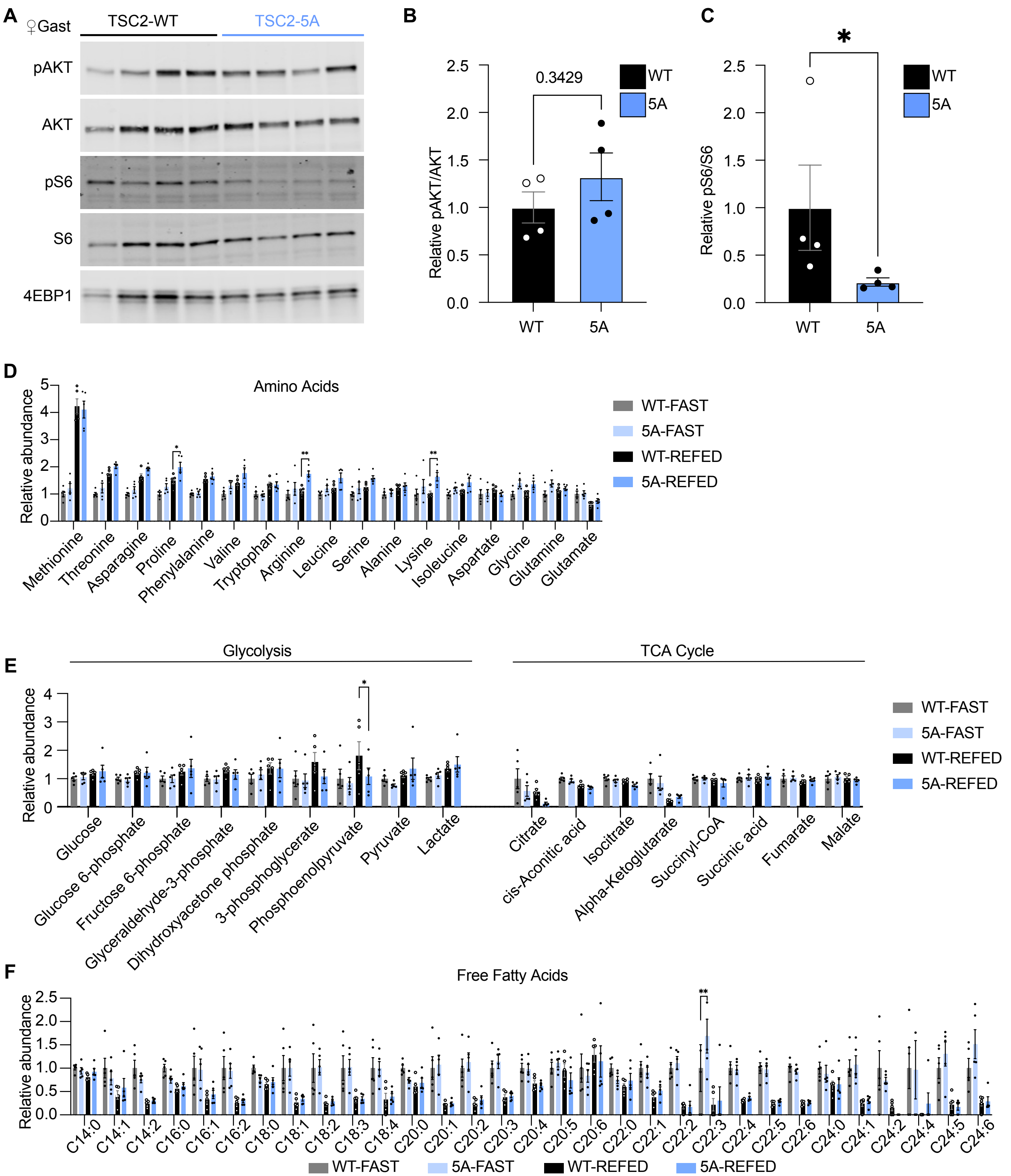
A
♀Gast
TSC2-WT
TSC2-5A
pAKT
AKT
pS6
S6
4EBP1
B
0.3429
WT
5A
Relative pAKT/AKT
C
*
Relative pS6/S6
D
Amino Acids
Relative abundance
WT-FAST
5A-FAST
WT-REFED
5A-REFED
Methionine
Threonine
Asparagine
Proline
Phenylalanine
Valine
Tryptophan
Arginine
Leucine
Serine
Alanine
Lysine
Isoleucine
Aspartate
Glycine
Glutamine
Glutamate
E
Glycolysis
TCA Cycle
Glucose
Glucose 6-phosphate
Fructose 6-phosphate
Glyceraldehyde-3-phosphate
Dihydroxyacetone phosphate
3-phosphoglycerate
Phosphoenolpyruvate
Pyruvate
Lactate
Citrate
cis-Aconitic acid
Isocitrate
Alpha-Ketoglutarate
Succinyl-CoA
Succinic acid
Fumarate
Malate
F
Free Fatty Acids
C14:0
C14:1
C14:2
C16:0
C16:1
C16:2
C18:0
C18:1
C18:2
C18:3
C18:4
C20:0
C20:1
C20:2
C20:3
C20:4
C20:5
C20:6
C22:0
C22:1
C22:2
C22:3
C22:4
C22:5
C22:6
C24:0
C24:1
C24:2
C24:4
C24:5
C24:6

Supplemental Figure 2. AKT-mediated phosphorylation of TSC2 does not significantly alter muscle metabolites following refeeding. (A-C) Immunoblots and quantification of protein extracts from gastrocnemius of 4-mos-old female SkM-TSC2-WT and -5A mice (n=4/group). **(B,C)** Quantification of immunoblot data from **(A)**, with relative phospho/total protein graphed as mean ± SEM. **(D-F)** Relative abundance of metabolites of quadriceps muscle of 3-mos-old male SkM-TSC2-WT and -5A mice from Figure 2I (n=5/group) graphed as mean ± SEM. **(D)** Proteinogenic amino acids, **(E)** glycolytic and TCA cycle intermediates, and **(F)** free fatty acids. **(B,C)** Mann-Whitney, **(D-F)** 2-way ANOVA with Tukey's multiple comparison. P >0.05, *P < 0.05, **P < 0.01, ***P < 0.001, and ****P < 0.0001 **(D-F)** differences between genotypes, but not feeding status, are shown.

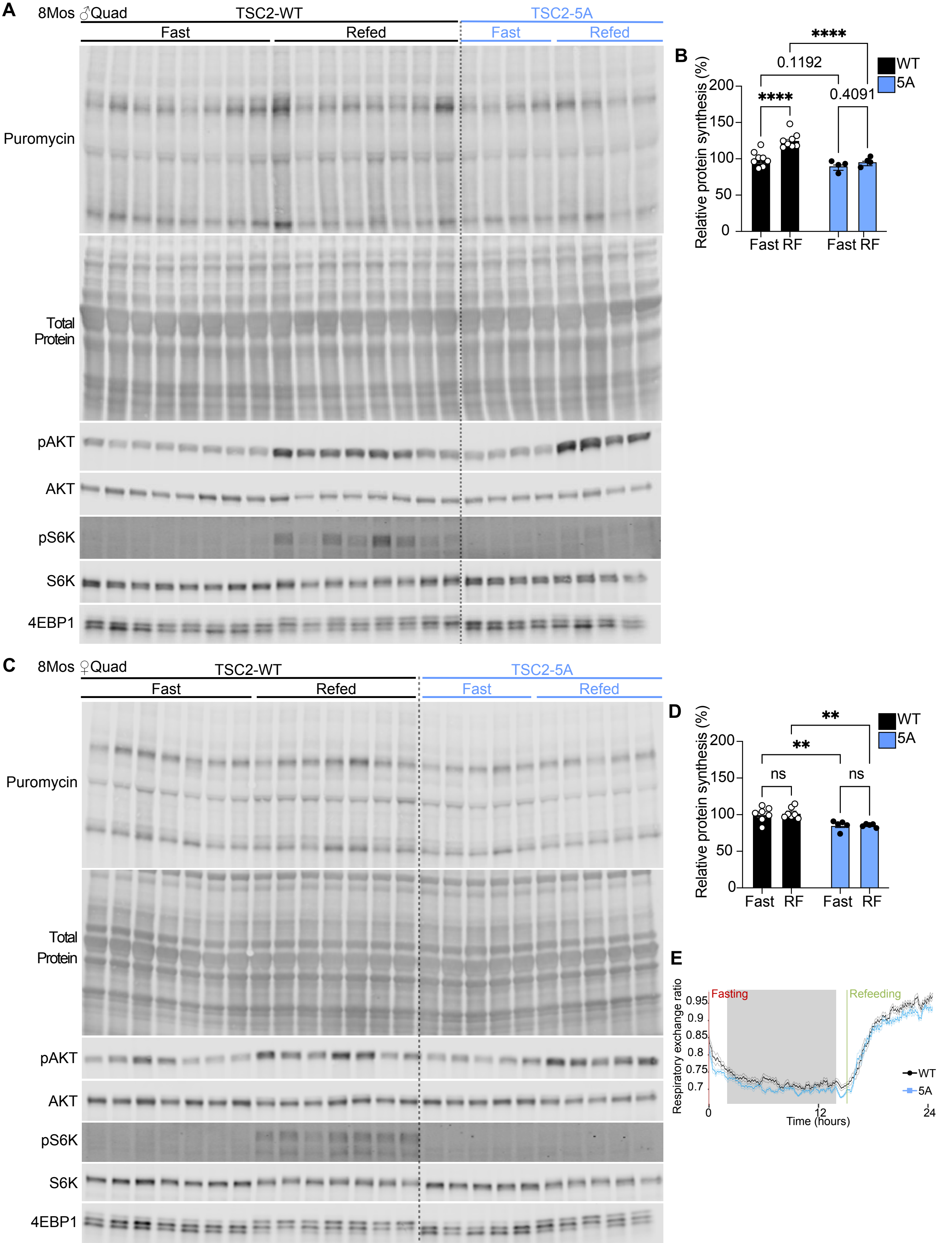

A
8Mos ♂Quad
TSC2-WT
TSC2-5A
Fast
Refed
Fast
Refed
Puromycin
Total Protein
pAKT
AKT
pS6K
S6K
4EBP1
B
Relative protein synthesis (%)
0
50
100
150
200
****
0.1192
****
0.4091
WT
5A
Fast RF
Fast RF
C
8Mos ♀Quad
TSC2-WT
TSC2-5A
Fast
Refed
Fast
Refed
Puromycin
Total Protein
pAKT
AKT
pS6K
S6K
4EBP1
D
Relative protein synthesis (%)
0
50
100
150
200
**
**
ns
ns
WT
5A
Fast RF
Fast RF
E
Respiratory exchange ratio
0.7
0.75
0.8
0.85
0.9
0.95
Fasting
Refeeding
WT
5A
0
12
24
Time (hours)

Supplemental Figure 3. Skeletal muscle protein synthesis is reduced in SkM-TSC2-5A mice despite a decreased induction by feeding. (A-B) Immunoblots and quantification of protein extracts from quadriceps of 8-mos-old male SkM-TSC2-WT and -5A mice after a 16 h overnight fast with or without 2 h RF (n=4-8/group). Puromycin injected (40 nmol/g bodyweight) 30 minutes prior to sacrifice. **(B)** Quantification of immunoblot data from **(A)**, with relative puromycin labeled protein/total protein graphed as mean ± SEM. **(C-D)** Representative immunoblots and quantification of protein extracts from gastrocnemius of 8-mos-old female mice after a 16 h overnight fast with or without 2 h RF (n=5-7/group). Puromycin injected (40 nmol/g bodyweight) 30 minutes prior to sacrifice. **(D)** Quantification of immunoblot data from **(C)**, with relative puromycin labeled protein/total protein graphed as mean ± SEM. **(E)** Respiratory exchange ratio of 6-mos-old male mice fasted for 16 h followed by ad libitum access to food as indicated (n=5,11). **(B,D)** 2-way ANOVA. ns $P > 0.05$, *$P < 0.05$, **$P < 0.01$, ***$P < 0.001$, and ****$P < 0.0001$.

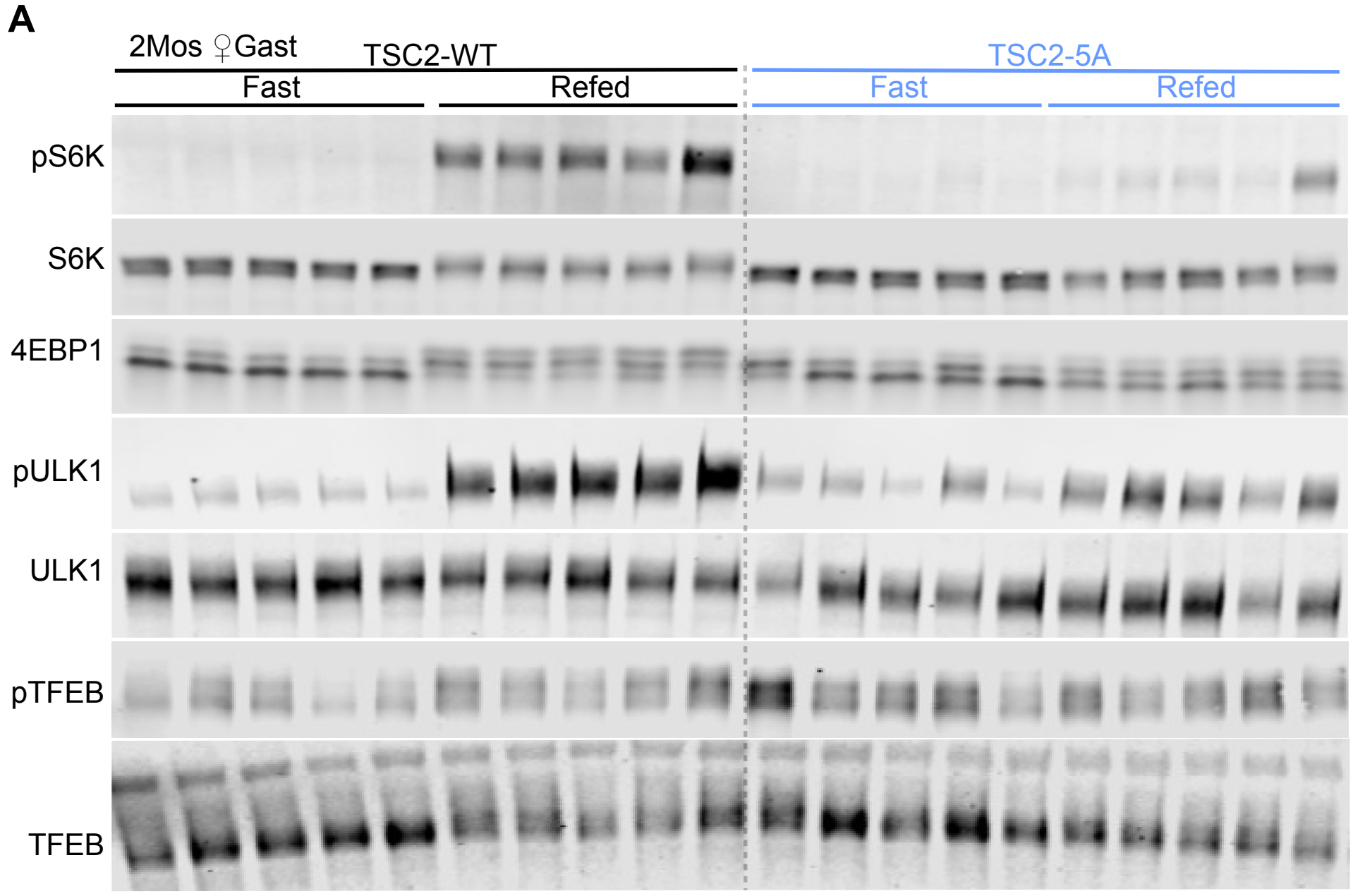

Supplemental Figure 4. AKT-mediated phosphorylation of TSC2 has substrate specific effects downstream of skeletal muscle mTORC1 signaling. (A) Representative immunoblots of protein extracts from gastrocneimus muscle of female 2-mos-old mice after a 16 h overnight fast with or without 2 h RF (n=5-7/group) quantified in Figure 4E-G.

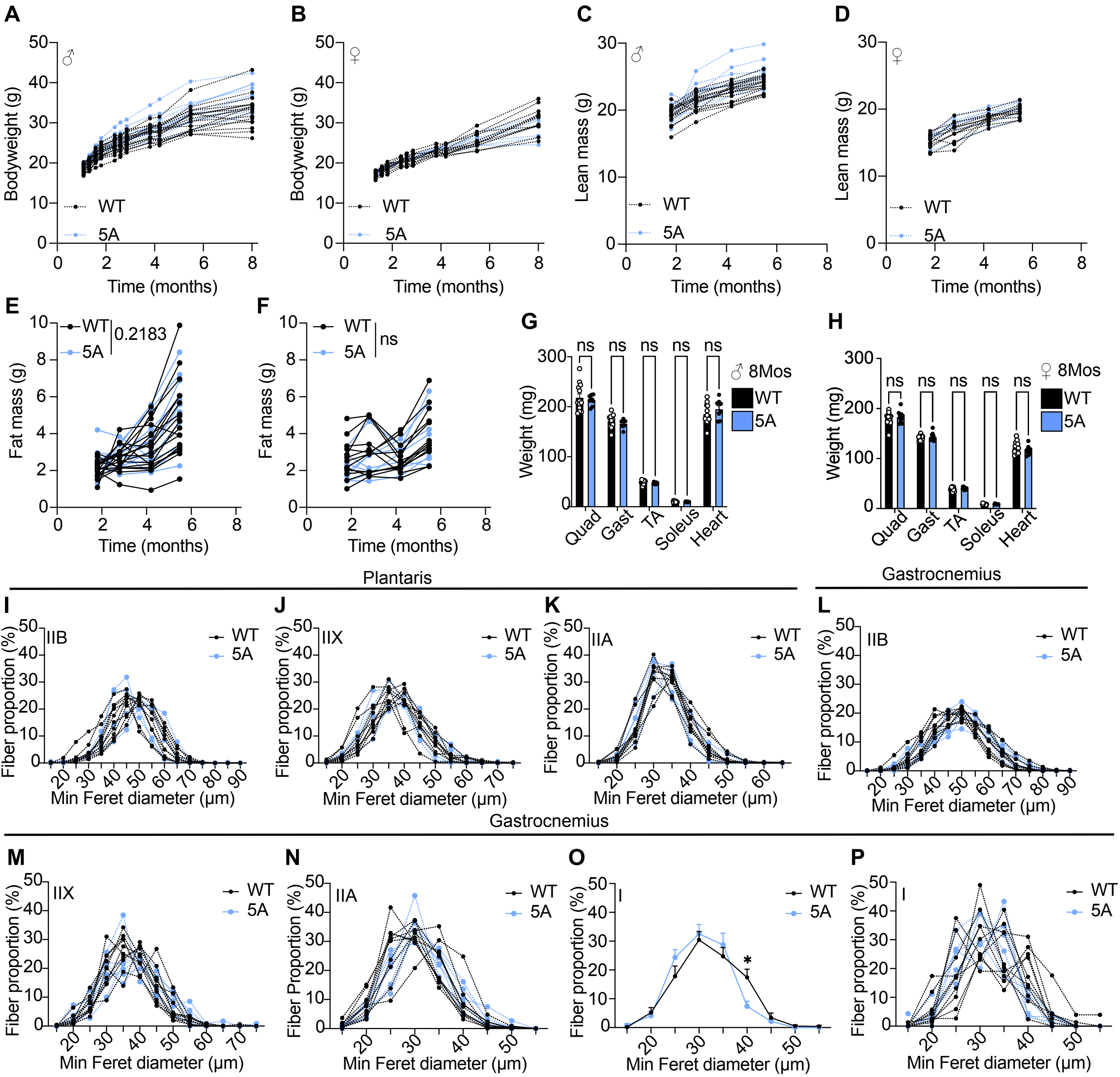

Supplemental Figure 5. SkM-TSC2-WT and -5A mice have no difference in fat mass or muscle weight. **(A-B)** Bodyweight over 8 mos graphed as individual mice in **(A)** male (n=11-19) and **(B)** female mice (n=7-13). **(C-D)** Lean mass over 6 mos graphed as individual mice in **(C)** male (n=11-18) and **(D)** female mice (n=7-13). **(E-F)** Fat mass over 6 mos graphed as individual mice of **(E)** male (n=11-18) and **(F)** female mice (n=7-13). **(G-H)** Muscle weights for quadriceps (Quad), gastrocnemius + plantaris (Gast), tibialis anterior (TA), soleus, and heart in 8-mos-old **(G)** male (n=10-11) and **(H)** female (n=10-14) mice pooled from both 16 h overnight fasted with or without 2 h RF displayed as mean ± SEM. **(I-P)** Myofiber minimum Feret diameter for **(I-K)** plantaris: **(I)** Type IIB, **(J)** Type IIX, and **(K)** Type IIA graphed as individual mice. **(L-P)** gastrocnemius: **(L)** Type IIB, **(M)** Type IIX, **(N)** Type IIA graphed as individual mice **(O-P)** Type I **(O)** graphed as mean ± SEM and **(P)** graphed as individual mice. **(E,O)** 2-way ANOVA, **(F)** Mixed-effects analysis, **(G-H)** Multiple Mann-Whitney with Holm-Šídák correction; ns $P > 0.05$, $^*P < 0.05$, $^{**}P < 0.01$, $^{***}P < 0.001$, and $^{****}P < 0.0001$.

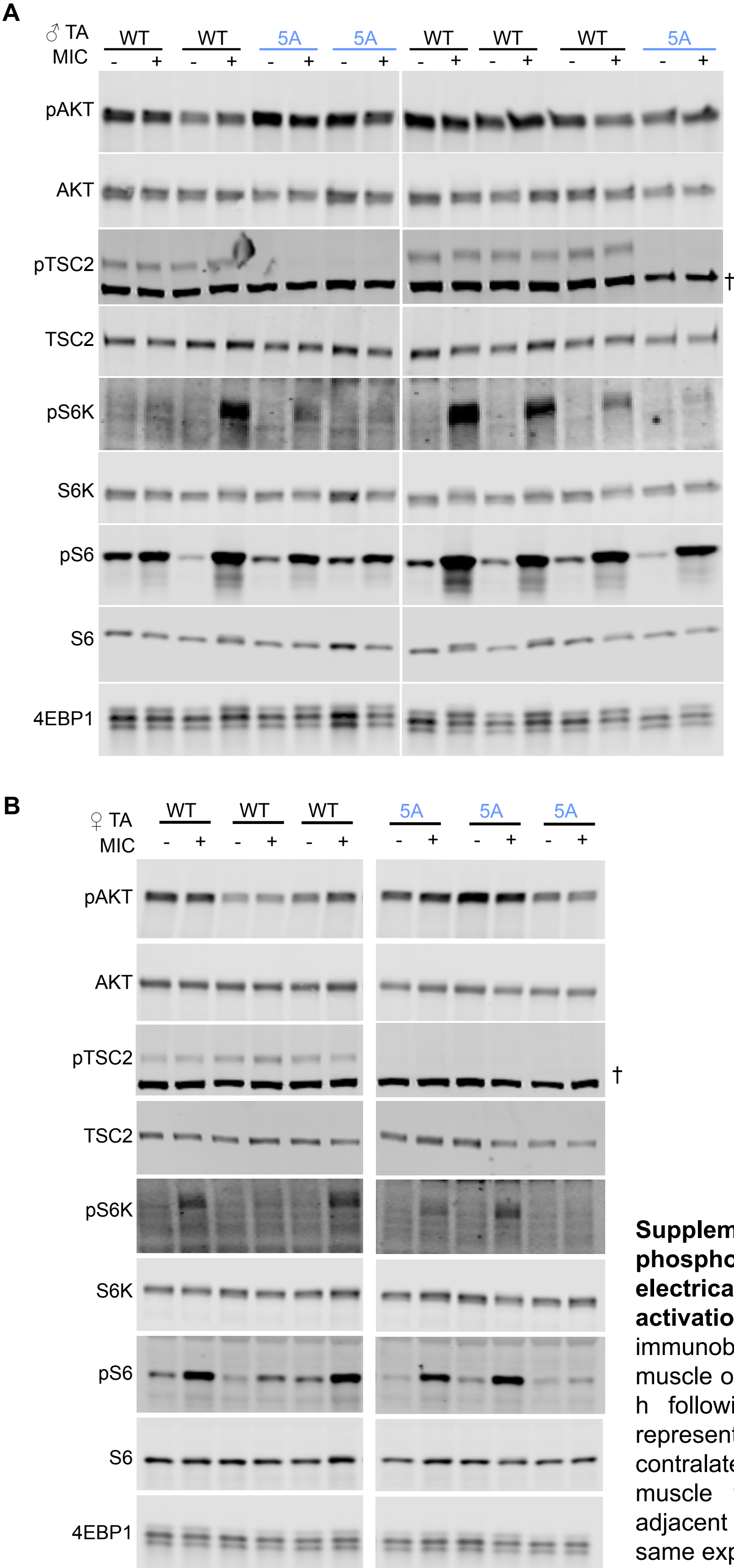

Supplemental Figure 6. AKT-mediated phosphorylation of TSC2 is dispensable for electrical stimulation-mediated mTORC1 activation in female mice. (A-B) Representative immunoblots of protein extracts from tibialis anterior muscle of 3-mos-old **(A)** male and **(B)** female mice 3 h following MIC treatment. Each "WT" or "5A" represents one animal with "-" indicating unstimulated contralateral muscle and "+" indicating the stimulated muscle from the same mouse. Images shown adjacent to each other are from the same blot at the same exposure. † denotes non-specific band.

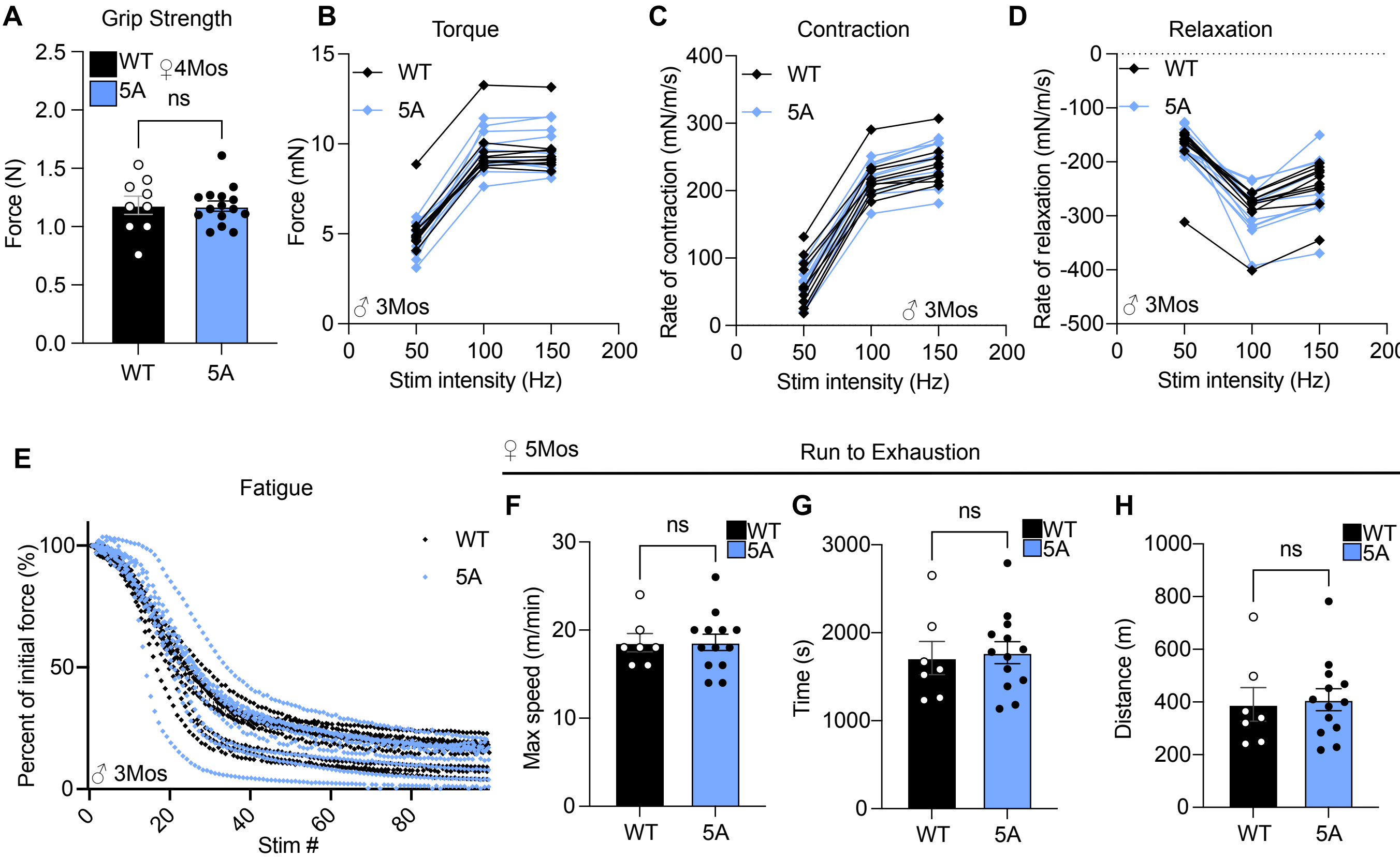

Supplemental Figure 7. SkM-TSC2-5A female mice do not demonstrate alterations in strength or endurance capacity. (A) Four-limb grip strength of female 4-mos-old mice (n=9-15) **(B-D)** Electrical stimulation of tibial nerve in vivo for functional assessment of plantarflexors in 3-mos-old male mice at indicated stimulation intensities represented as individual mice (n=10/group): **(B)** isometric torque, **(C)** rate of contraction, and **(D)** rate of relaxation. **(E)** Male 3-mos-old mice treated with repeated submaximal, 50 Hz, stimulations to measure fatigue resistance represented as individual mice as a percentage of initial force of first stimulation at 50 Hz. **(F-H)** Female 5-mos-old mice run to exhaustion assessed via graded exercise stress test assay represented as mean ± SEM (n=7-13): **(F)** maximal speed reached, **(G)** time to exhaustion, and **(H)** final distance reached. **(A,F-H)** Mann Whitney. ns $P > 0.05$, $^{*}P < 0.05$, $^{**}P < 0.01$, $^{***}P < 0.001$, and $^{****}P < 0.0001$.

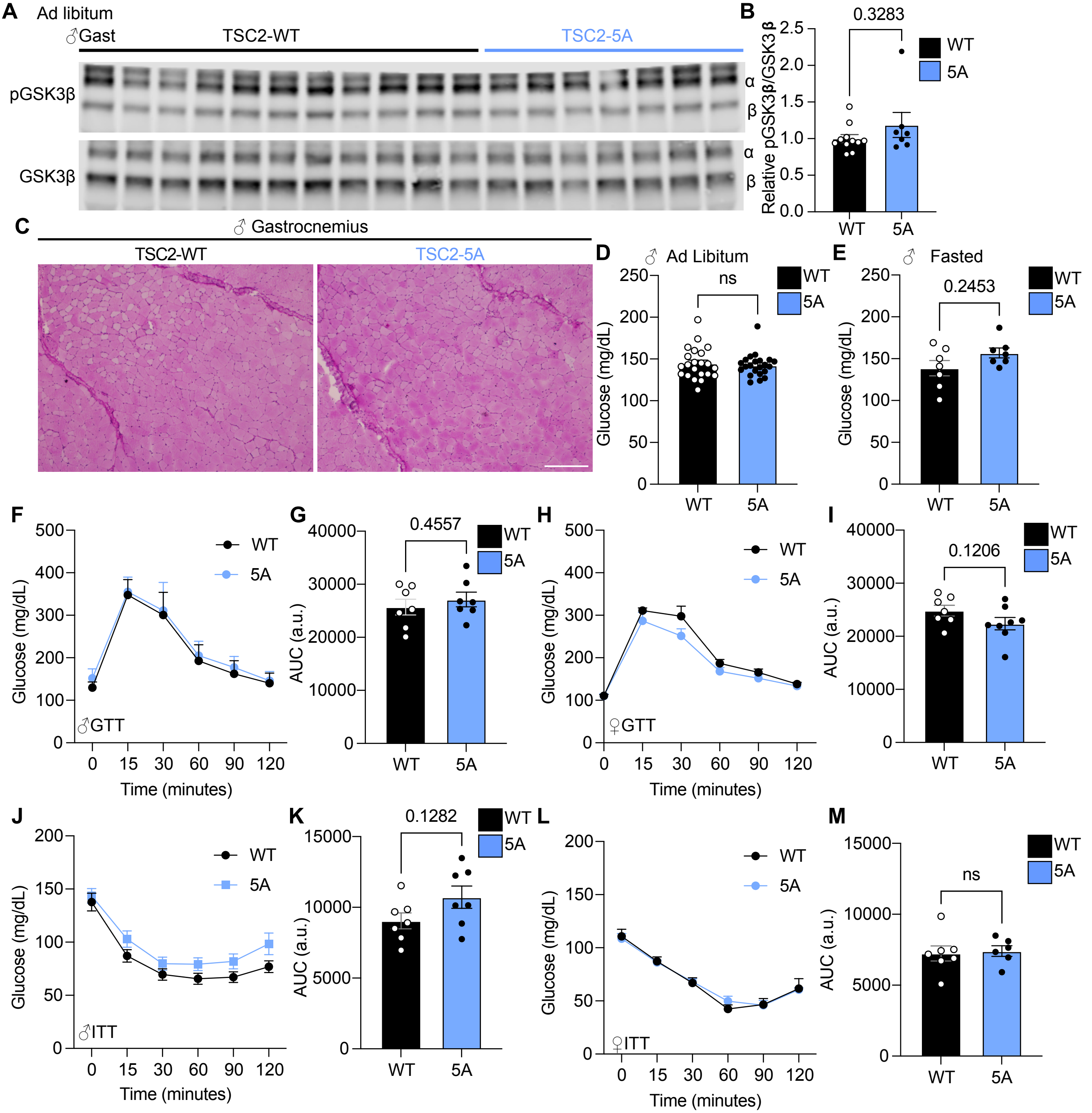

Supplemental Figure 8. SkM-TSC2-5A mice do not exhibit overt differences in glucose metabolism. (A-B) Immunoblots and quantification of protein extracts from gastrocnemius of male 4-mos-old SkM-TSC2-WT and -5A mice fed ad libitum (n=7-11). **(B)** Quantification of immunoblot data from **(A)**, with relative phospho/total protein graphed as mean ± SEM. **(C)** Representative images of Periodic Acid-Schiff staining for glycogen in gastrocnemius of male 4-mos-old SkM-TSC2-WT and -5A mice fed ad libitum. Scale bar indicates 200 µm. **(D-E)** Blood glucose of male 3-mos-old SkM-TSC2-WT and -5A mice represented as mean ± SEM for either (D) ad libitum (n=22/group) or **(E)** 6 h fasted (n=7/group). **(F-I)** Glucose tolerance tests (GTT) in 3-mos-old SkM-TSC2-WT and -5A mice injected with glucose (2 mg/g bodyweight) following a 6 h daytime fast represented as mean ± SEM for **(F-G)** males (n=7/group) with **(F)** GTT and **(G)** area under the curve (AUC) and **(H-I)** female (n=7-8/group) with **(H)** GTT and (I) AUC. **(J-M)** Insulin tolerance tests (ITT) in 3-mos-old SkM-TSC2-WT and -5A mice injected with insulin (0.5 U/kg bodyweight) following a 6 h daytime fast represented as mean ± SEM for **(J-K)** male (n=7/group) with **(J)** ITT and **(K)** AUC and **(L-M)** female (n=6-7/group) with **(L)** ITT and **(M)** AUC. **(B,D-E,G,I,K,M)** Mann Whitney. ns P >0.05, *P < 0.05, **P < 0.01, ***P < 0.001, and ****P < 0.0001.
