# Supplementary material for: Feeding-induced muscle mTORC1 signaling regulates postprandial protein synthesis and endurance but not muscle size
Source: JCI Insight. 2026 Sep 10;11(20):e210523. doi: 10.1172/jci.insight.210523 (PMC13726562; doi:10.1172/jci.insight.210523)

Figure 1B,C,D
Supplemental Figure 1A,B

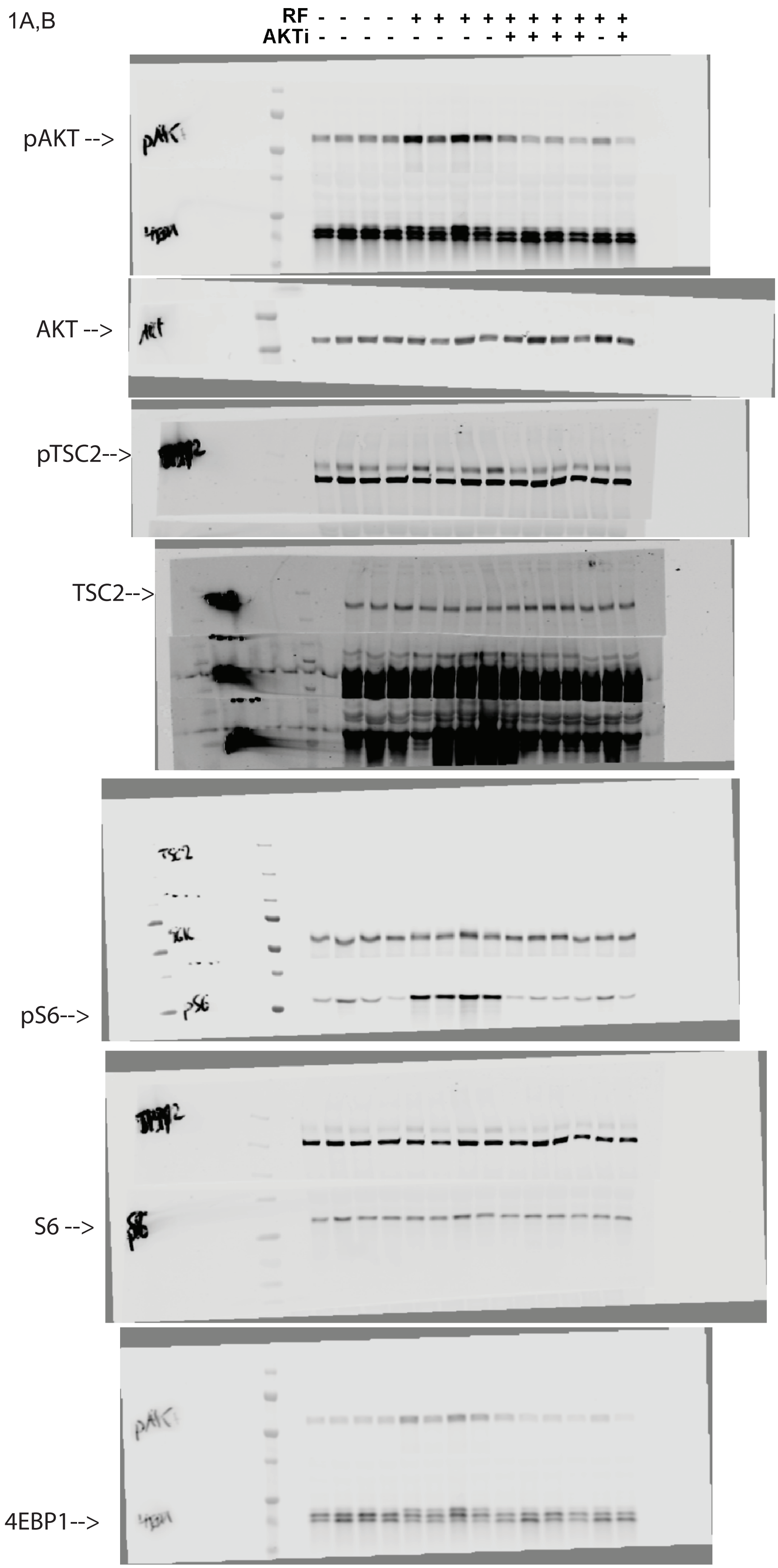

Figure 1C,D,E
Supplemental Figure 1A

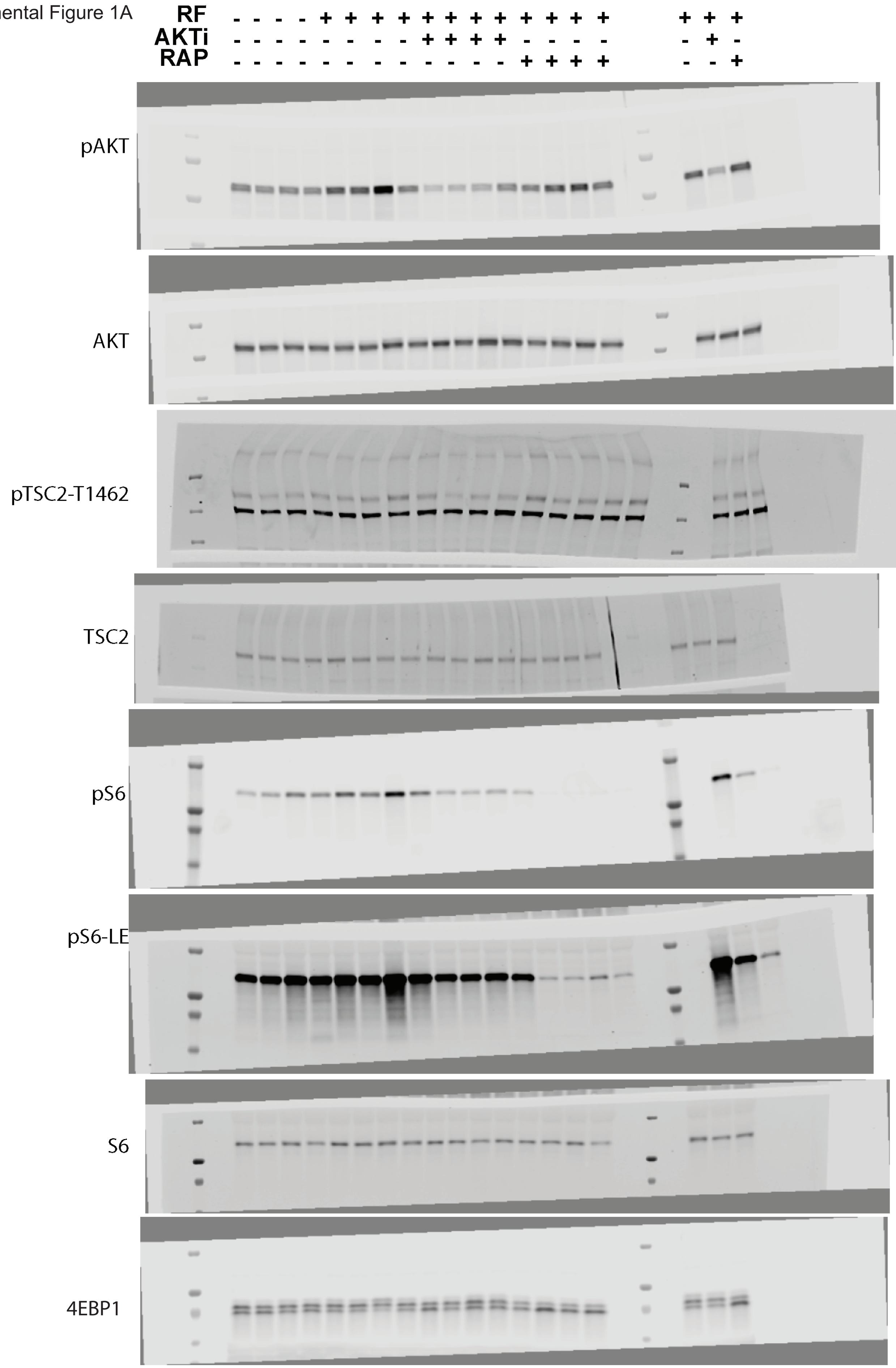

Figure 1B and S1B

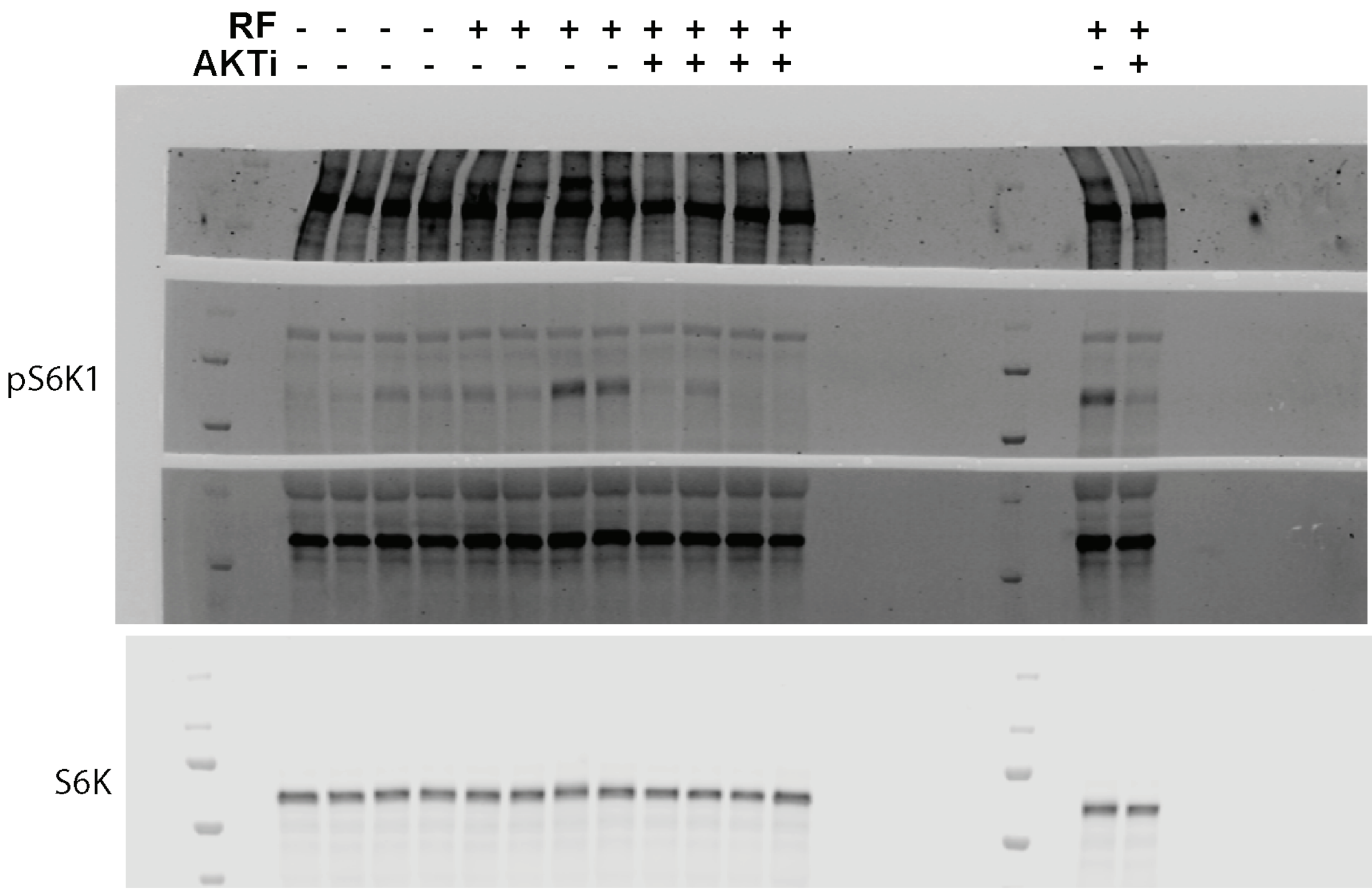

Figure S1B

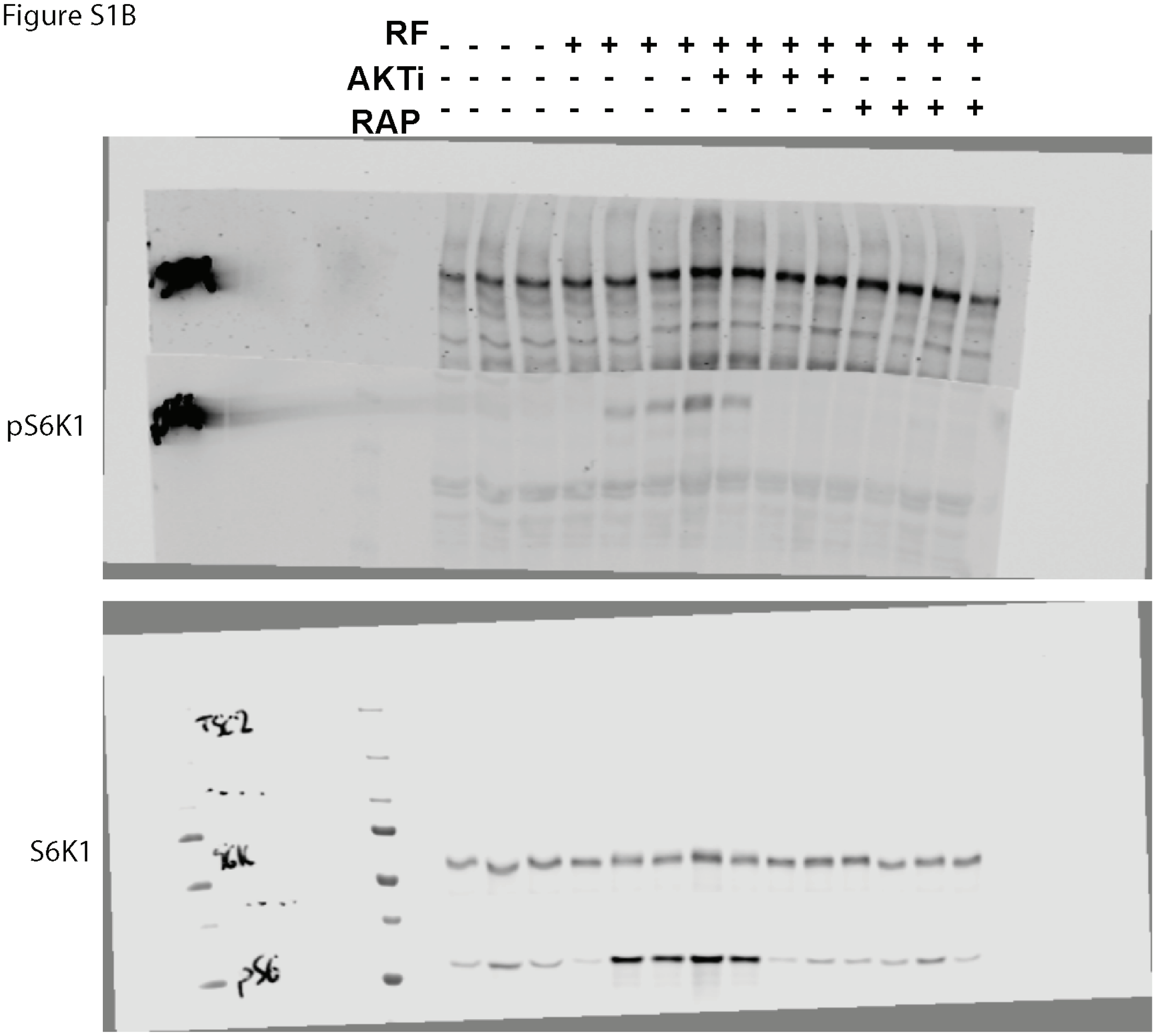

Figure 1E,F

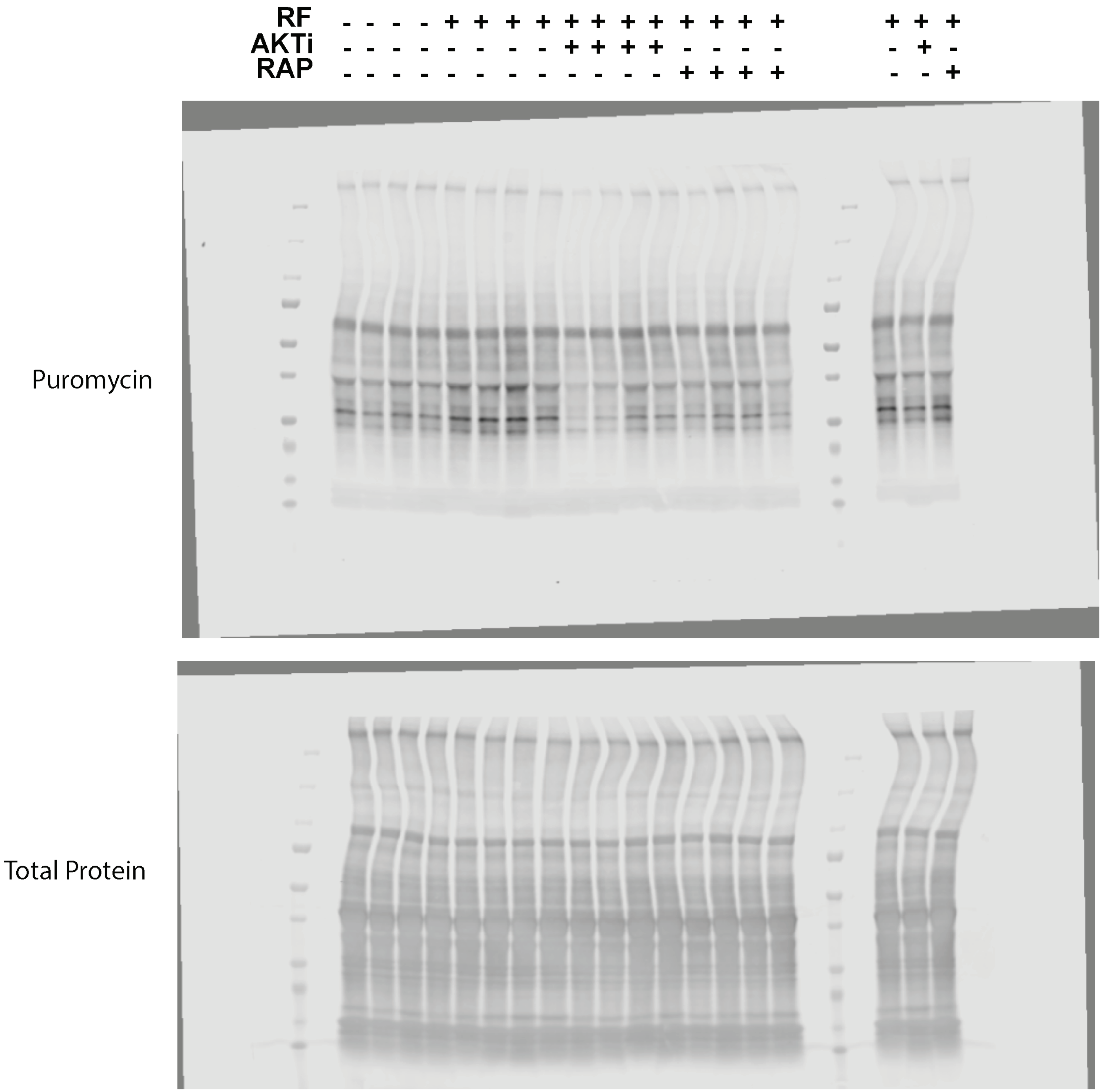

Figure 1F

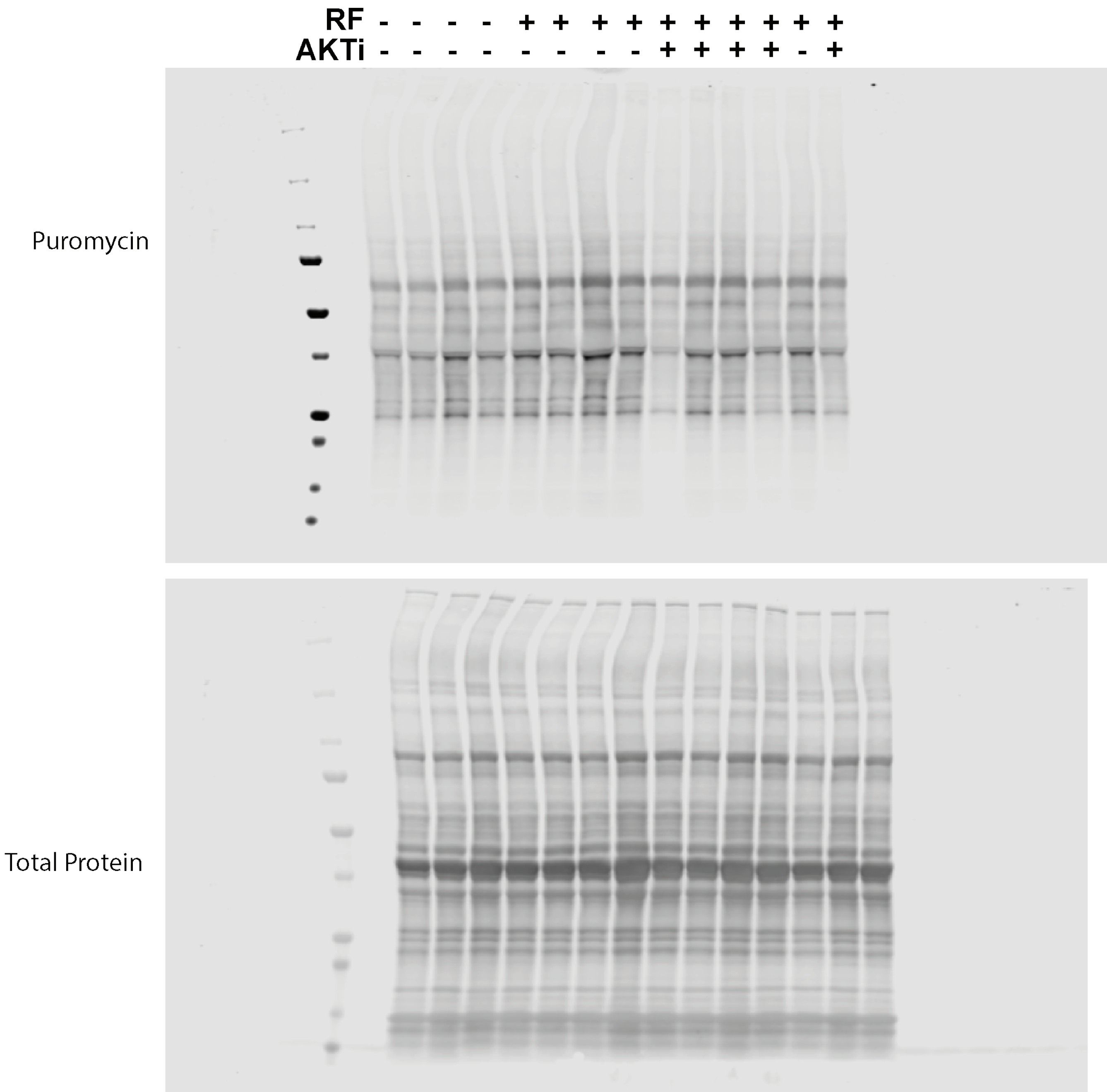

Figure 2C-E

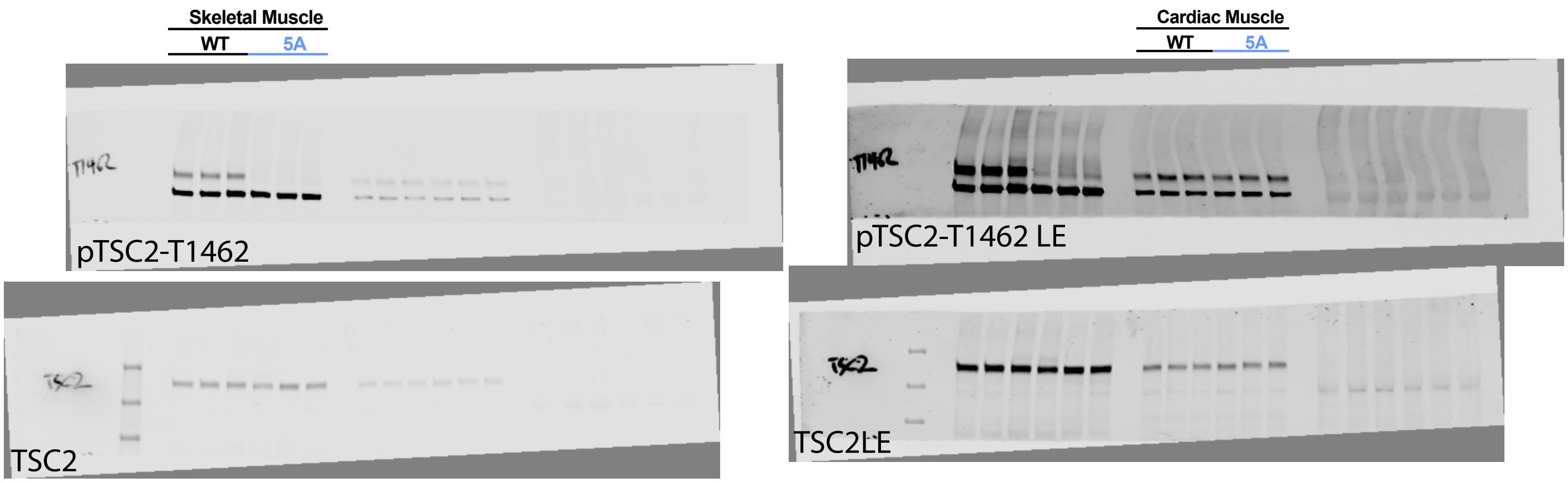
Skeletal Muscle
WT
5A
T1462
pTSC2-T1462
TSC2
TSC2
Cardiac Muscle
WT
5A
T1462
pTSC2-T1462 LE
TSC2
TSC2LE

Figure 2F-H

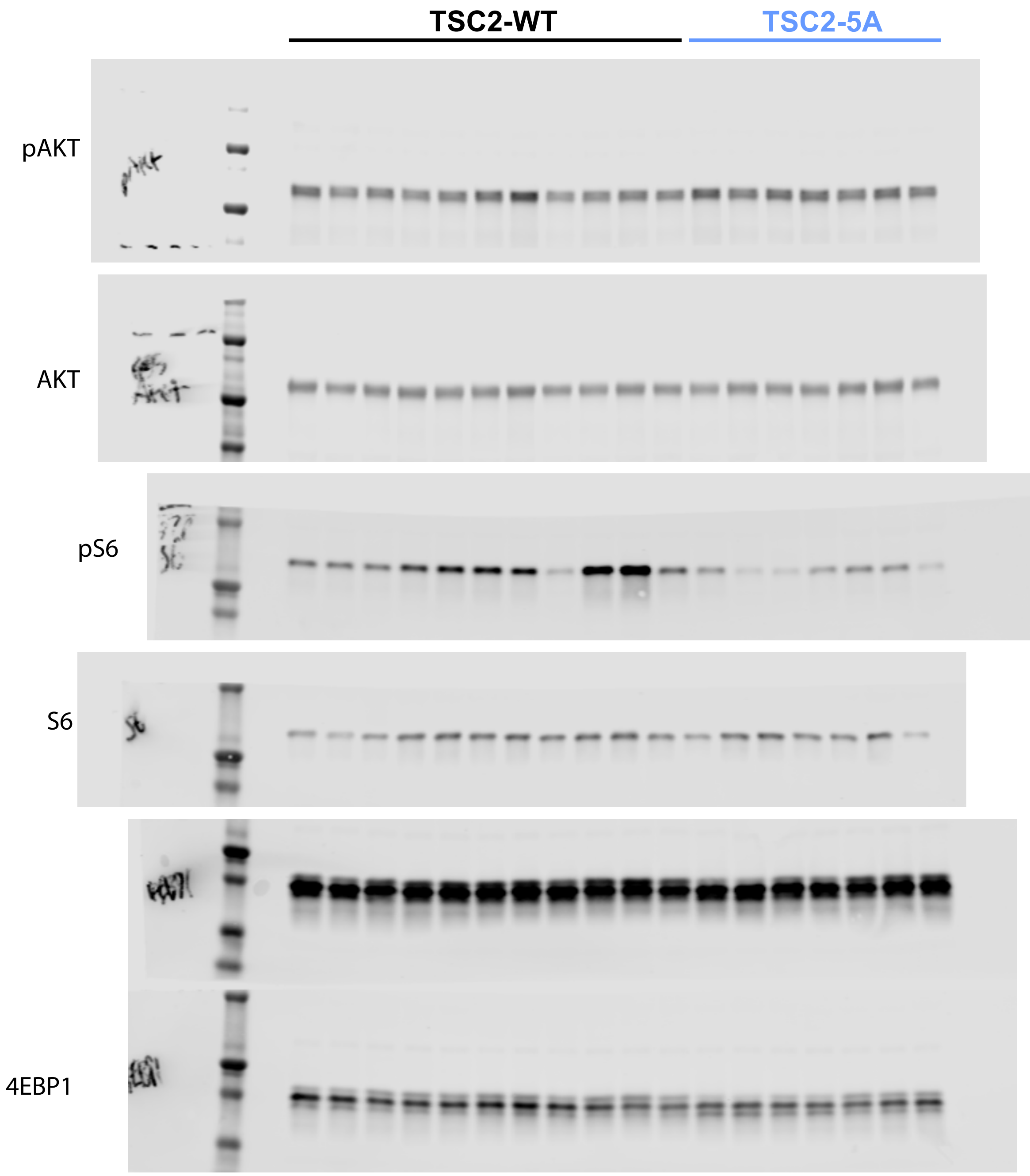

Figure 2I-K

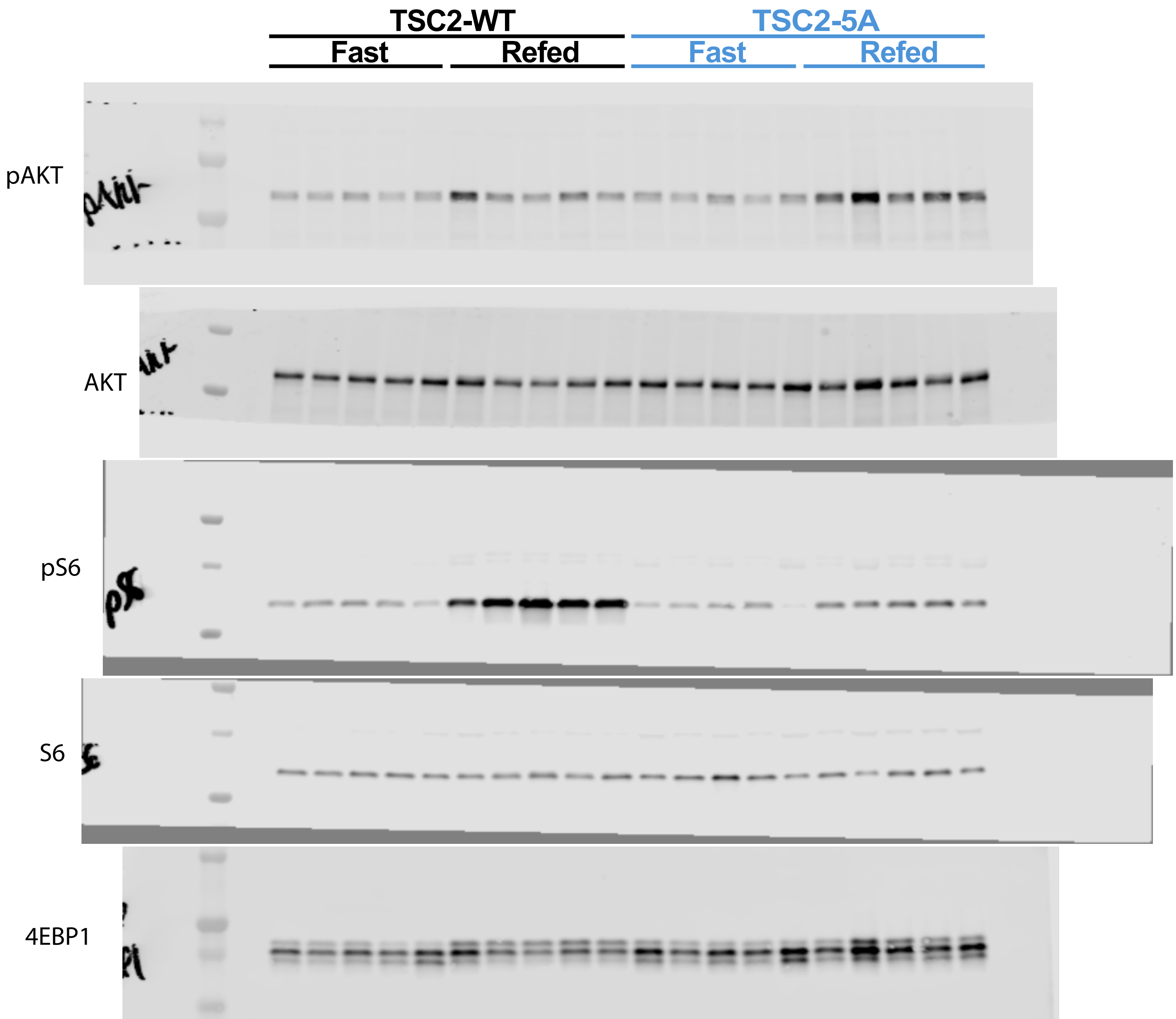

Figure S2A-C

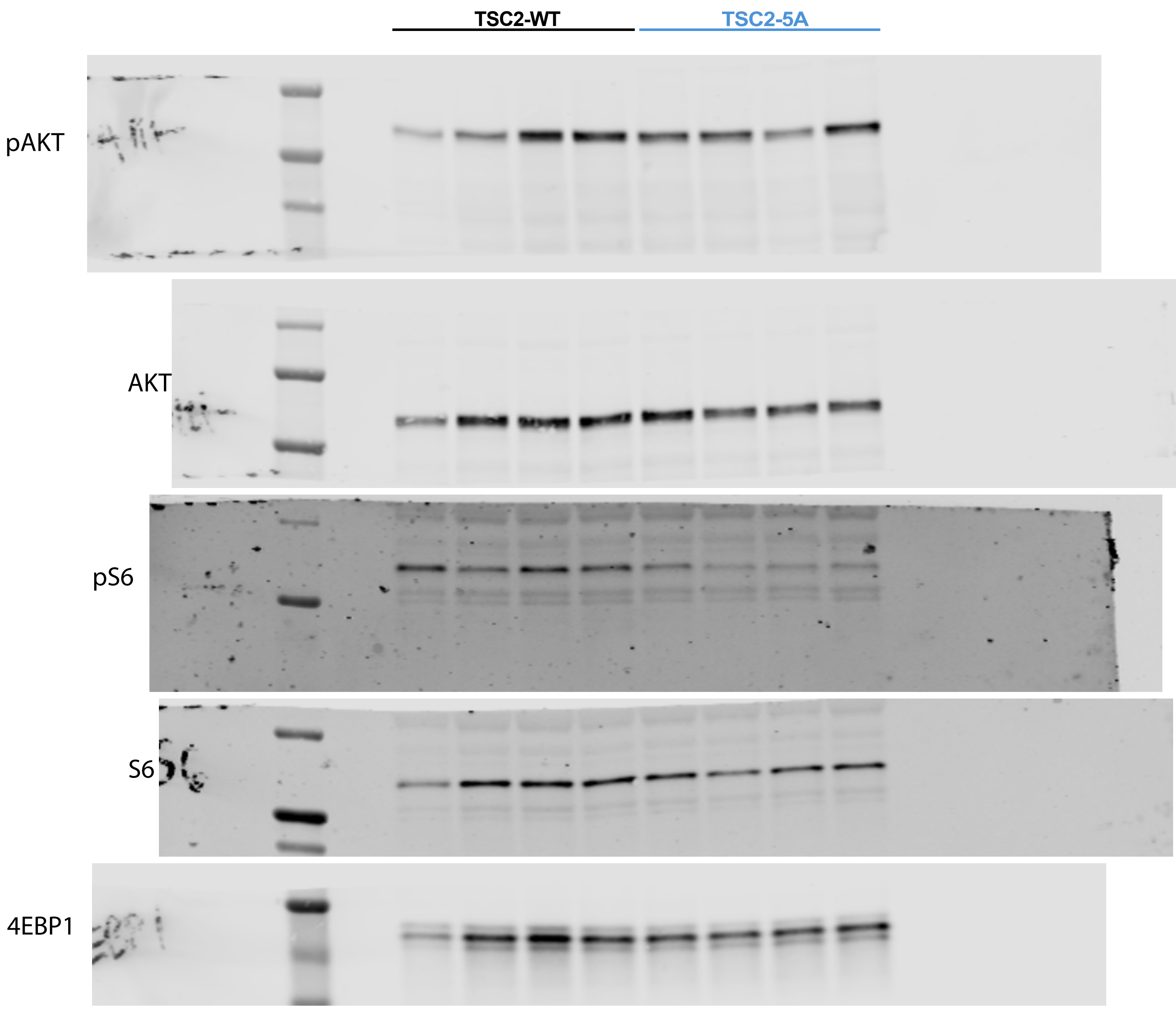

Figure 3A,B

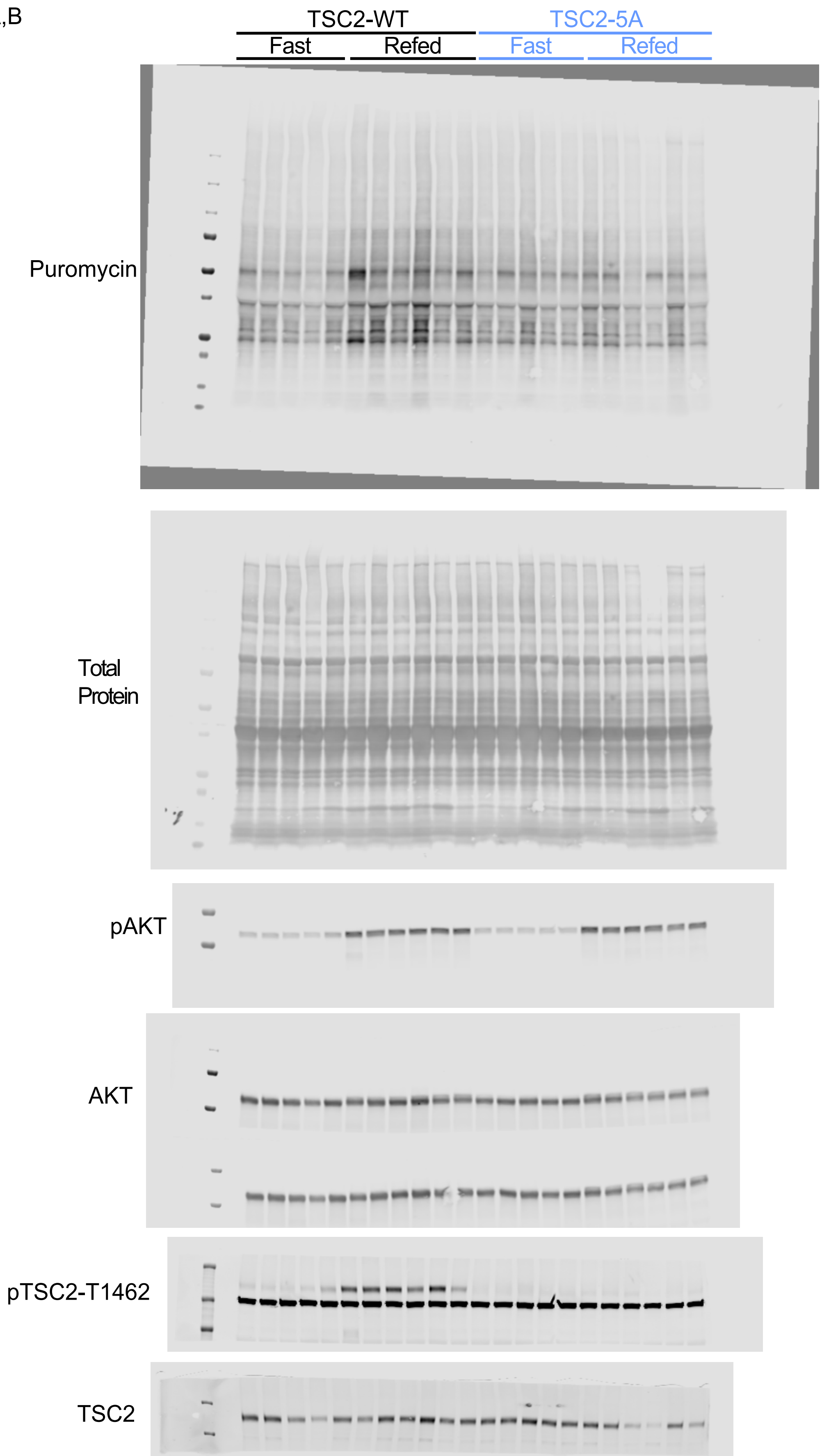
TSC2-WT
TSC2-5A
Fast
Refed
Fast
Refed
Puromycin
Total Protein
pAKT
AKT
pTSC2-T1462
TSC2

Figure 3C,D

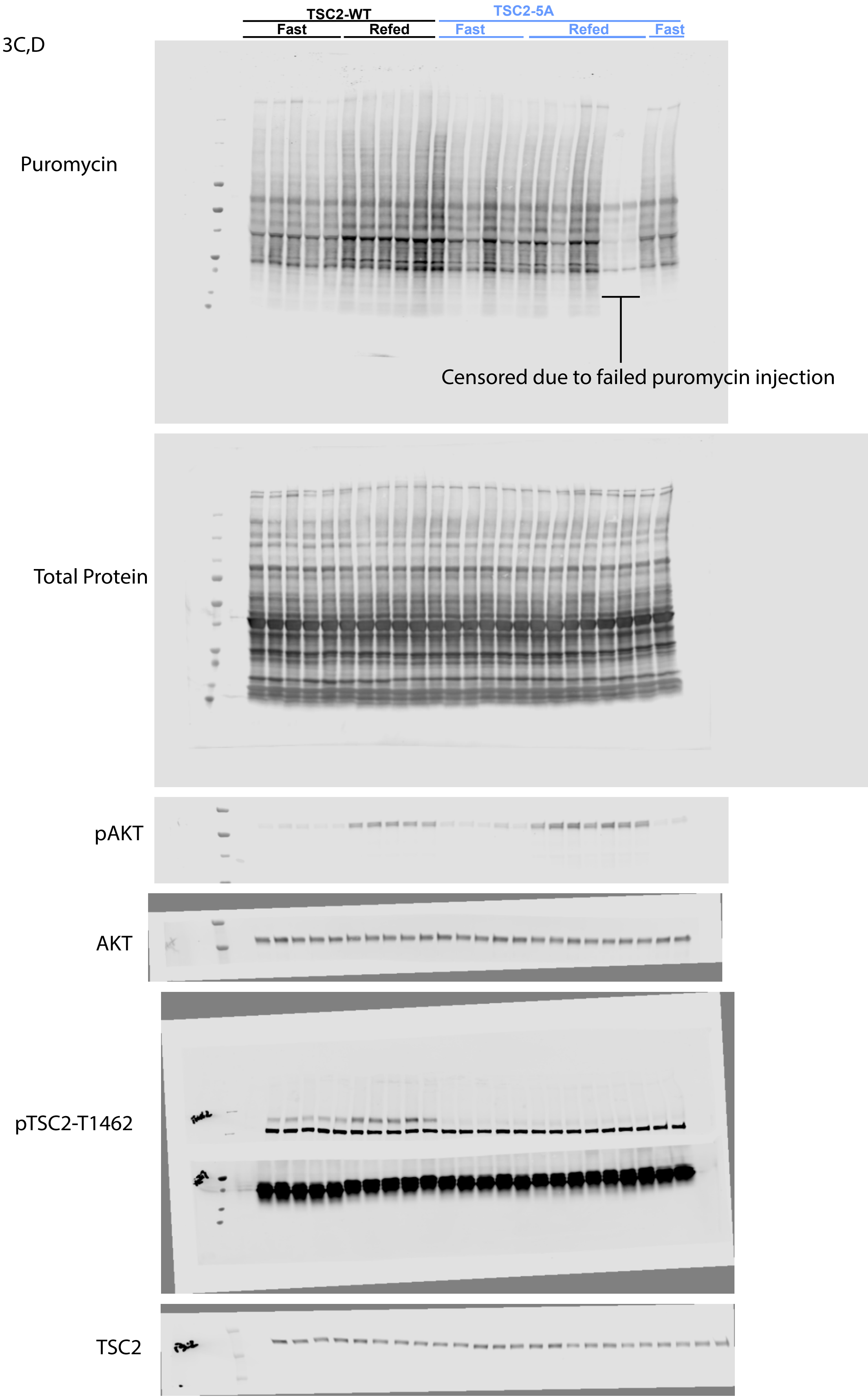
TSC2-WT
Fast
Refed
TSC2-5A
Fast
Refed
Fast
Puromycin
Censored due to failed puromycin injection
Total Protein
pAKT
AKT
pTSC2-T1462
TSC2

Supplemental Figure 3A,B

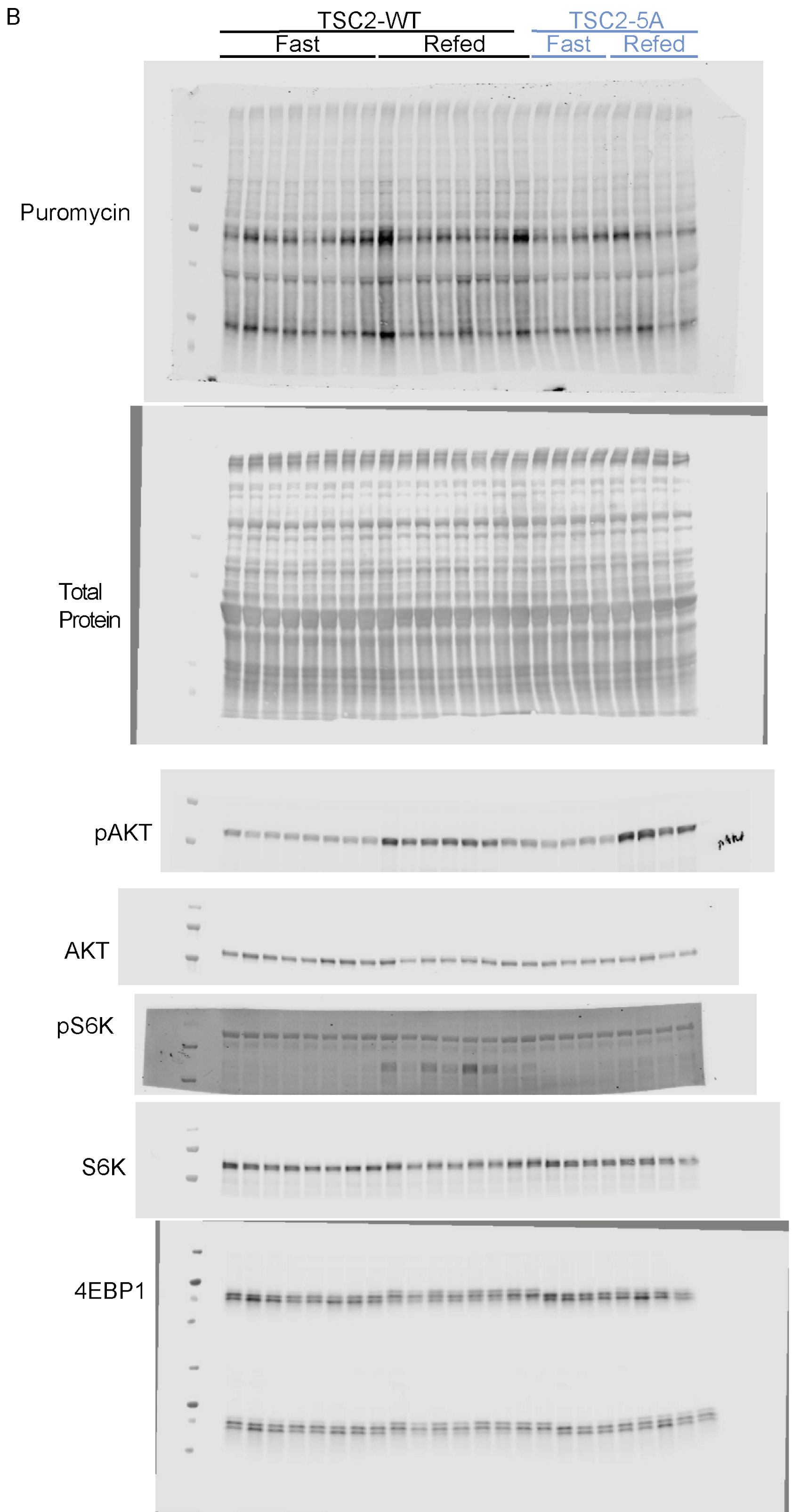

Supplemental Figure 3C,D

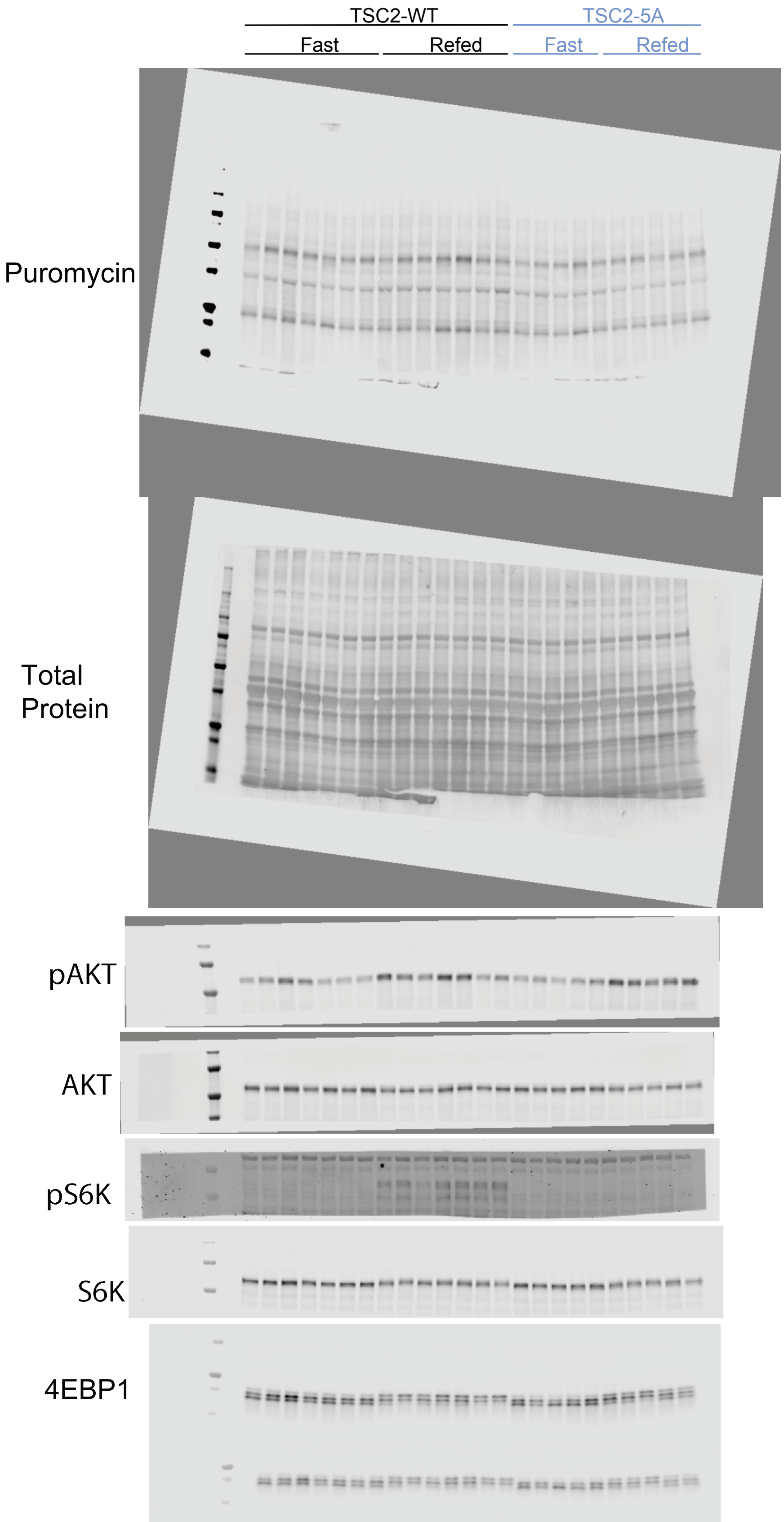

Figure 4A-D

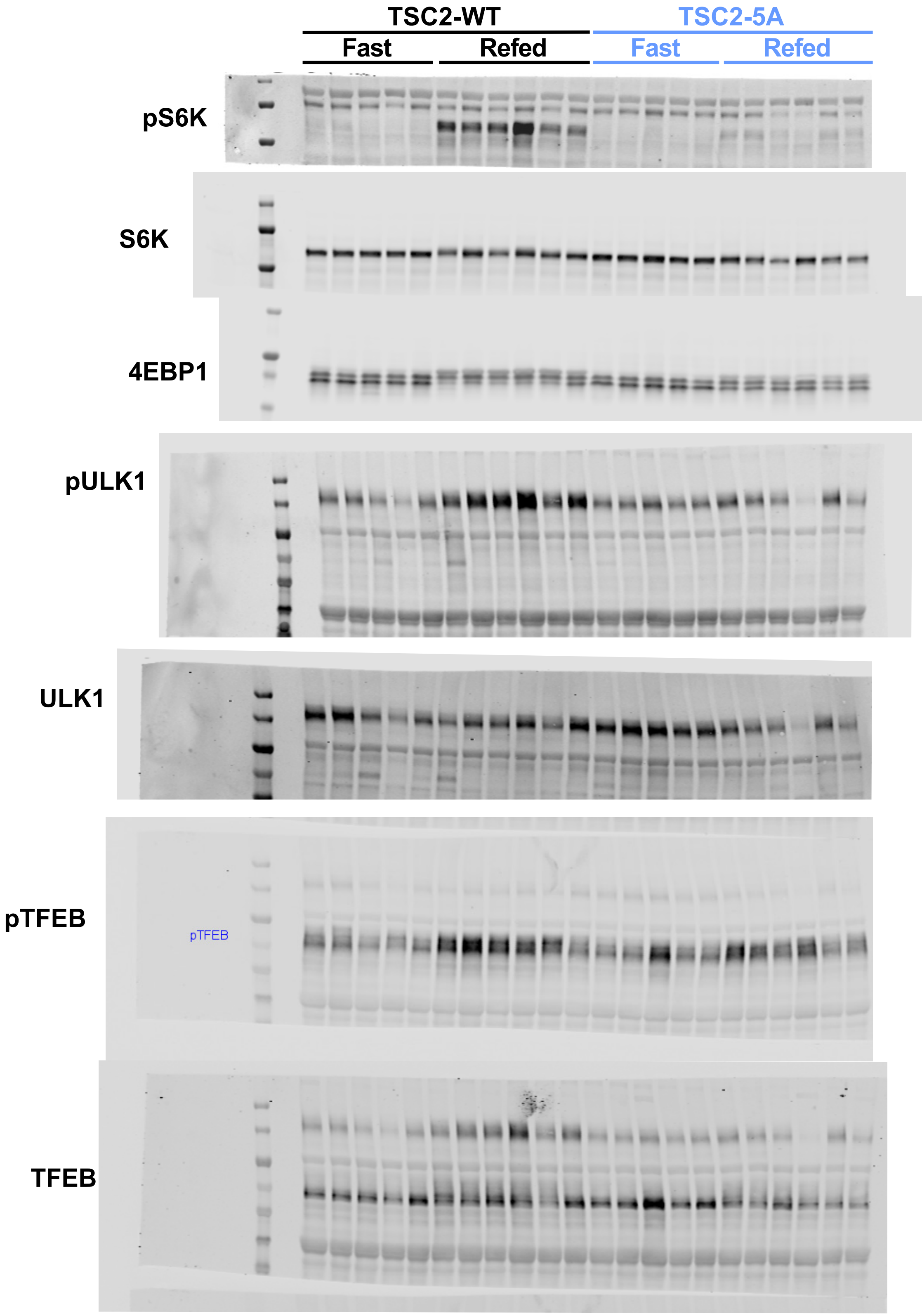
TSC2-WT
TSC2-5A
Fast
Refed
Fast
Refed
pS6K
S6K
4EBP1
pULK1
ULK1
pTFEB
pTFEB
TFEB

Figure 4E-G
Supplemental Figure 4A

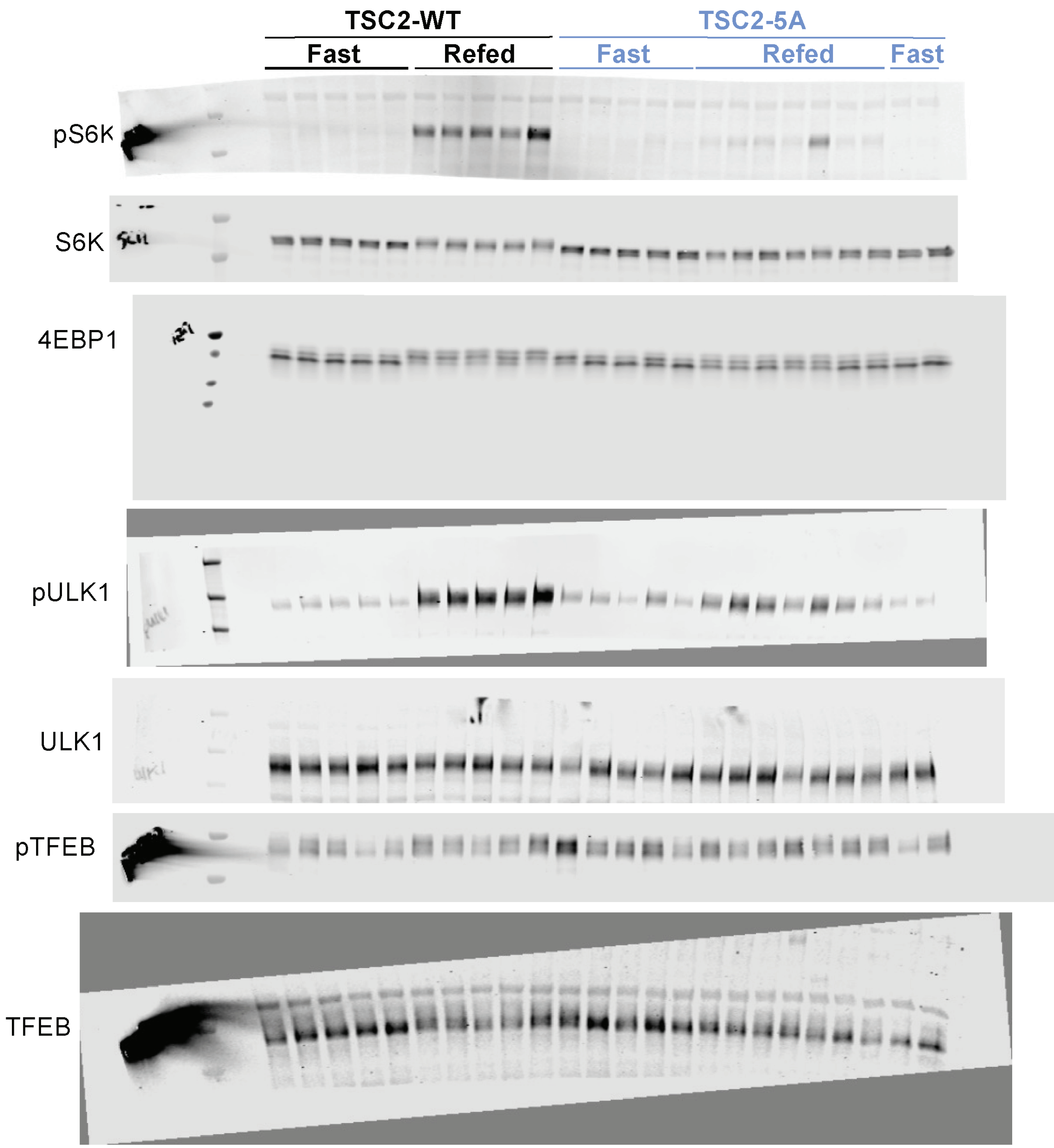

Figure 7B,C,D,G,H
Supplemental Figure 6A

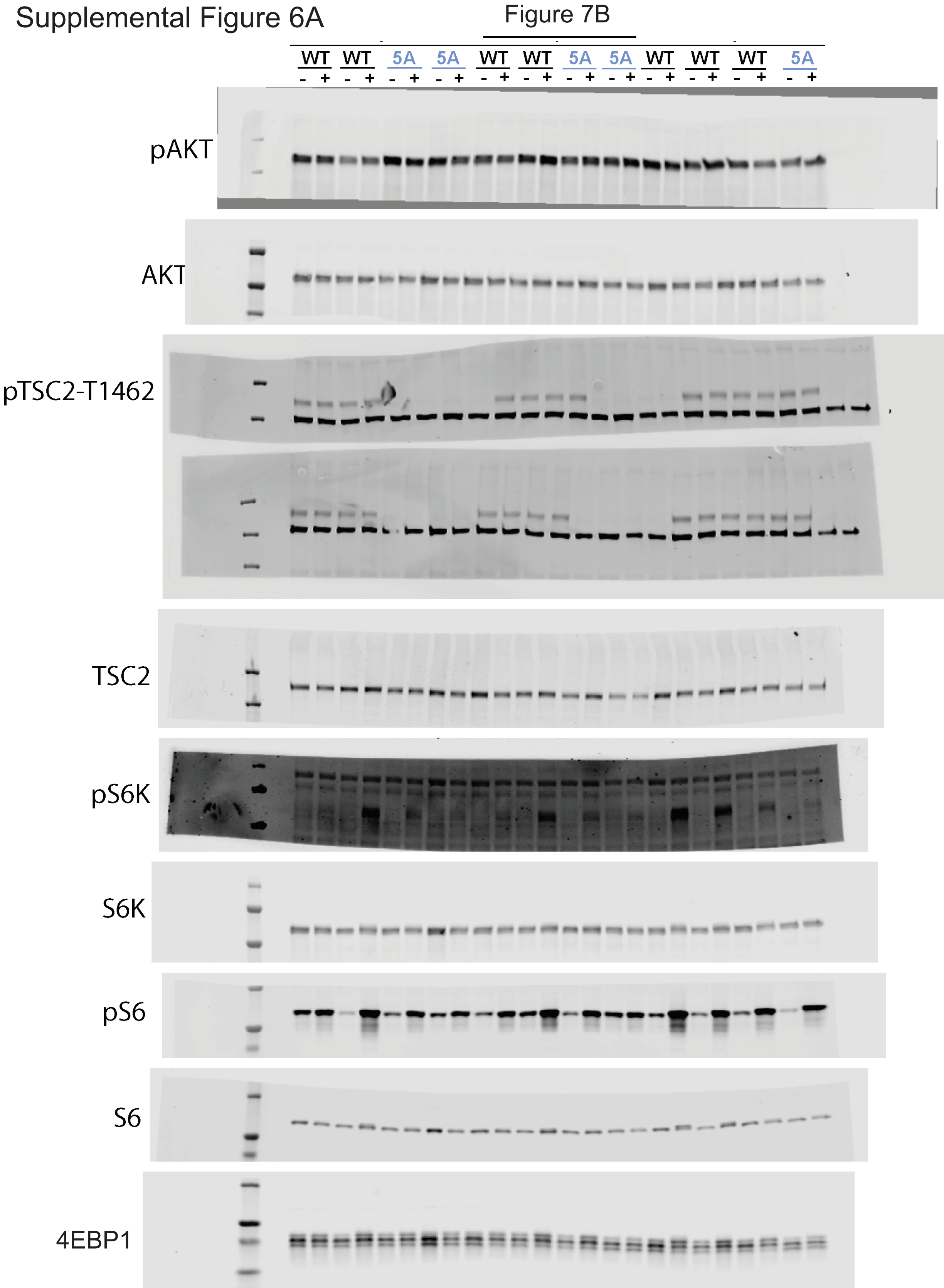

Figure 7E,F,I,J
Supplemental Figure 6B

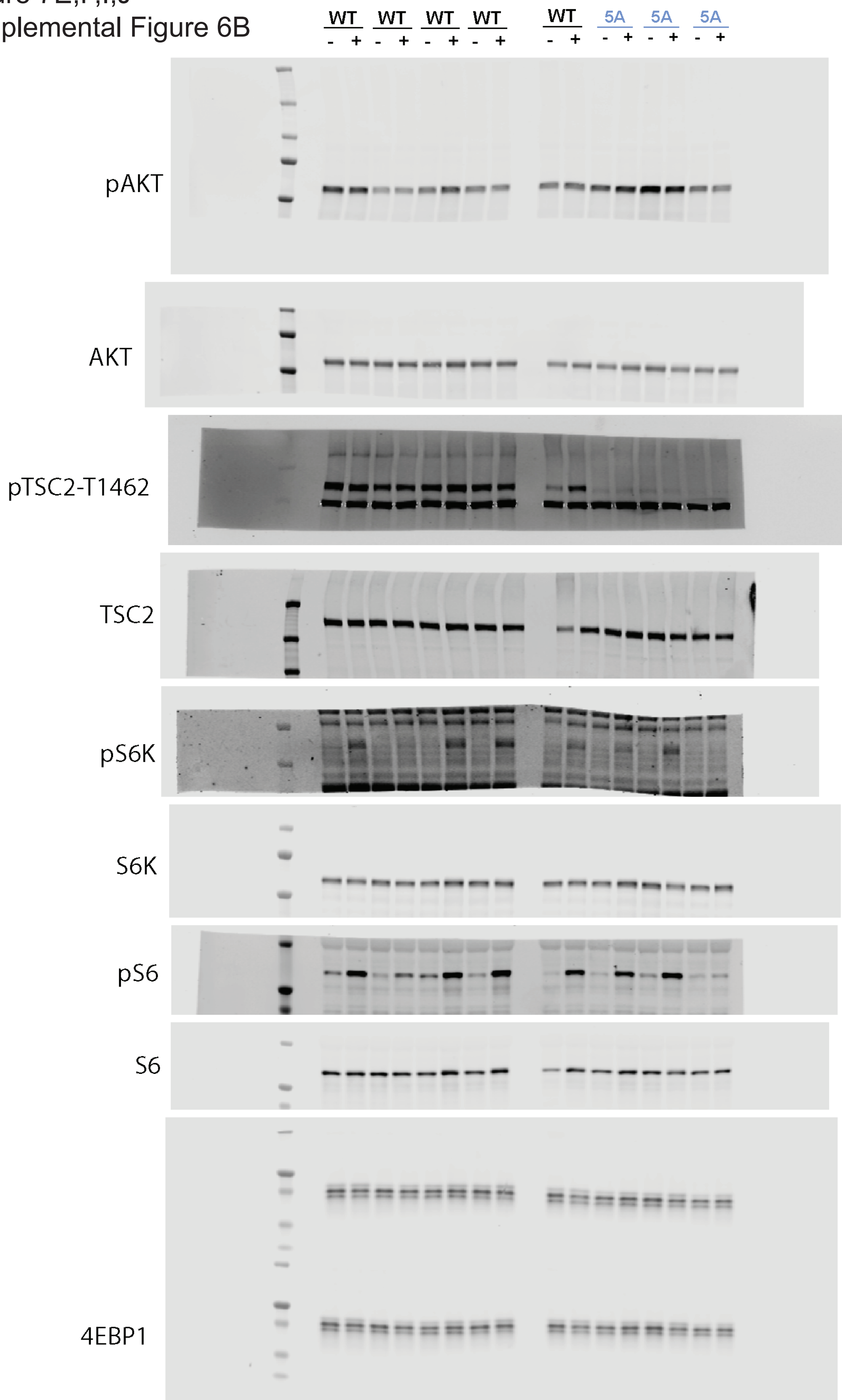

Figure 7E,F,I,J

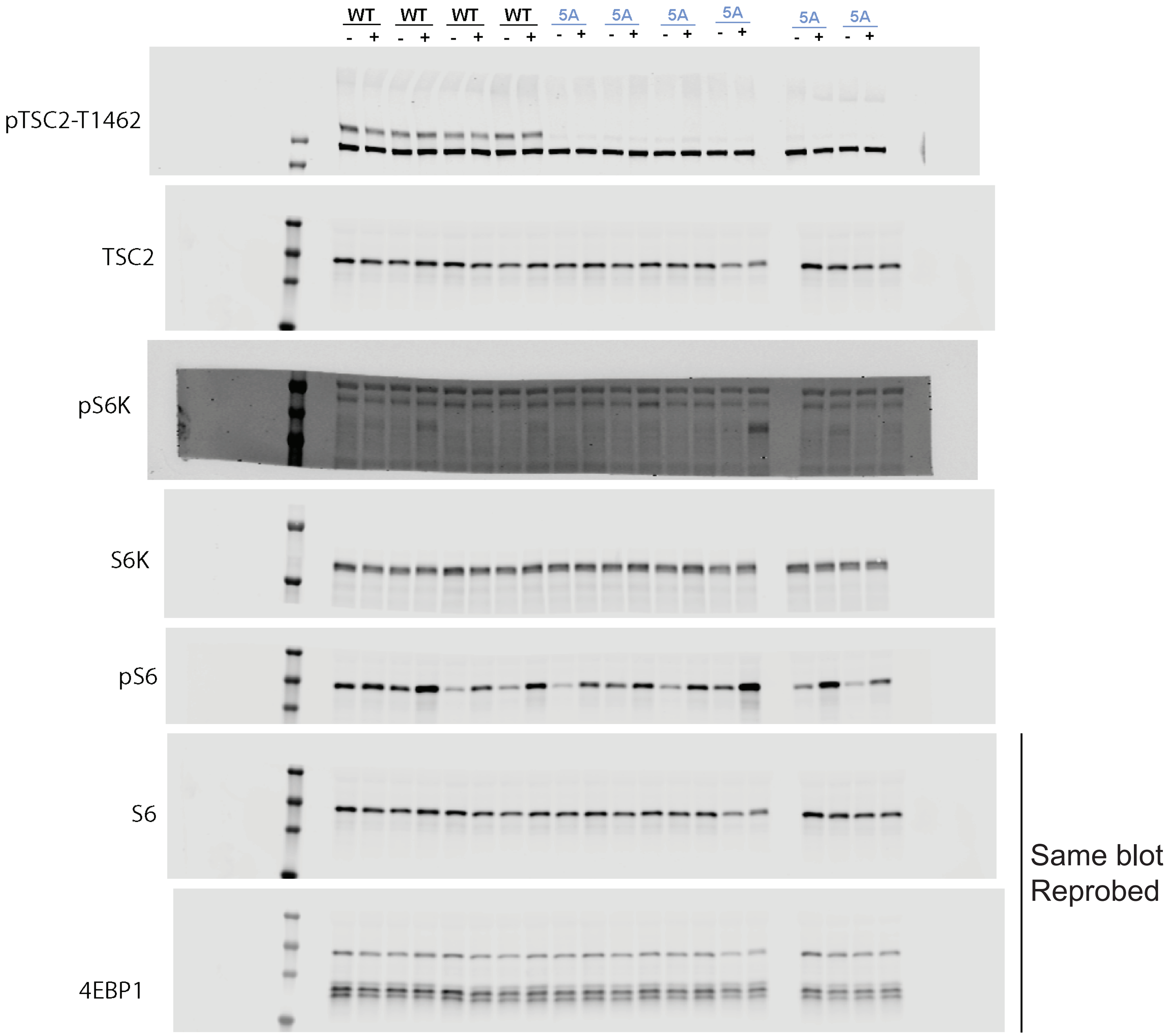

Figure 9B-D

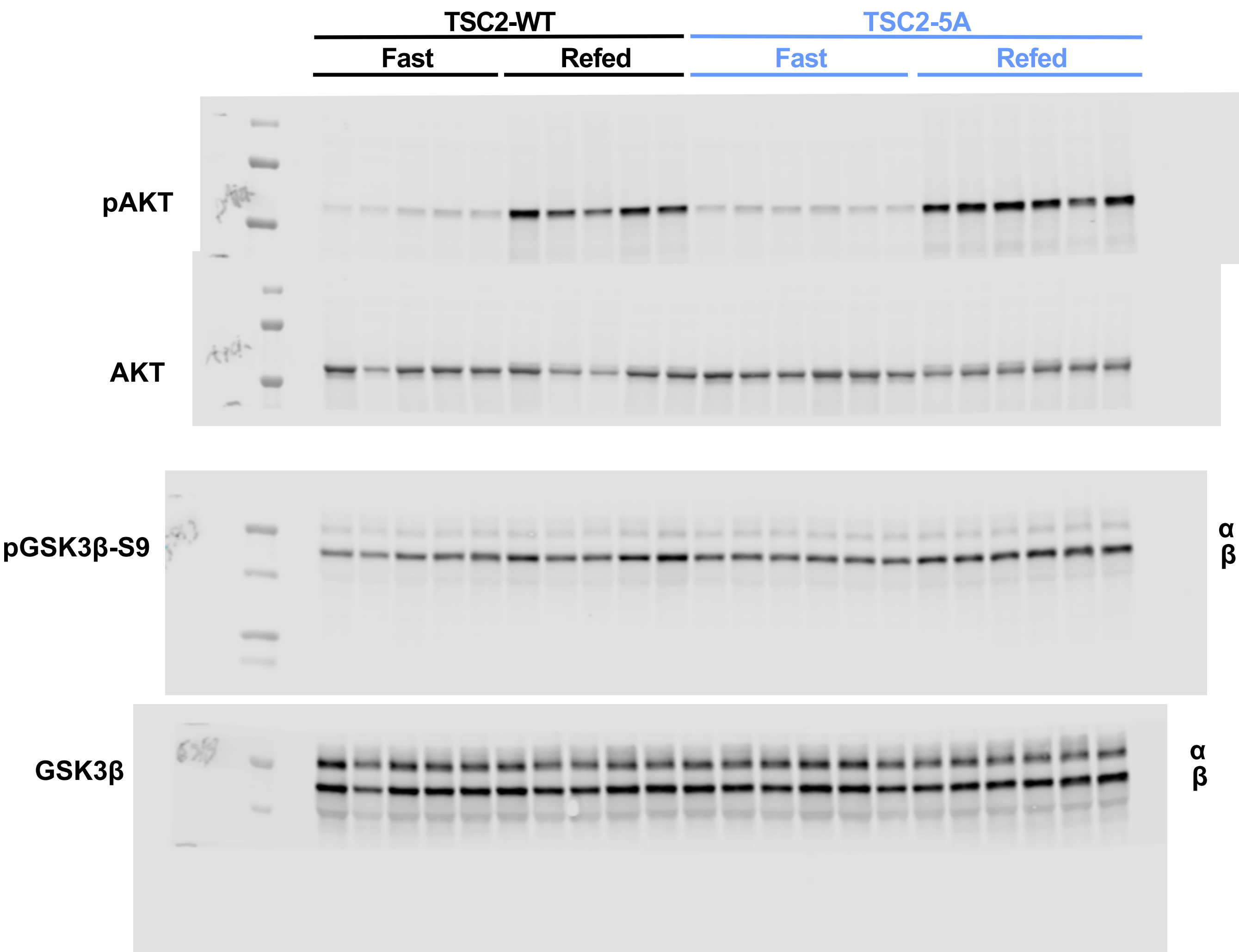

TSC2-WT
TSC2-5A
Fast
Refed
Fast
Refed
pAKT
AKT
pGSK3β-S9
α
β
GSK3β
α
β

Supplemental Figure 8A,B

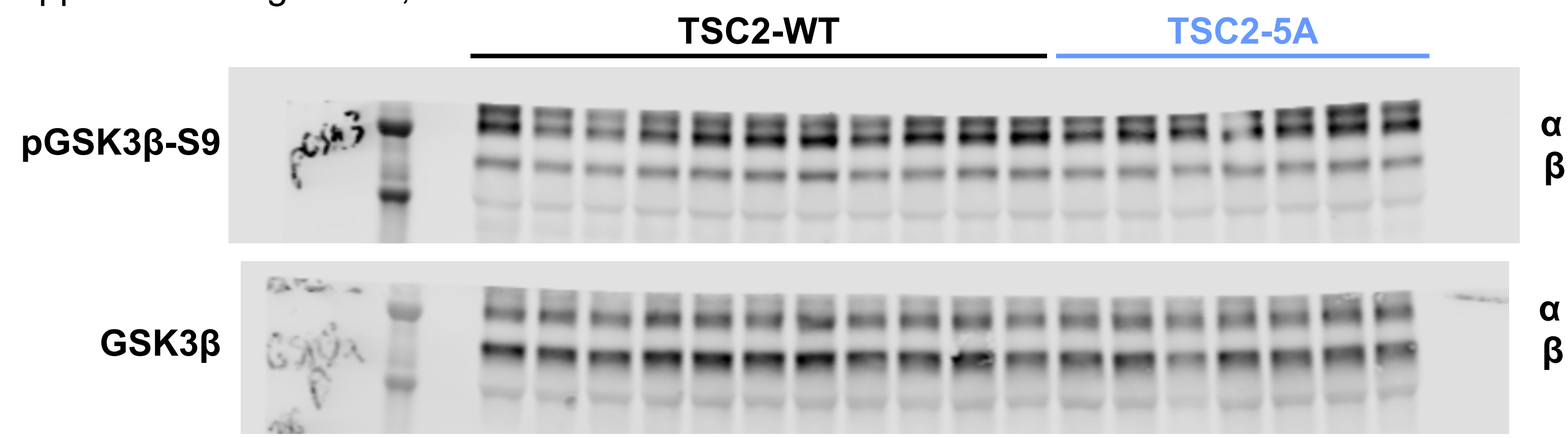

Supplement: Supplementary file 2 [file jciinsight-11-210523-s171.pdf]
